# The Potential Impact of Pre-Exposure Prophylaxis for HIV Prevention among Men Who Have Sex with Men and Transwomen in Lima, Peru: A Mathematical Modelling Study

**DOI:** 10.1371/journal.pmed.1001323

**Published:** 2012-10-09

**Authors:** Gabriela B. Gomez, Annick Borquez, Carlos F. Caceres, Eddy R. Segura, Robert M. Grant, Geoff P. Garnett, Timothy B. Hallett

**Affiliations:** 1 Department of Infectious Disease Epidemiology, School of Public Health, Imperial College London, United Kingdom; 2 Amsterdam Institute for Global Health and Development, Amsterdam, The Netherlands; 3 Instituto de Estudios en Salud, Sexualidad y Desarrollo Humano, Lima, Peru; 4 Universidad Peruana Cayetano Heredia, Lima, Peru; 5 Gladstone Institutes, University of California at San Francisco, San Francisco, California, United States of America; Harvard School of Public Health, United States of America

## Abstract

**Background:**

HIV pre-exposure prophylaxis (PrEP), the use of antiretroviral drugs by uninfected individuals to prevent HIV infection, has demonstrated effectiveness in preventing acquisition in a high-risk population of men who have sex with men (MSM). Consequently, there is a need to understand if and how PrEP can be used cost-effectively to prevent HIV infection in such populations.

**Methods and Findings:**

We developed a mathematical model representing the HIV epidemic among MSM and transwomen (male-to-female transgender individuals) in Lima, Peru, as a test case. PrEP effectiveness in the model is assumed to result from the combination of a “conditional efficacy” parameter and an adherence parameter. Annual operating costs from a health provider perspective were based on the US Centers for Disease Control and Prevention interim guidelines for PrEP use. The model was used to investigate the population-level impact, cost, and cost-effectiveness of PrEP under a range of implementation scenarios. The epidemiological impact of PrEP is largely driven by programme characteristics. For a modest PrEP coverage of 5%, over 8% of infections could be averted in a programme prioritising those at higher risk and attaining the adherence levels of the Pre-Exposure Prophylaxis Initiative study. Across all scenarios, the highest estimated cost per disability-adjusted life year averted (uniform strategy for a coverage level of 20%, US$1,036–US$4,254) is below the World Health Organization recommended threshold for cost-effective interventions, while only certain optimistic scenarios (low coverage of 5% and some or high prioritisation) are likely to be cost-effective using the World Bank threshold. The impact of PrEP is reduced if those on PrEP decrease condom use, but only extreme behaviour changes among non-adherers (over 80% reduction in condom use) and a low PrEP conditional efficacy (40%) would adversely impact the epidemic. However, PrEP will not arrest HIV transmission in isolation because of its incomplete effectiveness and dependence on adherence, and because the high cost of programmes limits the coverage levels that could potentially be attained.

**Conclusions:**

A strategic PrEP intervention could be a cost-effective addition to existing HIV prevention strategies for MSM populations. However, despite being cost-effective, a substantial expenditure would be required to generate significant reductions in incidence.

*Please see later in the article for the Editors' Summary*

## Abstract 2

Gabriela Gomez and colleagues developed a mathematical model of the HIV epidemic among men who have sex with men and transwomen in Lima, Peru to explore whether HIV pre-exposure prophylaxis could be a cost-effective addition to existing HIV prevention strategies.

## Editors' Summary

**Background:**

Without a vaccine, the only ways to halt the global HIV epidemic are prevention strategies that reduce transmission of the HIV virus. Up until recently, behavioral strategies such as condom use and reduction of sexual partners have been at the center of HIV prevention. In the past few years, several biological prevention measures have also been shown to be effective in reducing (though not completely preventing) HIV transmission. These include male circumcision, treatment for prevention (giving antiretroviral drugs to HIV-infected people, before they need it for their own health, to reduce their infectiousness) and pre-exposure prophylaxis (or PrEP), in which HIV-negative people use antiretroviral drugs to protect themselves from infection. One PrEP regimen (a daily pill containing two different antiretrovirals) has been shown in a clinical trial to reduce new infections by 44% in of men who have sex with men (MSM). In July 2012, the US Food and Drug Administration approved this PrEP regimen to reduce the risk of HIV infection in uninfected men and women who are at high risk of HIV infection and who may engage in sexual activity with HIV-infected partners. The approval makes it clear that PrEP needs to be used in combination with safe sex practices.

**Why Was This Study Done?:**

Clinical trials have shown that PrEP can reduce HIV infections among participants, but they have not examined the consequences PrEP could have at the population level. Before decision-makers can decide whether to invest in PrEP programs, they need to know about the costs and benefits at the population level. Besides the price of the drug itself, the costs include HIV testing before starting PrEP, as well as regular tests thereafter. The health benefits of reducing new HIV infections are calculated in “disability-adjusted life years” (or DALYs) averted. One DALY is equal to one year of healthy life lost. Other benefits include future savings in lifelong HIV/AIDS treatment for every person whose infection is prevented by PrEP.

This study estimates the potential costs and health benefits of several hypothetical PrEP roll-out scenarios among the community of MSM in Lima, Peru. The scientists chose this community because many of the participants in the clinical trial that showed that PrEP can reduce infections came from this community, and they therefore have some knowledge on how PrEP affects HIV infection rates and behavior in this population. Because the HIV epidemic in Lima is concentrated among MSM, similar to most of Latin America and several other developed countries, the results might also be relevant for the evaluation of PrEP in other places.

**What Did the Researchers Do and Find?:**

For their scenarios, the researchers looked at “high coverage” and “low coverage” scenarios, in which 20% and 5% of uninfected individuals use PrEP, respectively. They also divided the MSM community into those at lower risk of becoming infected and those at higher risk. The latter group consisted of transwomen at higher risk (transsexuals and transvestites with many sexual partners) and male sex workers. In a “uniform coverage” scenario, PrEP is equally distributed among all MSM. “Prioritized scenarios” cover transwomen at higher risk and sex workers preferentially. Two additional important factors for the estimated benefits are treatment adherence (i.e., whether people take the pills they have been prescribed faithfully over long periods of time even though they are not sick) and changes in risk behavior (i.e., whether the perceived protection provided by PrEP leads to more unprotected sex).

The cost estimates for PrEP included the costs of the drug itself and HIV tests prior to PrEP prescription and at three-month intervals thereafter, as well as outreach and counseling services and condom and lubricant promotion and provision.

To judge whether under the various scenarios PrEP is cost-effective, the researchers applied two commonly used but different cost-effectiveness thresholds. The World Health Organization's WHO-CHOICE initiative considers an intervention cost-effective if its cost is less than three times the gross domestic product (GDP) per capita per DALY averted. For Peru, this means an intervention should cost less than US$16,302 per DALY. The World Bank has more stringent criteria: it considers an intervention cost-effective for a middle-income country like Peru if it costs less than US$500 per DALY averted.

The researchers estimate that PrEP is cost-effective in Lima's MSM population for most scenarios by WHO-CHOICE guidelines. Only scenarios that prioritize PrEP to those most likely to become infected (i.e., transwomen at higher risk and sex workers) are cost-effective (and only barely) by the more stringent World Bank criteria. If the savings on antiretroviral drugs to treat people with HIV (those who would have become infected without PrEP) are included in the calculation, most scenarios become cost-effective, even under World Bank criteria.

The most cost-effective scenario, namely, having a modest coverage of 5%, prioritizing PrEP to transwomen at higher risk and sex workers, and assuming fairly high adherence levels among PrEP recipients, is estimated to avert about 8% of new infections among this community over ten years.

**What Do these Findings Mean?:**

These findings suggest that under some circumstances, PrEP could be a cost-effective tool to reduce new HIV infections. However, as the researchers discuss, PrEP is expensive and only partly effective. Moreover, its effectiveness depends on two behavioral factors—adherence to a strict drug regimen and continued practicing of safe sex—both of which remain hard to predict. As a consequence, PrEP alone is not a valid strategy to prevent new HIV infections. It needs instead to be considered as one of several available tools. If and when PrEP is chosen as part of an integrated prevention strategy will depend on the specific target population, the overall funds available, and how well its cost-effectiveness compares with other prevention measures.

**Additional Information:**

Please access these websites via the online version of this summary at http://dx.doi.org/10.1371/journal.pmed.1001323.

- Information is available from the US National Institute of Allergy and Infectious Diseases on HIV infection and AIDS
- NAM/aidsmap provides basic information about HIV/AIDS, summaries of recent research findings on HIV care and treatment, and a section on PrEP
- Information is available from Avert, an international AIDS charity, on many aspects of HIV/AIDS, including HIV prevention
- AVAC Global Advocacy for HIV Prevention provides up-to-date information on HIV prevention, including PrEP
- The US Centers for Disease Control and Prevention also has information on PrEP
- The World Health Organization has a page on its WHO-CHOICE criteria for cost-effectiveness

## Introduction

The use of antiretroviral (ARV) medicines for prevention of acquisition or transmission of HIV is currently a focus of policy discussions. The use of ARV drugs in HIV-uninfected individuals to prevent HIV acquisition—pre-exposure prophylaxis (PrEP)—is one of the alternatives being considered as a potential tool in the HIV prevention arsenal [1]. In 2010, the results of the first phase III clinical trial of PrEP were published: the Pre-Exposure Prophylaxis Initiative (iPrEx) study was a multinational trial of daily oral tenofovir/emtricitabine to prevent acquisition of HIV among high-risk men who have sex with men (MSM) [2]. It showed that this regime was safe and reduced the risk of HIV acquisition by 44% [2]. Consequently, the World Health Organization, the US Centers for Disease Control and Prevention, the British Association for Sexual Health and HIV, and the South African HIV Clinicians Society have published interim guidance on PrEP [3]–[6] recommending its use as part of a programme of comprehensive HIV prevention. Recently the US Food and Drug Administration approved the use of ARV drugs (tenofovir/emtricitabine, brand name Truvada) for use as PrEP among men and women [7]. Consultations are taking place to inform public health policy-makers in the development of clinical and service guidelines regarding PrEP. Additionally, adding momentum to this fast-moving field, PrEP was also found to be effective in preventing acquisition of HIV among heterosexual men and women in sub-Saharan Africa in some studies [8],[9]. However, FEM-PrEP, a trial recruiting heterosexual women in South Africa, Tanzania, and Kenya was closed prematurely last year when the data review committee stated that it would not be able to demonstrate an effect of PrEP [10]. Two further trials have tested the efficacy of 1% tenofovir gel, with somewhat inconsistent results. The CAPRISA 004 trial found a reduction in women's risk by 39% [11], while the VOICE trial's gel arm was stopped early after finding the product safe but not effective [12]. There is, therefore, a need to understand if and how PrEP could cost-effectively prevent HIV infection in specific populations within the current context of expanding access to treatment.

Although trials can demonstrate effectiveness in reducing an individual's chance of acquiring HIV, they do not show the extent to which a PrEP intervention would reduce the spread of HIV at the population level. Questions remain about how to optimally implement PrEP when multiple forms of delivery, prioritisation, and scale-up are possible in the context of other HIV prevention interventions. In response, we have built and analysed a mathematical model of the HIV epidemic among MSM and transwomen (male-to-female transgender individuals) in Lima, Peru. Mathematical models provide a framework to examine the potential impact of interventions that may inform policy development [13]. The HIV epidemic in Lima, Peru, is concentrated among MSM, comparable to most of Latin America and multiple high-income settings [14]. As such, it provides a “test case” for which there are high-quality behavioural and epidemiological data to adequately specify a mathematical model. Moreover, the majority of iPrEx participants were recruited in Peru, allowing us to include representative information on potential PrEP use. Our analysis aims to provide information to assist the process of translating recent trial results into cost-effective programmes.

## Methods

In this paper, we present the potential benefits and costs of a hypothetical PrEP intervention using a deterministic, compartmental model to represent the sexual transmission of HIV amongst MSM and transwomen in Lima, Peru. Our aim was to investigate the impact of a feasible intervention and to determine the most efficient strategies for its roll out in this population. In particular, we looked at the impact of coverage, adherence, and prioritisation on both health benefits and costs to the health system.

**Figure 1:**
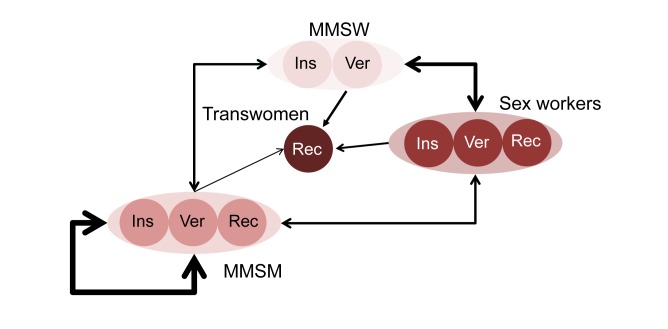
Model representation of sexual mixing and sexual positioning among MSM and transwomen. Insertive (Ins) and receptive (Rec) men always take the insertive and receptive role during anal sex, respectively. Versatile (Ver) men take either the insertive or the receptive role during anal sex. Arrows indicate sexual partnerships being formed between individuals within groups—the width shows the number of partnerships, and the direction illustrates sexual positioning (from insertive towards receptive). For each category, insertive men will form partnerships only with receptive men or versatile men (the latter in a receptive role); versatile men will form partnerships with insertive men, versatile men, or receptive men, depending on their role per sex act; receptive men will form partnerships only with insertive men or versatile men (the latter in an insertive role).

Lima has a diverse MSM population, which we defined as men or transwomen who have reported a sexual partnership with a man in the last 12 mo. To represent HIV spread in the model, we defined four interacting groups: men who mostly have sex with women (MMSW), men who mostly have sex with men (MMSM), sex workers, and transwomen at higher risk (including transsexuals and transvestites who have a large number of partners on average). These categories are intended to represent a broad spectrum of sexual identities, orientations, and behaviours encompassing variation in numbers of partners, types of partnerships formed (stable, casual, commercial), condom use, and sex work (defined as the exchange of anal sex for money, drugs, gifts, or favours). Our subgroup definitions followed the classification commonly used in research studies in Peru [14]. Some groups were further subdivided into mutually exclusive compartments according to their sexual positioning in anal sex (insertive, receptive, or versatile, i.e., practicing both insertive and receptive anal sex), resulting in a total of nine groups (Figure 1; more information on the group definitions can be found in Text S1). To estimate the distribution of MSM into the different risk groups, we reviewed studies describing the proportion of MSM reporting sex with a woman in the last year and/or the proportion of MSM self-identified as heterosexuals or bisexuals (for MMSW), as homosexuals/gay (for MMSM), and as transgender, transvestite, or transsexual. To estimate the proportion of sex workers, we subtracted the proportions of MMSW, MMSM, and transwomen from the total. We then compared this estimate against reported proportions of MSM involved in sex work during the last year [15]–[20]. Frequency of partnership formation was based on reported number of sexual partners from published studies and unpublished data from studies of transwomen (transwomen study funded by amfAR) [21] and MSM (CPOS study; C. Caceres and E. R. Segura, unpublished data). Stable, casual, and commercial partnerships were defined by frequencies of sex acts. For the versatile group, the total number of sex acts was divided into a proportion of insertive or receptive acts. Condom use was estimated from reported condom use data at last sex act for different partnership types. If data were available for both the insertive and receptive partners, the probability of using a condom was determined by the receptive partner. Receptive partners have a higher probability of infection than insertive partners, and condom use thus has a higher impact on the receptive partner. HIV prevalence in Lima among the general population is low, and therefore the risk of transmission to MSM and transgender people from other sources was not included (tables detailing contact patterns by type of partnership, HIV prevalence by group, group distributions, and demographic and behavioural parameters used in the model can be found in Text S1).

Following previous work, the natural course of HIV infection was represented as four phases of disease progression defined by duration and infectiousness: acute infection, latent phase, pre-AIDS, and AIDS (see Text S1) [22]–[25]. A proportion of individuals initiate antiretroviral treatment (ART) rather than progress to pre-AIDS, depending on the coverage level. ART is assumed to reduce infectiousness and extend survival [26].

**Figure 2:**
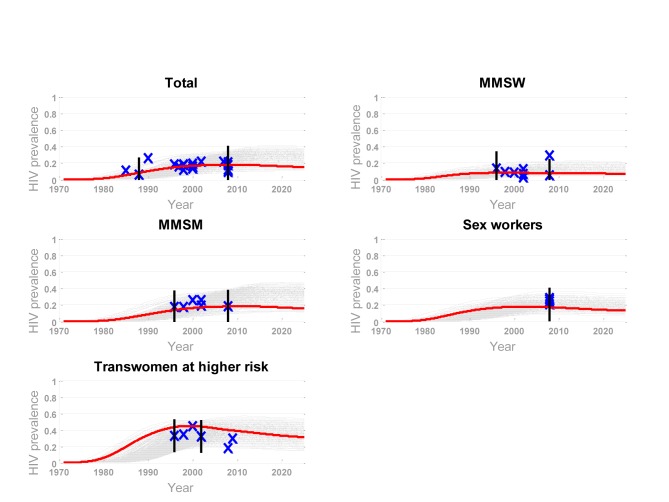
Selected model runs and best fit based on prevalence data for four subgroups and the overall population. Grey shading represents selected runs; red lines represent the best fit. The runs were selected based on bounds (black bars) defined from prevalence data (blue x's) for four subgroups and the overall population.

Three sources of information were used to calibrate the model: reported behaviour, HIV natural history, and data on prevalence and incidence for MSM and transwomen subpopulations in Lima (from the Peruvian Ministry of Health and published studies; these sources and values are detailed in Text S1). We used a quasi-Bayesian procedure to combine these sources of information and account for uncertainty around parameter estimates [27],[28]. This procedure consists in allowing certain parameters to vary within a specified prior distribution reflecting the uncertainty of the estimates. The parameters allowed to vary describe the risk distribution in the population and risk behaviours (presented in Text S1). These are specific to each setting and are most vulnerable to bias in the sampling method and to reporting bias. The sampling of parameter values was carried out using Latin hypercube sampling [29]. Each model run is the result of a different combination of parameter values. Prior limits on prevalence are defined in order to select plausible runs, and then the best fitting set of parameters is determined using the log likelihood (details on prior limits chosen are given in Text S1). Of 10,000 runs performed, 449 were selected and are shown in Figure 2, together with the best fit based on prevalence data for the four subgroups (MMSW, MMSM, sex workers, and transwomen at higher risk) and the overall population. This best fit was then used for the primary analyses. To ensure it was representative of the most likely epidemiological scenario, we explored the uncertainty due to the epidemiological assumptions and compared the results obtained using the parameter set corresponding to the best fit to those from all other runs selected in the Bayesian process. The distribution of infections averted obtained from all selected runs and the 50 best fits are presented in Text S1 and compared to the estimates obtained from the best fit for the most relevant scenarios.

### Assumptions about the Pre-Exposure Prophylaxis Intervention in the Model

PrEP effectiveness in the model is assumed to result from the combination of a reduction in susceptibility to HIV infection during a PrEP-protected sex act (“conditional efficacy” parameter) and a proportion of sex acts protected (adherence parameter). We aimed to reflect a daily regimen of PrEP and an overall effectiveness equivalent to that of the iPrEx trial, 44% (95% CI 15–63) [2]. In a retrospective analysis of data from the iPrEx trial, for those with detectable levels of drug in their blood samples, HIV incidence was reduced by 92% (95% CI 40–99). We interpreted these findings to show an efficacy of PrEP that varies with individual-level adherence, in accordance to results presented recently in which biological determinants did not explain the differential efficacy observed in the iPrEx trial [30]. We refer to this parameter as a “conditional efficacy” in the model to reflect an assumption of high intrinsic efficacy of PrEP (equivalent to 92%) but large population-level heterogeneity in adherence behaviours. Therefore, we divided PrEP-users into three adherence groups using point estimates: good (95%), average (45%), and poor (15%). The proportion in each group was altered to reflect three adherence profiles: the “iPrEx adherence” profile, for which the proportions were as observed in the trial, and the “less adherence” and “more adherence” scenarios. The adherence profile in the “more adherence” scenario corresponds to the hypothesis being tested in the open label extension of the iPrEx trial looking at a possible increase in use of the study drug by participants due to the participants' knowledge of receiving an active drug that provides some protection against HIV infection. For all scenarios, when both the conditional efficacy and adherence profiles are combined, the functional effectiveness calculated reproduces the effectiveness observed in the iPrEx trial, 44% (95% CI 15–63) [2]. Table 1 shows the assumptions made for conditional efficacy and adherence, as well as the resulting functional effectiveness for each profile.

**Table 1:** Scenario definitions: impact of PrEP by adherence and functional effectiveness.

Efficacy/Adherence	Adherence		
	iPrEx Adherence	More Adherence	Less Adherence
Conditional efficacy	0.92 (0.4–0.99)	0.92 (0.4–0.99)	0.92 (0.4–0.99)
Adherence 1^a^	0.95	0.95	0.95
Adherence 2^a^	0.40	0.40	0.40
Adherence 3^a^	0.15	0.15	0.15
Proportion in adherence group 1	0.50	0.60	0.30
Proportion in adherence group 2	0.00	0.10	0.10
Proportion in adherence group 3	0.50	0.30	0.60
Functional effectiveness	0.52	0.62	0.35

All values are proportions. Functional effectiveness is a function of the probability of transmission, the intrinsic efficacy, the adherence to PrEP, and its distribution, affecting only unprotected sex acts, which in turn are dependent on the number of partners, average condom use, and number of sex acts per partner (all values derived from iPrEx data [2]).

a Adherence 1, 2, and 3 refer to the proportion of doses taken consistently by an individual. The proportion in each adherence group is the population distribution (the number of people in each adherence group). This reflects the data reported in the iPrEx study: a distribution of participants by ranges of adherence. We included a point estimate within those ranges.

We constructed two scenarios for overall scale-up of PrEP programmes: (i) “low coverage” and (ii) “high coverage”, whereby 5% and 20% of uninfected individuals use PrEP, respectively. In both scenarios, scale-up begins in 2012, and coverage is reached in 5 y and maintained thereafter for an additional 5 y. We quantified the contribution of each subpopulation to the number of future HIV infections and the relative impact of PrEP if used in each group separately to identify key points for an intervention. We looked at the impact of distributing a fixed amount of PrEP (25,000 person-years, corresponding to a 10% resource allocation of the national prevention programme to PrEP [31]) to each group in isolation. PrEP could then be distributed evenly to all MSM and transwomen (“uniform coverage”), or certain key groups could be prioritised for PrEP allocation (“prioritisation”). Prioritised scenarios could result in “some prioritisation”, where a higher coverage is achieved in key populations such as the transwomen at higher risk and sex worker groups (but no more than 50% coverage) than in MMSW and MMSM, or in “high prioritisation”: once 90% of the transwomen at higher risk group receives PrEP, the residual amount (to achieve 5% or 20% overall coverage) is divided among the three other populations, prioritising sex workers over MMSW and MMSM. Table 2 shows the coverage assumptions of PrEP for each scenario.

**Table 2:** Scenario definitions: impact of PrEP by coverage and prioritisation.

Distribution	Subgroup	Coverage	
		Low Coverage (5%)	High Coverage (20%)
Uniform coverage	Overall	0.05	0.2
	MMSW	0.05	0.2
	MMSM	0.05	0.2
	MSW	0.05	0.2
	Trans	0.05	0.2
Some prioritisation	Overall	0.05	0.2
	MMSW	0	0.46
	MMSM	0	0.09
	MSW	0.26	0.5
	Trans	0.5	0.5
High prioritisation	Overall	0.05	0.2
	MMSW	0	0.22
	MMSM	0	0.04
	MSW	0.11	0.9
	Trans	0.9	0.9

All values are proportions. MSW, men sex workers; trans, transwomen at higher risk.

### Cost-Effectiveness Calculations

We estimated the annual operating costs of providing a hypothetical PrEP intervention to individuals, from a health provider perspective, based on the US Center for Disease Control and Prevention interim guidelines for PrEP [3]. In accordance with these clinical recommendations, we included costs for the following procedures: HIV testing before starting PrEP, HIV testing every 3 mo during use of PrEP, HIV confirmatory testing if an individual is found positive, one creatinine/blood urea nitrogen test per year during PrEP use, outreach and counselling services, and condom and lubricant promotion and provision. The estimation includes additional costs for human resources, but it does not include costs related to HIV treatment after infection, resistance testing, or testing and treatment of other sexually transmitted infections. We included a 5% extra cost to allow for the creation of an enabling environment at the project level, programme management costs, or monitoring and evaluation costs at the project level [32]. This 5% mark-up was chosen based on the budget reported for management, monitoring, and evaluation of HIV programmes at the national level [31]. We did not include indirect costs such as potential earning forgone by patients or carers. We present all costs as unadjusted market prices, specific to Peru [31],[33],[34], and calculated for 10 y (i.e., the duration of our simulated intervention).

We estimated the unit cost of a PrEP intervention to be between US$525 and US$830, of which the main component was the cost of PrEP drugs (over two-thirds of the total estimate). The cost of drugs used for PrEP was set to be between US$420 and US$600 per year based on cost data provided by Gilead (for one bottle [1 mo] supply: Viread, US$30, and Truvada, US$45, plus a 10% to 15% distributor mark-up per bottle) [33].

For the cost-effectiveness analysis, our principal epidemiological outcome was cost per disability-adjusted life year (DALY) averted. PrEP implementation scenarios were assessed against a “no PrEP intervention” scenario. Total number of DALYs averted was then calculated using the number of infections averted in each scenario. The uncertainty due to the epidemiological assumptions was represented by adding credible intervals in our primary analysis. These credible intervals correspond to the model runs estimating the maximum and minimum impact of PrEP, in terms of infections averted per person-year on PrEP. The estimated number of DALYs associated with one HIV infection averted was calculated as the sum of the number of years of life lost and the number of years lost due to disability using published methods [34],[35]. These calculations included weights for Peruvian life expectancy, age, future time, and disability. Duration of disability was calculated on the basis of local clinical data for HIV-positive patients presenting to care [36], and the disability weights for HIV-related conditions were obtained from the Global Burden of Disease study [37]. We estimated approximately 12.3 discounted DALYs averted per infection averted, including access to ART for 80% of infected individuals. When we excluded the age weighting function, we estimated 11.5 discounted DALYs averted. This is the equivalent of 27.09 (and 27.12 when we exclude the age weighting function) DALYs averted per infection averted if no discounting is assumed, comparable to other estimates in the HIV literature [38],[39]. All details of costs and DALY estimate calculations are given in Text S1. Future costs, savings, and health gains were discounted at a rate of 3%. See Figures S3, S5, S6, S7, S10, and S11 and Table S1 for a sensitivity analysis of all our results if downstream costs of treatment averted are included in the evaluation. The cost of ART for this analysis varied from US$1,000 [40] to US$3,500 [38].

Currently, there is a subjective selection of cost-effectiveness thresholds in the literature [41]. We refer to two threshold systems commonly used. The World Health Organization Choosing Interventions That Are Cost-Effective (WHO-CHOICE) initiative considers an intervention to be (1) very cost-effective, if its cost is less than the gross domestic product (GDP) per capita (<US$5,401) per DALY averted; (2) cost-effective, if it costs between one and three times the GDP per capita (US$5,401 to US$16,203) per DALY averted; or (3) not cost-effective, if it costs more than three times the GDP per capita (>US$16,203) per DALY averted. The GDP values are those estimated for Peru in 2010 [34],[42]. The second threshold system we used involves more conservative cutoff points suggested by the World Bank in 1993 for middle-income countries [43]: <US$100 per DALY averted to reflect a highly cost-effective intervention, between US$100 to US$500 for a cost-effective intervention, and >US$500 for an intervention to be considered not cost-effective. These World Bank cutoff points were considered in the analysis inflated to their 2010 equivalents—<US$149 for a highly cost-effective intervention and <US$745 for a cost-effective intervention.

### Analysis

We quantified the impact, cost, and cost-effectiveness of different patterns of PrEP use across the population, identifying dependencies on PrEP conditional efficacy, coverage, scale-up period, prioritisation of key groups, and adherence. The impact of risk compensation, i.e., where those on PrEP reduce condom use, was investigated. We then used the model to find intervention scenarios that would result in reducing the number of new infections by one-third, representing a relevant planning target for Lima.

## Results

### Key Groups to Prioritise among MSM and Transgender People

**Figure 3:**
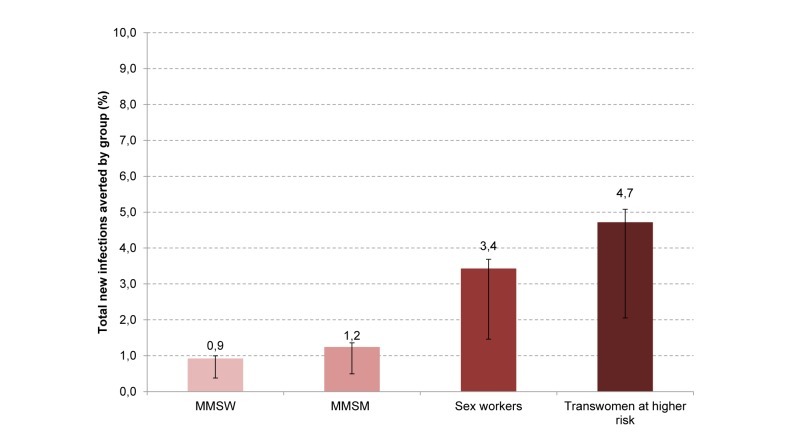
Proportion of total new infections averted over 10 y by giving 25,000 PrEP person-years to each subgroup in isolation. The error bars reflect the iPrEx efficacy estimate of 92% (95% CI 40–99).

To establish an appropriate set of strategies for using PrEP, it is important to identify which groups to prioritise. Individuals who might be a priority could be determined by (1) the number of similar people and the ease of reaching them, (2) their own risk of HIV, and (3) their relative contribution to onward transmission. In Lima, the majority of MSM are MMSM (approximately 70%) and MMSW (approximately 15%), but both groups experience a relatively low risk of infection (2.5% and 1.0% modelled incidence in 2010, respectively). Transwomen at higher risk (approximately 5%) and sex workers (approximately 10%) are smaller groups, but are at higher risk of infection (7.3% and 3.1% modelled incidence in 2010, respectively). A fixed amount of PrEP (25,000 person-years) would allow covering 60% of transwomen at higher risk, 20% of sex workers, 3% of MMSM, and 16% of MMSW over 10 y. Allocated only to transwomen at higher risk or sex workers, 25,000 person-years of PrEP would be expected to avert, respectively, 4.7% or 3.4% of infections over 10 y in the whole population, whereas the same amount of PrEP allocated to MMSW or MMSM would avert fewer infections: 0.9% or 1.2%, respectively (Figure 3). For these reasons, in this epidemic context, strategies that prioritise transwomen at higher risk and sex workers could be expected to have higher impact for the same cost, including amongst those not taking PrEP, because of the prevention of downstream infection.

### Potential Impact of PrEP Programmes as a Function of Adherence, Coverage, and Prioritisation Strategies

**Figure 4:**
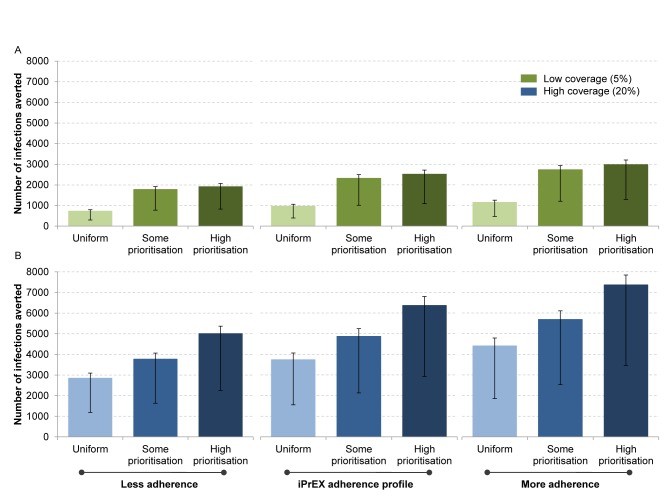
Estimated impact of PrEP with respect to coverage, adherence, and prioritisation of key populations. The error bars reflect the uncertainty in the iPrEx efficacy estimate of 92% (95% CI 40–99). In this comparison, we show two scenarios (low coverage in green [A]; high coverage in blue [B]) including three prioritisation strategies—uniform, where the coverage is the same in each subgroup; some prioritisation, where higher coverage is achieved in the transwomen at higher risk and sex worker populations (but no more than 50% covered) than in MMSM and MMSW; and high prioritisation, where 90% of transwomen at higher risk and 11% of sex workers receive PrEP in the low coverage scenario, or 90% of transwomen at higher risk, 90% of sex workers, 3.9% of MMSM, and 21.5% of MMSW receive PrEP in the high coverage scenario.

We estimated the impact of a PrEP intervention over 10 y under a range of implementation strategies. Figure 4 shows the number of infections averted over 10 y (number of infections averted per 1,000 person-years on PrEP and the proportion of infections averted over 10 y are shown in Figures S1 and S2). For a modest programme of 5% PrEP coverage, over 8% of infections could be averted with an adherence level equivalent to the one observed in the iPrEx trial and with prioritisation (high prioritisation scenario) of the groups most likely to become infected. Prioritising PrEP to sex workers and transwomen rather than achieving a uniform coverage increases the impact of interventions. This is particularly the case when overall coverage is low. For example, for an iPrEx adherence profile, with a coverage of 5%, the number of infections averted with a uniform coverage strategy (970 [range 394–1,060]) more than doubles by prioritising PrEP in a high prioritisation scenario (2,519 [range 1,086–2,713]). Increased individual adherence to PrEP increases its estimated impact at the population level. With higher coverage levels, the impact of PrEP interventions is greater but the relative benefit is reduced (the numbers of infections averted per year of PrEP decreases). This is because, in the high coverage scenario, person-years of PrEP become relatively less efficiently allocated to individuals with low risk.

### Cost-Effectiveness of PrEP interventions

**Figure 5:**
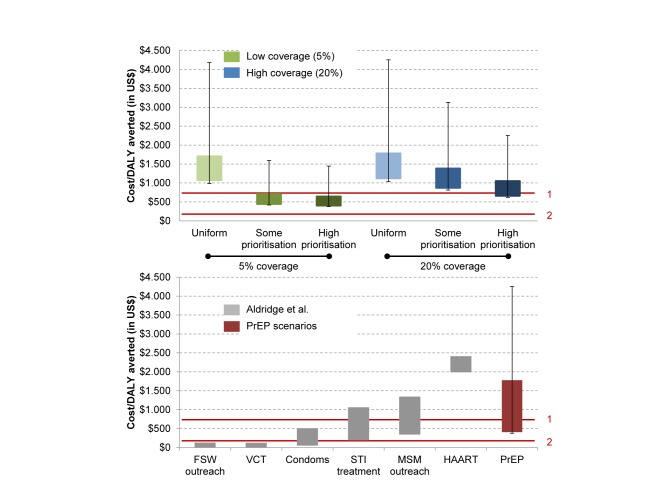
Cost-effectiveness of PrEP, estimated as cost per DALY averted. The top panel shows PrEP cost per DALY averted with regard to coverage levels and strategies. The bottom panel shows a comparison of PrEP cost per DALY averted with other HIV prevention strategies. The error bars reflect the uncertainty in the iPrEx efficacy estimate of 92% (95% CI 40–99). The height of the bars shows the variation due only to the cost assumptions of one person-year on PrEP. In the top panel, the iPrEx adherence profile is used for the scenarios. In green: low coverage scenario: 5%; in blue: high coverage scenario: 20%. In the bottom panel, the variation in cost for the interventions reflects different coverage strategies assumed and the uncertainty in the costing. Values from Aldridge et al. [38] presented in this paper were calculated using assumptions from the GOALS model on coverage and intervention design (see also Stover et al. [54]). PrEP estimated cost/DALY was calculated for both uniform and high prioritisation strategies (including 5% and 20% coverage scenarios). The red lines correspond to (1) the World Bank threshold for a cost-effective intervention, <US$745/DALY averted, and (2) the World Bank threshold for a highly cost-effective intervention, <US$149/DALY averted. FSW, female sex worker; HAART, highly active ART; STI, sexually transmitted infection; VCT, voluntary counselling and testing.

The cost per DALY averted was quantified for PrEP interventions assuming the iPrEx profile of adherence (Figure 5, top panel). Across all scenarios the highest estimated cost per DALY averted (a uniform strategy at a 20% coverage level, US$1,126–US$1,780 [uncertainty due to PrEP conditional efficacy: US$1,036–US$4,254) is below the WHO-CHOICE threshold for a cost-effective intervention for Peru (<US$5,401/DALY averted) [34], while only prioritisation scenarios (some prioritisation and high prioritisation) at low coverage, and the low bound of a high prioritisation scenario at high coverage, are likely to be cost-effective using the more conservative threshold suggested by the World Bank (<US$745/DALY averted) [43]. None of the scenarios appear to be cost-effective when the lower bound of PrEP conditional effectiveness is included in the analysis, nor are the scenarios considered very cost-effective using the World Bank threshold of <US$149/DALY averted. However, the cost per DALY averted is substantially reduced with a high degree of prioritisation. It could be higher in larger PrEP programmes (with 20% rather than 5% coverage). In Table 3, we present the cost-effectiveness results for our six main scenarios, together with the total cost over 10 y for each scenario. For the same total investment, prioritisation improves cost-effectiveness. In Table S1, we include the downstream ARV costs averted. PrEP interventions could potentially be cost-effective in most scenarios, and cost-saving in some, if the treatment costs of infections averted are included (see Figures S5, S6, S7). Figure 5 (bottom panel) compares the cost-effectiveness of PrEP to that estimated for other prevention interventions in Peru [38]—the estimates for other interventions were derived from Aldridge et al. [38], specific to Peru. Figure 5 shows that, depending on the implementation strategy, PrEP could be as cost-effective as treatment for sexually transmitted infections, MSM outreach, or highly active ART. It also suggests that PrEP should be considered as an additional intervention available as part of a comprehensive combination HIV prevention approach. An alternative representation of the information in Figure 5 can be found in Figures S4, S5, S6.

**Table 3:** Cost-effectiveness and total cost of PrEP over 10 y.

Distribution	Coverage^a^ (Proportion)	Total Person-Years^b^	Cost/DALY: Cost Assumptions Only^c^ (US Dollars)	Cost/DALY: Total Uncertainty^d^ (US Dollars)	Total Cost^e^ (US Dollars)
Uniform	0.05	45,325	1,076–1,702	419–4,182	23,795,696–37,619,863
Uniform	0.20	182,596	1,125–1,779	428–4,254	95,863,381–151,555,440
Some prioritisation	0.05 (0, 0, 0.26, 0.5)	44,925	447–707	159–1,596	23,852,467–37,709,615
Some prioritisation	0.20 (0.04, 0.08, 0.5, 0.5)	187,116	886–1,400	397–3,133	96,398,735–152,401,810
High prioritisation	0.05 (0, 0, 0.11, 0.9)	44,002	403–637	163–1,553	23,877,220–37,748,747
High prioritisation	0.20 (0.21, 0.03, 0.9, 0.9)	183,851	665–1,052	310–2,258	96,071,585–151,884,601

Downstream ARV costs averted not included.

a Coverage in the uniform distribution strategy is equal in all subgroups (MMSW, MMSM, sex workers, and transwomen at higher risk). The coverage showed in both strategies involving prioritisation (some and high) are given as overall population coverage and, in parentheses, the coverage in each subpopulation (MMSW, MMSM, sex workers, transwomen at higher risk).

b Total number of person-years on PrEP over 10 ys.

c Variation in the cost/DALY due to cost assumptions only (corresponding to the height of the coloured bars in the top panel of Figure 5).

d Variation due to total uncertainty, including both cost and PrEP efficacy assumptions as well as epidemiological assumptions.

e Total cost of PrEP intervention over 10 y; the range reflects the uncertainty of PrEP programme costs only.

### Behaviour Change Associated with PrEP Use

**Figure 6:**
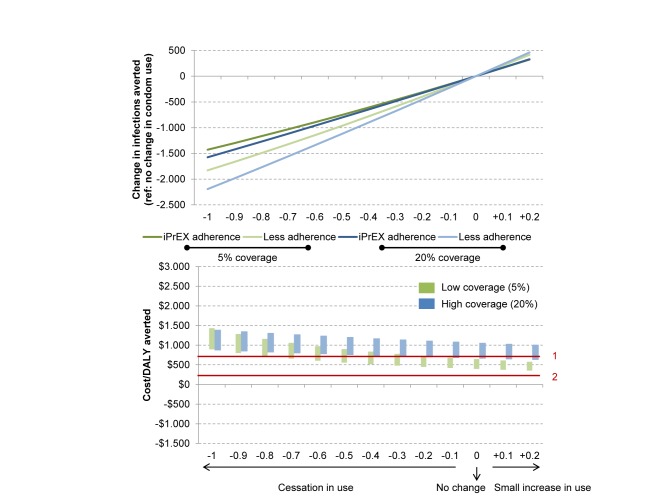
Population impact and cost of PrEP with respect to changes in condom use for a high prioritisation strategy. The top panel shows behaviour change and population impact of PrEP. The bottom panel shows behaviour change and PrEP cost per infection averted. This figure assumes there is no correlation between adherence and risk compensation. We explore this issue separately in Figure S12. The iPrEx adherence profile was used for these scenarios. In green: low coverage scenario: 5%; in blue: high coverage scenario: 20%. The red lines correspond to (1) the World Bank threshold for a cost-effective intervention, <US$745/DALY averted, and (2) the World Bank threshold for a highly cost-effective intervention, <US$149/DALY averted. The height of the bars shows the variation due only to the cost assumptions of one person-year on PrEP.

A major issue in considering the impact of PrEP interventions is whether individuals using PrEP will reduce how often they use condoms. We estimated the impact of PrEP programmes making different assumptions about condom use changes by individuals on PrEP, ranging from complete cessation of use (−100% condom use), to a small increase in use (+20% condom use). In Figure 6, we show how number of infections averted and the cost per DALY averted are affected by these changes, in the context of a low or high coverage intervention with a high degree of prioritisation (plots for a high prioritisation strategy including downstream ARV costs are given in Figure S8; results for other prioritisation strategies including and excluding downstream ARV costs are given in Figures S9, S10, S11, S12). Compared with no change in behaviour, a complete cessation of condom use among those on PrEP reduces the number of infections averted over 10 y by between 1,500 (32%) and 2,250 (45%), and increases the cost per DALY averted by US$200–US$315 for a high coverage programme (blue bars). The effect is greater for a low coverage programme (green bars). This is because only those at highest risk of acquiring and transmitting infection are receiving PrEP, such that each breakthrough infection has greater potential to cause onward epidemic spread. The potential for a PrEP intervention to have the net effect of generating more new infections is theoretically possible but highly unlikely, requiring that the lowest empirical estimate of intrinsic efficacy of PrEP be true (40% is the lower bound of the confidence interval of effectiveness among participants with drug levels in their blood [95% CI 40–99]) and that all users reduce their condom use by at least 50% (results not shown). We further explored two different scenarios where behaviour change was correlated with adherence and found that the effect on HIV incidence of behavioural change in those with high adherence is only modest (because if individuals do consistently take the PreP pills, then the degree of protection is very high). The effect of behaviour change on incidence is greater if those with bad adherence are found to change their behaviour (Figure S12). Nevertheless, communication programmes to limit risk compensation may be a cost-effective complement to PrEP interventions.

### Averting One-Third of New Infections Using PrEP

Finally, we identify alternative strategies that would be expected to reduce the number of infections over 10 y by one-third (Table 4). With a rapid scale-up of 2 y and a highly prioritised strategy, a minimum of 435,000 PrEP person-years would be required, at an estimated cost of US$196 million to US$310 million over 10 y. The same impact would be generated with a slower scale-up and a uniform distribution, but at a higher cost: US$277million to US$439 million over 10 y. With all strategies, modelling a longer period to reach an intended coverage level increases the amount of person-years on PrEP needed. The total cost and cost-effectiveness of different PrEP strategies to avert one-third of infections, including downstream ARV costs averted, are shown in Table S1. While the resources needed are still important in these scenarios, the cost-effectiveness is greatly improved.

**Table 4:** Scenarios to achieve one third of new infections averted over 10 y.

Distribution	Scale-Up	Coverage^a^ (Proportion)	Total Person-Years^b^	Cost/DALY^c^ (US Dollars)	Total Cost^d^ (US Dollars)
Uniform	Two years	0.46	597,165	1,189–1,880	269,888,000–426,680,500
Uniform	Five years	0.57	625,155	1,263–1,996	277,445,500–438,628,500
Some prioritisation	Two years	0.40 (0.26/0.38/0.63/0.63)	521,040	1,046–1,654	235,447,000–372,232,000
Some prioritisation	Five years	0.54 (0.51/0.51/0.7/0.7)	591,730	997–1,879	262,597,000–415,153,500
High prioritisation	Two years	0.34 (0.27/0.22/0.9/0.9)	433,810	870–1,375	195,959,000–309,802,000
High prioritisation	Five years	0.49 (0.42/0.41/0.9/0.9)	535,155	1,073–1,696	237,555,000–375,563,000

a Coverage in the uniform distribution strategy is equal in all subgroups (MMSW, MMSM, sex workers, and transwomen at higher risk). The coverage showed in both strategies involving prioritisation (some and high) are given as overall population coverage and, in parentheses, the coverage in each subpopulation (MMSW/MMSM/sex workers/transwomen at higher risk).

b Total number of person-years on PrEP over 10 y.

c The range represents the variation observed in the estimated costs per PrEP person-year.

d Total cost of PrEP intervention over 10 y; the range represents the variation observed in the estimated costs per PrEP person-year.

## Discussion

We estimated the potential impact of PrEP under different intervention scenarios, examining the relative importance of implementation strategies and individual adherence. Our model shows an important epidemiological impact of PrEP use, largely driven by the characteristics of the implementation programme—PrEP conditional efficacy, coverage, prioritisation strategy, and time to scale up—and risk compensation behaviour.

Overall, if downstream ARV costs averted are not included, PrEP is predicted to be cost-effective in this population by the WHO-CHOICE guidelines, and borderline cost-effective by the more conservative World Bank guidelines, if the intervention is prioritised to those most vulnerable to infection. If downstream ARV costs are included, most PrEP scenarios are found to be cost-effective. This argues for PrEP to be considered among the set of efficacious and cost-effective interventions that could be included in a comprehensive combination of HIV prevention interventions for MSM in this setting. Consistent with the significant heterogeneity in risk behaviours among MSM and transwomen at higher risk, our results show that prioritising people in higher risk groups (e.g., transwomen at higher risk) when implementing PrEP would have the largest impact. While risk compensation could reduce the impact of PrEP, our results suggest that, under a range of assumptions, PrEP is still likely to be cost-effective.

A higher coverage strengthens the impact on incidence over 10 y, as does a rapid scale-up of the intervention. However, implementation of PrEP in isolation to a scale that it would arrest the epidemic would require more resources than have been available. To reduce new infections by one-third, the total cost of introducing PrEP will amount to at least US$20 million on average per year (over US$200 million over 10 years). As a comparison, total HIV spending (prevention, treatment, and care) in Peru for the year 2009 was reported to be just over US$40 million [44]. Furthermore, while Peru has been an important aid recipient from The Global Fund to Fight AIDS, Tuberculosis and Malaria in Latin America, the total approved HIV grant amount to date has been just over US$85 million [45].

The modelling results presented here are one of the first to quantify the potential population impact of PrEP incorporating data from the only clinical trial carried out in an MSM population. We highlighted the need to represent heterogeneity in adherence behaviour together with a high intrinsic efficacy of PrEP (when adherence is high). It is also to our knowledge the first model to examine the impact among MSM and transwomen in a low- or middle-income country.

Our results are broadly consistent with previous modelling work on the impact of PrEP among MSM in the US, where most authors have found that while PrEP might be cost-effective under certain assumptions, the investment needed remains very high. For instance, Desai et al. [46] found that a PrEP program was cost-effective under most variations in efficacy and adherence—a high impact was possible for an efficacy of 70% and program adherence of 50%. Paltiel et al. [47] also found that PrEP could have a substantial effect on lifetime HIV infection risk, especially among younger populations, and that it could be a cost-saving intervention with drug efficacy levels over 70% and an annual PrEP cost of US$2,500 or less. Recently, Koppenhaver et al. [48] found that although reductions in new HIV cases following PrEP introduction among MSM in New York City led to substantial reductions in treatment costs, these savings were largely offset by increases in PrEP costs. The authors assumed both high costs for PrEP—including costs for implementing PrEP and for tenofovir/emtricitabine (valued at $22/d)—and a coverage of 100% of all susceptible. Juusola et al. [49] varied the coverage of susceptible MSM and also included high costs of PrEP consistent with US costs. Juusola et al. [49] concluded that “PrEP in the general MSM population could prevent a substantial number of HIV infections, but it is expensive”.

Results from modelling studies of PrEP use in generalised epidemics have been conflicting. Abbas et al. [50] found that with high effectiveness, PrEP could have a beneficial impact when targeted to those at highest risk, if it did not lead to an increase in risk behaviours. Recently, Pretorius et al. [39] found a PrEP intervention to be cost-effective according to the WHO-CHOICE threshold, but concluded that it did not provide value for money when compared to the scale-up of ART. Hallett et al. [51] found the use of PrEP in serodiscordant couples to be a cost-effective alternative, especially in couples with increased risk behaviours. Although our findings cannot be directly compared with these, as the settings differ greatly, one explanation for the different results obtained could be the difference in incidence assumptions (higher incidence rates among MSM lead to prevention interventions having a greater value).

Our study balances heterogeneity considerations with data availability and the programmatic relevance of distinguishing particular subgroups, but it has limitations. The model is limited by the need to capture important variation and complexity in sexual behaviour in this population into a simplified framework. To account for this, we applied a Bayesian model calibration procedure. Because of the large amount of consistent prevalence data available for MSM and transwomen in Lima, the best fit was used in our analysis. However, we explored the potential effect of uncertainty around the best fit epidemiological scenario on our main outputs (i.e., the cost-effectiveness of PrEP across all programmatic scenarios) and observed that although the range is broad, it does not change the main conclusions from this analysis. The model does not capture the possibility of evolution of drug resistance generated by individuals using PrEP after breakthrough infection, which has been a concern. However, several models suggest that the amount of resistance generated by individuals using PrEP after breakthrough infection will be small compared to that generated by ART itself [52], provided individuals on PrEP are tested frequently. Our results suggest that only four of every 1,000 PrEP person-years would be wrongly allocated to infected individuals (in a low coverage, high prioritisation scenario) if individuals on PrEP are tested every 3 mo. This increases to eight of every 1,000 PrEP person-years if the delay between tests is set to every 6 mo (results not shown). Moreover, Supervie et al. [53] recently used a mathematical model to explore resistance in the context of PrEP use in the MSM community of San Francisco, California, showing that if risk behaviours did not increase, then transmitted resistance would decline with PrEP.

Furthermore, the validity of DALYs as an aggregate measure of effectiveness depends on assumptions made for disability weighting, discounting, age weighting, and life expectancy. We followed standard practices regarding these assumptions, aiming to increase the comparability of our analysis while providing meaningful results for policy-makers. However, we recognise that standard assumptions about DALYs averted by averting HIV transmission do not fully consider strong preferences to remain HIV-uninfected, given the burden of lifelong daily therapy and stigma associated with HIV infection. In addition, we limited the calculation of the total DALYs averted to a function of the number of infections averted during the 10-y intervention period, assuming 80% of these would have received treatment otherwise. By doing this, we provide a conservative estimate of the cost-effectiveness of PrEP (i.e., not including the savings due to treatment costs averted). However, this could also represent an optimistic approximation of potential benefits, as we assume that those infections averted during the intervention will not happen afterwards. We tested this by running the model for an additional 60 y after the end of the intervention and found that the number of infections averted overall is greater than that observed during the intervention period (results available in Text S1). The spread of infection is a dynamic process; stopping infections during one period protects others from being infected further down the line in a similar way that vaccines do. We did not use this information to produce results, to ensure comparability with other cost-effectiveness analyses using mathematical models of HIV transmission. Additionally, recognising that we made a large number of assumptions to estimate the effect of PrEP during the 10 y of the intervention, we preferred not to extrapolate behavioural assumptions afterwards.

Another limitation of this study is that interactions between PrEP and earlier treatment for prevention of onward transmission are not fully considered. How PrEP and treatment programs will interact in practice remains to be learned. Although capacity for manufacturing ARV drugs, including generic formulations of the most popular agents, has not been limiting, the funding available to purchase these drugs has. Theoretically, PrEP could compete with treatment scale-up by using limited funding or limited clinic capacity, and this should be avoided. Alternatively, PrEP could enable treatment programs, by allowing greater volume discounts on drug prices and costs, by increasing testing coverage, by fostering retention in treatment by destigmatizing ARV drugs and the people who use them, or by fostering popular and political support for attracting more funding to HIV/AIDS initiatives. In the absence of information about how these various possibilities will play out, this paper has aimed to evaluate the potential impact of PrEP as a prevention strategy among a population with relatively high risk of infection and to explore the effect of different programmatic factors such as prioritisation and coverage. While widespread treatment is assumed in this model in the context of universal ART access along current guidelines, a detailed analysis of how PrEP could affect treatment coverage is beyond the scope of available information at this time.

In conclusion, we have shown that if it is prioritised to key groups and has a rapid scale-up, PrEP could be a cost-effective intervention for MSM populations and transwomen in Lima, Peru. Despite cost-effectiveness at apparently feasible levels of coverage and uptake, considerable expenditures and human resources will be required to generate a significant reduction in incidence. These expenditures should not be considered unless well-performing ART services are already in place, which is not the case everywhere in Peru, and even less so across the region. If such conditions are assured, however, a strategically implemented PrEP programme could make a significant contribution as part of a combination package of priority, well-implemented interventions for MSM/transwomen populations in concentrated epidemics after a scale-up of ART.

## Supporting Information

Alternative Language Text S1 **Spanish translation of the article.**
(DOC)

Figure S1 **Estimated impact of PrEP with respect to coverage, adherence, and prioritisation of key populations: number of infections averted/1,000 PrEP person-years.** Impact is shown as infections averted for every 1,000 PrEP person-years (PY). The error bars reflect the uncertainty in the iPrEx efficacy estimate of 92% (95% CI 40–99). In this comparison, we show two scenarios ([A]: low coverage, in green; [B]: high coverage, in blue) for three adherence profiles including three prioritisation strategies—uniform, where the coverage is the same in each subgroup; some prioritisation, where there is higher coverage achieved in the transwomen at higher risk and sex worker populations (but no more than 50% covered) than in MMSW and MMSM; and high prioritisation, where 90% of transwomen at higher risk and 11% of sex workers receive PrEP in the low coverage scenario, or 90% of transwomen at higher risk, 90% of sex workers, 3.9% of MMSM, and 21.5% of MMSW receive PrEP in the high coverage scenario.
(TIFF)

Figure S2 **Estimated impact of PrEP with respect to coverage, adherence, and prioritisation of key populations: proportion of infections averted over 10 y.** Impact is shown as percentage of total infections that are averted with PrEP. The error bars reflect the uncertainty in the iPrEx efficacy estimate of 92% (95% CI 40–99). In this comparison, we show two scenarios ([A]: low coverage, in green; [B]: high coverage, in blue) for three adherence profiles including three prioritisation strategies—uniform, where the coverage is the same in each subgroup; some prioritisation, where there is higher coverage achieved in the transwomen at higher risk and sex worker populations (but no more than 50% covered) than in MMSW and MMSM; and high prioritisation, where 90% of transwomen at higher risk and 11% of sex workers receive PrEP in the low coverage scenario, or 90% of transwomen at higher risk, 90% of sex workers, 3.9% of MMSM, and 21.5% of MMSW receive PrEP in the high coverage scenario.
(TIFF)

Figure S3 **Cost-effectiveness of PrEP, estimated as cost per DALY averted: downstream ARV costs included.** (A) Downstream ARV costs averted included at US$1,000/person-year on ARV drugs. (B) Downstream ARV costs averted included at US$3,500/person-year on ARV drugs. iPrEx adherence profile used for these scenarios. In green: low coverage scenario: 5%; in blue: high coverage scenario: 20%. The variation in costs reflects the uncertainty in the costing of one person-year on PrEP. The red lines correspond to (1) the World Bank threshold for a cost-effective intervention, <US$745/DALY averted, and (2) the World Bank threshold for a highly cost-effective intervention, <US$149/DALY averted.
(TIFF)

Figure S4 **Cost-effectiveness and total cost of PrEP over 10 y: downstream ARV costs not included.** iPrEx adherence profile used for all scenarios. In green: low coverage scenario: 5%; in blue: high coverage scenario: 20%. Lines are plotted against the *y*-axis of cost/DALY averted in US dollars and against the *x*-axis—total number of DALYs averted over 10 y. The numbers over the lines indicate the data points as follows: 1, low coverage, uniform scenario; 2, low coverage, some prioritisation scenario; 3, low coverage, high prioritisation scenario; 4, high coverage, uniform scenario; 5, high coverage, some prioritisation scenario; 6, high coverage, high prioritisation scenario. These data points have uncertainty bars representing the variation in the costing of one person-year on PrEP. The boxes are plotted against the right-hand axis only. They represent the total cost of scenarios. The variation in costs (height of boxes) reflects the uncertainty in the costing of one person-year on PrEP.
(TIFF)

Figure S5 **Cost-effectiveness and total cost of PrEP over 10 y: downstream ARV costs averted included at US$1,000/person-year on ARV drugs.** iPrEx adherence profile used for all scenarios. In green: low coverage scenario: 5%; in blue: high coverage scenario: 20%. Lines are plotted against the *y*-axis of cost/DALY averted in US dollars and against the *x*-axis—total number of DALYs averted over 10 y. The numbers over the lines indicate the data points as follows: 1, low coverage, uniform scenario; 2, low coverage, some prioritisation scenario; 3, low coverage, high prioritisation scenario; 4, high coverage, uniform scenario; 5, high coverage, some prioritisation scenario; 6, high coverage, high prioritisation scenario. These data points have uncertainty bars representing the variation in the costing of one person-year on PrEP. The boxes are plotted against the righthand axis only. They represent the total cost of scenarios. The variation in costs (height of boxes) reflects the uncertainty in the costing of one person-year on PrEP.
(TIFF)

Figure S6 **Cost-effectiveness and total cost of PrEP over 10 y: downstream ARV costs averted included at US$3,500/person-year on ARV drugs.** iPrEx adherence profile used for all scenarios. In green: low coverage scenario: 5%; in blue: high coverage scenario: 20%. Lines are plotted against the *y*-axis of cost/DALY averted in US dollars and against the *x*-axis—total number of DALYs averted over 10 y. The numbers over the lines indicate the data points as follows: 1, low coverage, uniform scenario; 2, low coverage, some prioritisation scenario; 3, low coverage, high prioritisation scenario; 4, high coverage, uniform scenario; 5, high coverage, some prioritisation scenario; 6, high coverage, high prioritisation scenario. These data points have uncertainty bars representing the variation in the costing of one person-year on PrEP. The boxes are plotted against the righthand axis only. They represent the total cost of scenarios. The variation in costs (height of boxes) reflects the uncertainty in the costing of one person-year on PrEP.
(TIFF)

Figure S7 **Cost of PrEP with respect to changes in condom use for a high prioritisation strategy.** (A) Downstream ARV costs averted included at US$1,000/person-year on ARV drugs. (B) Downstream ARV costs averted included at US$3,500/person-year on ARV drugs. This figure assumes there is no correlation between adherence and risk compensation. We explore this issue separately in Figure S12. iPrEx adherence profile used for these scenarios. In green: low coverage scenario: 5%; in blue: high coverage scenario: 20%. The red lines correspond to (1) the World Bank threshold for a cost-effective intervention, <US$745/DALY averted, and (2) the World Bank threshold for a highly cost-effective intervention, <US$149/DALY averted.
(TIFF)

Figure S8 **Population impact of PrEP with respect to changes in condom use.** (A) “Uniform” scenario. (B) “Some prioritisation” scenario. This figure assumes there is no correlation between adherence and risk compensation. We explore this issue separately in Figure S12. In green: low coverage scenario: 5%; in blue: high coverage scenario: 20%. Reference is no change in condom use.
(TIFF)

Figure S9 **Cost-effectiveness of PrEP with respect to changes in condom use: downstream ARV costs averted not included.** (A) “Uniform” scenario. (B) “Some prioritisation” scenario. This figure assumes there is no correlation between adherence and risk compensation. We explore this issue separately in Figure S12. iPrEx adherence profile used for these scenarios. In green: low coverage scenario: 5%; in blue: high coverage scenario: 20%. The red lines correspond to (1) the World Bank threshold for a cost-effective intervention, <US$745/DALY averted, and (2) the World Bank threshold for a highly cost-effective intervention, <US$149/DALY averted.
(TIFF)

Figure S10 **Cost-effectiveness of PrEP with respect to changes in condom use: downstream ARV costs averted included at US$1,000/person-years on ARV drugs.** (A) “Uniform” scenario. (B) “Some prioritisation” scenario. This figure assumes there is no correlation between adherence and risk compensation. We explore this issue separately in Figure S12. iPrEx adherence profile used for these scenarios. In green: low coverage scenario: 5%; in blue: high coverage scenario: 20%. The red lines correspond to (1) the World Bank threshold for a cost-effective intervention, <US$745/DALY averted, and (2) the World Bank threshold for a highly cost-effective intervention, <US$149/DALY averted.
(TIFF)

Figure S11 **Cost-effectiveness of PrEP with respect to changes in condom use: downstream ARV costs averted included at US$3,500/person-year on ARV drugs.** (A) “Uniform” scenario. (B) “Some prioritisation” scenario. This figure assumes there is no correlation between adherence and risk compensation. We explore this issue separately in Figure S12. iPrEx adherence profile used for these scenarios. In green: low coverage scenario: 5%; in blue: high coverage scenario: 20%. The red lines correspond to (1) the World Bank threshold for a cost-effective intervention, <US$745/DALY averted, and (2) the World Bank threshold for a highly cost-effective intervention, <US$149/DALY averted.
(TIFF)

Figure S12 **Population impact of PrEP with respect to differential changes in condom use.** (A) Only good adherers change their behaviour. (B) Only bad adherers change their behaviour. This figure assumes there is a correlation between adherence and risk compensation. iPrEx adherence profile used for these scenarios. In green: low coverage scenario: 5%; in blue: high coverage scenario: 20%. Reference is no change in condom use.
(TIFF)

Table S1 **Scenarios to achieve one-third of new infections averted over 10 y: downstream ARV costs averted included at US$1,000 and 3,500/person-year on ARV drugs.** Total PY: total number of person-years on PrEP over 10 y. Cost/DALY: cost in US dollars/DALY. Total cost: total cost of PrEP intervention over 10 y. (1) Includes downstream ARV costs averted included at US$1,000/person-year on ARV drugs. (2) Includes downstream ARV costs averted included at US$3,500/person-year on ARV drugs. The range observed in this column and in the Total cost column represents the variation observed in the costs per PrEP person-year. Coverage in the uniform distribution strategy is equal in all subgroups (MMSW, MMSM, sex workers, and transwomen at higher risk). The coverage showed in both strategies involving prioritisation (some and high) are given as overall population coverage and, in brackets, the coverage in each subpopulation (MMSW/MMSM/sex workers/transwomen at higher risk).
(DOC)

Text S1 **Supplementary technical information.**
(PDF)

## Abbreviations

ART: antiretroviral treatment
ARV: antiretroviral
DALY: disability-adjusted life year
GDP: gross domestic product
iPrEx: Pre-Exposure Prophylaxis Initiative
MMSM: men who mostly have sex with men
MMSW: men who mostly have sex with women
MSM: men who have sex with men
PrEP: HIV pre-exposure prophylaxis
WHO-CHOICE: World Health Organization Choosing Interventions That Are Cost-Effective
