# Supplementary material for: The Potential Impact of Pre-Exposure Prophylaxis for HIV Prevention among Men Who Have Sex with Men and Transwomen in Lima, Peru: A Mathematical Modelling Study
Source: PLoS Med. 2012 Oct 9;9(10):e1001323. doi: 10.1371/journal.pmed.1001323 (PMC3467261; doi:10.1371/journal.pmed.1001323)
Supplement: Alternative Language Text S1 — Spanish translation of the article. (DOC) [file pmed.1001323.s001.doc]

**El impacto potencial de la profilaxis pre-exposición para la prevención del VIH entre hombres que tienen sexo con hombres y mujeres trans en Lima, Perú: un estudio de modelamiento matemático.**

Gabriela B Gomez, PhD^1,2*^; Annick Borquez, MSc^1*†^; Carlos F Caceres, PhD^3,4^; Eddy R Segura, MD^4^; Robert M Grant, MD^5^; Geoff P Garnett, PhD^1^; Timothy B Hallett, PhD^1^

*Contribuyeron equitativamente

^1^Department of Infectious Disease Epidemiology, School of Public Health, Imperial College London, UK;

^2^Amsterdam Institute for Global Health and Development, Amsterdam, The Netherlands;

^3^Instituto de Estudios en Salud, Sexualidad y Desarrollo Humano, Lima, Peru;

^4^Universidad Peruana Cayetano Heredia, Lima, Peru;

^5^Gladstone Institutes, University of California at San Francisco, San Francisco, USA

^†^Persona de contacto

Department of Infectious Disease Epidemiology, Faculty of Medicine, Imperial College London, St Mary's Campus, Norfolk Place, London W2 1PG, United Kingdom

Tel: +44 (0)20 7594 3290 Fax: +44 (0)20 7594 8321

[annick.borquez06@imperial.ac.uk](mailto:annick.borquez06@imperial.ac.uk)

Recuento de palabras (excluyendo el resumen, tablas, notas, figuras, leyendas, reconocimientos, conflicto de interés, financiamiento y referencias): 6,859 palabras

Palabras clave – profilaxis pre-exposición para el VIH, hombres que tienen sexo con hombres, modelamiento matemático, costo-efectividad, prevención combinada.

**Resumen**

Antecedentes

La profilaxis pre-exposición (PrEP) para el VIH, el uso de medicamentos antirretrovirales por individuos no infectados para prevenir la infección por VIH, demostró efectividad al prevenir la adquisición en una población de alto riesgo de hombres que tienen sexo con hombres (HSH). En consecuencia, existe una necesidad de entender si y cómo la PrEP puede ser usada de manera costo-efectiva para prevenir la infección por VIH en las poblaciones.

Métodos y Hallazgos

Nosotros desarrollamos un modelo matemático que representa la epidemia del VIH entre HSH y mujeres trans en Lima, Perú como un caso de prueba. Se asume que la efectividad de la PrEP en el modelo resulta de la combinación de un parámetro de “eficacia condicional” y un parámetro de adherencia. Los costos operativos anuales desde la perspectiva de un profesional de la salud se basaron en las pautas interinas de los CDC para el uso de PrEP. El modelo fue usado para investigar el impacto al nivel de la población, el costo y el costo-efectividad de la PrEP bajo una variedad de escenarios de implementación.

El impacto epidemiológico de la PrEP es ampliamente guiado por las características del programa. Para una modesta cobertura PrEP del 5%, más del 8% de las infecciones pudieron ser evitadas en un programa que da prioridad a aquellos con más alto riesgo y logrando niveles de adherencia de iPrEX. A través de todos los escenarios, el costo estimado más alto por AVAD evitado (estrategia uniforme para un nivel de cobertura del 20%, US$ 1,036 a 4,254) se encuentra por debajo del umbral recomendado por la OMS para las intervenciones costo-efectivas; mientras que es probable que sólo ciertos escenarios optimistas (baja cobertura del 5% y un poco o alta prioridad) sean costo-efectivos usando el umbral del Banco Mundial. El impacto de la PrEP es reducido si aquellos en PrEP disminuyen el uso del condón pero solo cambios extremos en el comportamiento entre no cumplidores (más del 80% de reducción en el uso de condón) y una eficacia condicional PrEP baja (40%) impactaría negativamente la epidemia. Sin embargo, la PrEP no detendrá la transmisión del VIH de forma aislada debido a su efectividad incompleta, dependencia a la adherencia y el alto costo de los programas limitando niveles de cobertura alcanzables.

Interpretación

Una intervención estratégica de PrEP podría ser una adición costo-efectiva a las estrategias existentes de prevención del VIH para las poblaciones HSH. Sin embargo, a pesar de ser costo-efectiva, se requeriría un gasto sustancial para generar reducciones significativas en la incidencia.

**Introducción**

El uso de medicamentos anti-retrovirales para la prevención de la adquisición o transmisión del VIH es actualmente un foco de discusiones políticas. Su uso en individuos no infectados por el VIH para prevenir la adquisición del VIH – profilaxis pre-exposición (PrEP) – es una de las alternativas que están siendo consideradas como una herramienta potencial en el arsenal de prevención del VIH[1]. En el 2010, los resultados del primer ensayo clínico de fase III de PrEP fueron publicados: La Iniciativa Profilaxis Pre-Exposición (iPrEX) fue un ensayo multinacional de tenofovir/emtricitabina oral diario para prevenir la adquisición del VIH entre hombres que tienen sexo con hombres (HSH) del alto riesgo[2]. Este mostró que el régimen era seguro y reducía el riesgo de adquisición del VIH en un 44%[2]. Por consiguiente, la Organización Mundial de la Salud, el Centro para el Control y la Prevención de Enfermedades (EE.UU.), la Asociación Británica para la Salud Sexual y el VIH y la sociedad de Médicos Clínicos del VIH de Sudáfrica han publicado guías interinas acerca de la PrEP[3, 4, 5, 6], recomendando su uso como parte de un programa de prevención integral del VIH. Recientemente, la Administración de Alimentos y Medicamentos de EE.UU. aprobó el uso de medicamentos antirretrovirales (tenofovir/emtricitabina, nombre comercial Truvada) como profilaxis pre-exposición entre hombres y mujeres[7]. Se están llevando a cabo las consultas para informar a los formuladores de políticas de salud pública en el desarrollo de pautas clínicas y de servicio con respecto a la PrEP. Adicionalmente, añadiendo impulso a este campo de rápido movimiento, en algunos estudios también se halló que la PrEP es efectiva en la prevención de adquisición del VIH entre hombres y mujeres heterosexuales en el África subsahariana[8, 9]. Sin embargo, FEM-PrEP, un ensayo que reclutaba mujeres heterosexuales en Sudáfrica, Tanzania y Kenia fue cerrado prematuramente el año pasado cuando el comité de revisión de la data declaró que no sería capaz de demostrar un efecto de la PrEP[10]. Dos ensayos adicionales han probado la eficacia del gel de tenofovir al 1% con resultados un tanto inconsistentes. El CAPRISA 004 halló una reducción en el riesgo de las mujeres en un 39%[11]; mientras que el brazo del gel en el ensayo VOICE fue detenido tempranamente luego de determinar que el producto era seguro pero no efectivo[12]. Existe, por lo tanto, una necesidad de entender si y cómo la PrEP podría prevenir de manera costo-efectiva la infección por VIH en poblaciones dentro del contexto actual de expansión del acceso al tratamiento.

Aunque los ensayos pueden demostrar efectividad al reducir la probabilidad de que un individuo adquiera el VIH, no muestran la medida en que la intervención de la PrEP reduciría la propagación del VIH al nivel de la población. Aún quedan preguntas acerca de cómo implementar óptimamente la PrEP cuando son posibles múltiples formas de entrega, priorizaciones y ampliación en el contexto de otras intervenciones para la prevención del VIH. En respuesta, hemos construido y analizado un modelo matemático de la epidemia del VIH entre HSH y mujeres trans en Lima, Perú. Los modelos matemáticos proporcionan un marco para examinar el impacto potencial de las intervenciones que podrían dar información para el desarrollo de políticas[13]. La epidemia del VIH en Lima, Perú se concentra entre los HSH, comparable a la mayor parte de América Latina y múltiples entornos de altos ingresos[14]. Como tal, proporciona un “caso de prueba” para el cual existe información de comportamiento y epidemiológica de alta calidad para especificar adecuadamente un modelo matemático. Por otra parte, la mayoría de los participantes de la iPrEX fueron reclutados en Perú, permitiéndonos incluir información representativa acerca del uso potencial de la PrEP. Nuestro análisis tiene como objetivo proporcionar información para asistir en el proceso de traducir los resultados de ensayos recientes en programas costo-efectivos.

**Métodos**

En este documento presentamos los beneficios y costos potenciales de una intervención hipotética de PrEP usando un modelo compartimental determinista para representar la transmisión sexual del VIH entre HSH y mujeres trans en Lima, Perú. Nuestro objetivo era investigar el impacto de una intervención viable y determinar las estrategias más eficientes para su despliegue en esta población. En concreto, vimos el impacto de la cobertura, adherencia y priorización tanto en los beneficios de salud como en los costos para el sistema de salud.

Lima tiene una población HSH diversa que definimos como hombres y mujeres trans que han reportado una relación sexual con un hombre en los últimos 12 meses. Para representar la propagación del VIH en el modelo, definimos cuatro grupos que interactúan: hombres que en su mayoría tienen sexo con mujeres (HMSM), hombres que en su mayoría tienen sexo con hombres (HMSH), trabajadores sexuales, y mujeres trans (incluyendo transexuales y travestis) en mayor riesgo. Estas categorías intentan representar un amplio espectro de las identidades, orientaciones y comportamientos sexuales, incluyendo números de parejas, tipos de relación formada (estable, casual, comercial), uso del condón y trabajo sexual (definido como el intercambio de sexo anal por dinero, drogas, regalos o favores). Nuestras definiciones de subgrupos siguieron la clasificación comúnmente utilizada en estudios de investigación en Perú[14]. Algunos grupos fueron nuevamente subdivididos en compartimentos mutuamente exclusivos de acuerdo con su posición sexual durante el sexo anal (insertivo, receptivo o versátil, es decir, practica tanto el sexo anal insertivo como el receptivo) resultando en un total de nueve grupos (Figura 1. Más información en las definiciones de grupos puede ser ubicada en el Texto S1). Para estimar la distribución de los HSH en los diferentes grupos de riesgo, revisamos estudios que describían la proporción de HSH que reportaron sexo con una mujer en el último año y/o la proporción de HSH que se auto identificaban como heterosexuales o bisexuales (para HMSM), como homosexuales/gay (para HMSH) y como transgénero, travestis o transexuales. Para estimar la proporción de trabajadores sexuales, sustrajimos las proporciones de HMSM, HMSH y mujeres trans del total. Luego comparamos este estimado contra las proporciones reportadas de HSH que se estaban involucrando en sexo comercial durante el último año[15, 16, 17, 18, 19, 20]. La frecuencia de formación de parejas se basó en el número reportado de parejas sexuales de estudios publicados y data no publicada de estudios de mujeres trans (estudio Trans-Amfar)[21] y HSH (estudio CPOS)[22]. Las relaciones estables, casuales y comerciales se definen por frecuencias de actos sexuales. Para el grupo versátil, el número total de actos sexuales fue dividido en una proporción de actos insertivos o receptivos. El uso del condón fue estimado de la data de uso reportado de condón durante el último acto sexual para los diferentes tipos de relación. Si la información estaba disponible tanto para las parejas insertivas como receptivas, la probabilidad de usar un condón estaba determinada por la pareja receptiva. Las parejas receptivas tienen una probabilidad más elevada de infección que las parejas insertivas y el uso del condón tendrá un mayor impacto en la pareja receptiva. La prevalencia del VIH en Lima, en la población en general, en baja y por lo tanto el riesgo de transmisión a HSH y personas transgénero desde otras fuentes no fue incluido (las tablas que detallan los patrones de contacto por tipo de relación, prevalencia de VIH por grupo, distribuciones de grupo y parámetros demográficos y de comportamiento usados en el modelo pueden ser encontradas en el Texto S1).

**Figura 1:** Representación del modelo de mezcla sexual y posicionamiento sexual entre HSH y mujeres trans

Basado en los trabajos previamente publicados, el curso natural de la infección por VIH fue representado como cuatro fases de la progresión de la enfermedad definidas por duración y infecciosidad: infección aguda, fase latente, pre-SIDA y SIDA (ver Texto S1)[23, 24, 25, 26]. Una proporción de individuos inicia tratamiento antirretroviral (TAR) en lugar de progresar a pre-SIDA, dependiendo del nivel de cobertura. Se asume que el TAR reduce la infecciosidad y extiende la supervivencia[27].

Tres fuentes de información fueron utilizadas para calibrar el modelo: comportamiento reportado, historia natural del VIH y data sobre la prevalencia e incidencia para sub-poblaciones de HSH y mujeres trans en Lima (del Ministerio de Salud Peruano y estudios publicados. Estas fuentes y valores se encuentran detalladas en el Texto S1). Utilizamos un procedimiento cuasi Bayesiano para combinar estas fuentes de información y justificar la incertidumbre en torno a las estimaciones de los parámetros [28,29]. Este procedimiento consiste en permitir que ciertos parámetros varíen dentro de una distribución previa especificada reflejando su incertidumbre. Los parámetros a los que se les permite variar describen la distribución del riesgo en la población y comportamientos de riesgo (presentados en el Texto S1). Estos son específicos para cada entorno y son más vulnerables al sesgo en el método de muestreo y al sesgo de reporte. El muestreo de los valores de parámetros fue llevado a cabo usando el muestreo por hipercubo latino[30]. Cada modelo corrido es el resultado de una combinación diferente de valores de parámetros. Los límites previos sobre la prevalencia son definidos con el fin de seleccionar corridas plausibles y luego se determina el mejor conjunto de ajuste de parámetros usando el logaritmo de la verosimilitud (los detalles sobre límites previos elegidos se dan en el Texto S1). De 10,000 corridas realizadas, 449 fueron seleccionadas y son mostradas en la Figura 2, junto con el mejor ajuste en base a la data de prevalencia para cuatro sub-grupos y la población en general. Este mejor ajuste fue usado luego para los análisis principales. Para asegurar que fuera representativo del escenario epidemiológico más probable exploramos la incertidumbre debido a los supuestos epidemiológicos y comparamos los resultados obtenidos usando el conjunto de parámetros correspondiente al mejor ajuste con aquellos de todas las otras corridas seleccionadas en el proceso Bayesiano. La distribución de infecciones evitadas obtenida de todas las corridas seleccionadas y los 50 mejores ajustes son presentados en el Texto S1 y comparados con los estimados obtenidos del mejor ajuste para los escenarios más relevantes.

**Figura 2:** Corridas seleccionadas del modelo y mejor ajuste en base a la data de prevalencia para cuatro sub-grupos y la población en general.

Supuestos acerca de la intervención de profilaxis pre-exposición en el modelo

Se asume que la efectividad de la PrEP en el modelo resulta de la combinación de una reducción en la susceptibilidad a la infección por VIH durante un acto sexual protegido mediante PrEP (parámetro de “eficacia condicional”) y una proporción de actos sexuales protegidos (parámetro de adherencia). Nuestro objetivo era reflejar un régimen diario de PrEP y un equivalente de eficacia general al ensayo iPrEX, 44%[95%IC 15-63][2]. En un análisis retrospectivo de la data del ensayo iPrEX, para aquellos con niveles detectables del medicamento en las muestras de sangre, la incidencia del VIH se redujo en un 92%[95%UC 40-99]. Interpretamos que estos hallazgos muestran una eficacia de PrEP que varía con el nivel de adherencia individual, en concordancia con los resultados presentados recientemente por los cuales los determinantes biológicos no explicaban la eficacia diferencial observada en el ensayo iPrEX[31]. Nos referimos a este parámetro como una “eficacia condicional” en el modelo para reflejar un supuesto de alta eficacia intrínseca de la PrEP (equivalente al 92%) pero una amplia heterogeneidad a nivel población en los comportamientos de adherencia. Por lo tanto, dividimos a los usuarios de PrEP en tres grupos de adherencia usando estimaciones puntuales: buena (95%), promedio (45%) y pobre (15%). La proporción en cada grupo fue alterada para reflejar tres perfiles de adherencia: el perfil de “adherencia iPrEX”, para el cual las proporciones fueron de acuerdo a las observadas en el ensayo y los escenarios “menos adherencia” y “más adherencia”. El escenario que reproduce el comportamiento de “más adherencia” corresponde a la hipótesis que está siendo probada en la extensión de etiqueta abierta del ensayo iPrEX viendo un posible incremento en el uso del medicamento de estudio por los participantes, debido al conocimiento de los participantes de recibir un medicamento activo que proporciona alguna protección contra la infección del VIH. Para todos los escenarios, cuando ambos, la “eficacia condicional” y los perfiles de adherencia, se combinan, la efectividad funcional calculada reproduce la efectividad observada en el ensayo iPrEX, 44%[95%IC 15-63][2]. La Tabla 1 muestra los supuestos realizados para la “eficacia condicional” y la adherencia así como también la efectividad funcional resultante para cada perfil.

Tabla 1 – Definición de escenario: Impacto de la PrEP por adherencia y efectividad funcional

Construimos dos escenarios para la ampliación general de los programas de PrEP: (i) “baja cobertura” y (ii) “alta cobertura”, en donde el 5% y 20% de los individuos no infectados usan PrEP respectivamente. En ambos escenarios, la ampliación comienza en el 2012, la cobertura se alcanza en cinco años y se mantiene a partir de entonces por cinco años más. Cuantificamos la contribución de cada sub-población al número de futuras infecciones por VIH y el impacto relativo de la PrEP de usarse en cada grupo por separado para identificar puntos clave para una intervención. Vimos el impacto de distribuir una cantidad fija de PrEP (25,000 años-persona, correspondiente al equivalente de PrEP del 10% de la asignación de recursos del programa nacional de prevención[32]) a cada grupo por separado. Entonces la PrEP podría ser distribuida equitativamente a todos los HSH y mujeres trans (cobertura “uniforme”) o ciertos grupos clave podrían ser priorizados para la asignación de PrEP (“priorización”). Los escenarios priorizados podrían resultar en “poca priorización”, donde una mayor cobertura es lograda en poblaciones clave, tales como los grupos de mujeres trans y trabajadores sexuales (pero no más del 50% de cobertura) comparado con los HMSM y HMSH; y en un escenario de “alta priorización”, una vez que el 90% del grupo transgénero recibe la PrEP, el monto residual (para lograr el 5% o 20% de la cobertura total) es dividido entre las otras tres poblaciones priorizando a los trabajadores sexuales sobre los HMSM y HMSH.

Tabla 2 – Definición de escenario: Impacto de la PrEP por cobertura y priorización

Cálculos de costo-efectividad

Estimamos el costo operativo anual de proporcionar una hipotética intervención de PrEP a individuos, desde una perspectiva del profesional de la salud, en base a las pautas interinas del CDC para PrEP[3]. De acuerdo con estas recomendaciones clínicas, incluimos: pruebas de VIH antes de iniciar la PrEP, pruebas de VIH cada tres meses durante el uso de la PrEP, pruebas confirmatorias de VIH en un individuo si resultara positivo, una prueba de creatinina/NUS por año durante el uso de PrEP, servicios de alcance comunitario y consejería, promoción y provisión de condones y lubricantes. La estimación incluye costos adicionales para recursos humanos pero no incluye costos relacionados con el tratamiento del VIH luego de la infección, pruebas de resistencia o pruebas y tratamiento de otras infecciones transmitidas sexualmente. Incluimos un 5% extra de costos para permitir la creación de un ambiente de apoyo a nivel de proyecto, costos de manejo del programa o costos de monitoreo y evaluación a nivel de proyecto[33]. Este margen del 5% fue elegido en base al presupuesto reportado para el manejo, monitoreo y evaluación de programas de VIH a nivel nacional[32]. No incluimos costos indirectos tales como ingresos potenciales no percibidos por pacientes o cuidadores. Presentamos todos los costos a precios de mercado sin ajustar, específicos para Perú[32, 34, 35] y calculados para diez años (es decir, la duración de nuestra intervención simulada).

Estimamos que el costo unitario de una intervención de PrEP se encuentra entre los US$525 y US$830, de los cuales el componente principal era el costo de los medicamentos PrEP (por encima de los dos tercios del total estimado). Se estableció que el costo de los medicamentos utilizados para la PrEP se encontraba entre los US$ 420 y US$ 600 por año en base a una data de costos proporcionada por Gilead (Viread® US$ 30 y Truvada® US$ 45, más 10 a 15% de margen del distribuidor por botella, un costo de botella por mes)[34].

Para el análisis de costo-efectividad, nuestro principal resultado epidemiológico fue costo por AVAD (años de vida ajustados por discapacidad) evitados. Los escenarios de implementación de la PrEP fueron evaluados contra un escenario de “sin intervención de PrEP”. El número total de AVADs evitados fueron entonces calculados usando el número de infecciones evitadas en cada escenario. La incertidumbre debido a los supuestos epidemiológicos fue representada al agregar intervalos de credibilidad en nuestro análisis principal. Estos intervalos de credibilidad corresponden a las corridas del modelo estimando el máximo y mínimo impacto de la PrEP en términos de infecciones evitadas por año persona en PrEP. El número estimado de AVADs asociado con una infección por VIH evitada se calculó como la suma de los números de años de vida perdidos (AVPs) y el número de años perdidos debido a discapacidad (APDs) usando métodos publicados[35, 36]. Estos cálculos incluyeron pesos para la esperanza de vida peruana, edad, tiempo futuro y discapacidad. La duración de discapacidad fue calculada en base a la data clínica local para pacientes VIH positivos que se presentan para recibir atención[37] y los pesos de discapacidad para condiciones relacionadas con el VIH fueron obtenidos del estudio Carga Mundial de Enfermedad[38]. Estimamos aproximadamente 12.3 AVADs evitados descontados por infección evitada, incluyendo acceso a TAR para el 80% de individuos infectados. Cuando excluimos la función de ponderación de la edad, estimamos 11.5 AVADs evitados descontados. Esto es el equivalente a 27.09 (y 27.12 cuando excluimos la función de ponderación de la edad) AVADs evitados por infección evitada si no se asume ningún descuento, comparable a otros estimados en la literatura sobre el VIH[39, 40]. Todos los detalles de los cálculos de costos y estimados de AVAD se encuentran en el Texto S1. Costos, ahorros y ganancias de salud futuros fueron descontados a una tasa del 3%. Incluimos como material suplementario un análisis de sensibilidad de todos nuestros resultados si costos posteriores de tratamiento evitados eran incluidos en la evaluación. El costo del tratamiento antirretroviral para este análisis varía desde US$ 1,000[41] a US$ 3,500[39].

Actualmente, existe una selección subjetiva de umbrales de costo-efectividad en la literatura[42]. Hacemos referencia a dos umbrales comúnmente usados. La iniciativa Selección de Intervenciones que son Costo-Efectivas de la OMS (WHO-CHOICE) considera que una intervención es: 1) muy costo-efectiva si su costo es menor que el producto bruto interno (PBI) per cápita (<US$ 5,401) por AVAD evitado; 2) costo-efectiva para un costo entre uno y tres veces el PBI per cápita (US$ 5,401 a US$ 16,203) por AVAD evitado; y 3) no costo-efectiva si cuesta más de tres veces el PBI per cápita (>US$ 16,203) por AVAD evitado. Los valores de PBI son aquellos estimados para Perú en el 2010[35, 43]. El segundo umbral mostrado es un punto de corte más conservador sugerido por el Banco Mundial en 1993 para países de ingreso medio[44]: <US$ 100 por AVAD evitado para reflejar una intervención altamente costo-efectiva, entre US$ 100 a US$ 500 para una intervención costo-efectiva y >US$ 500 para que una intervención sea considerada no costo-efectiva. Estos puntos de corte fueron considerados en el análisis ajustados por inflación al 2010 – US$ 149 y US$ 745 respectivamente.

Análisis

Cuantificamos el impacto, costo y costo efectividad de diferentes patrones de uso de PrEP a través de la población, identificando dependencias en la eficacia condicional de PrEP, cobertura, periodo de ampliación, priorización de grupos clave y adherencia. El impacto de compensación del riesgo donde aquellos en PrEP redujeron el uso del condón fue investigado. Luego usamos el modelo para encontrar escenarios de intervención que resultarían en la reducción del número de nuevas infecciones en un tercio, lo que representa un objetivo de planificación relevante para Lima.

**Resultados**

1. *Grupos clave para priorizar entre HSH y personas transgénero*

Para establecer un conjunto apropiado de estrategias para usar PrEP, es importante identificar qué grupos priorizar. Los individuos que podrían ser una prioridad se pudieron determinar por (i) el número de personas similares y la facilidad de llegar a ellos, (ii) su propio riesgo de VIH y (iii) su contribución relativa a la posterior transmisión. En Lima, la mayoría de HSH son hombres que mayormente tienen sexo con hombres (aprox. 70%) y hombres que mayormente tienen sexo con mujeres (aprox. 15%), pero ambos grupos experimentan un riesgo relativamente bajo de infección (2.5 y 1.0% de incidencia modelada en el 2010, respectivamente). Las mujeres trans (aprox. 5%) y trabajadores sexuales (aprox. 10%) son grupos más pequeños, pero con un mayor riesgo de infección (7.3 y 3.1% de incidencia modelada en el 2010, respectivamente). Un monto fijo de PrEP (25,000 años-persona) permitiría cubrir el 60% de mujeres trans, 20% de trabajadores sexuales, 3% de HMSH y 16% de HMSM en diez años. Usado por el sub-grupo de mujeres trans o trabajadores sexuales, se esperaría que 25,000 años-persona de PrEP evitaran 4.7% o 3.4% de infecciones en un lapso de diez años en la población total respectivamente; mientras que la misma cantidad de PrEP usada en otros grupos evitaría menos infecciones – 0.9%, 1.2% para HMSM y HMSH (Figura 3). Por estas razones, en este contexto epidémico, se podría esperar que las estrategias que priorizan a las mujeres trans en mayor riesgo y trabajadores sexuales tengan un mayor impacto por el mismo costo, incluyendo entre aquellos que no están tomando PrEP debido a la prevención de una infección posterior.

**Figura 3.** Proporción de nuevas infecciones evitadas totales en diez años entregando 25,000 PrEP años-persona para cada grupo por separado.

1. *Potencial impacto de programas de PrEP como una función de adherencia, cobertura y estrategias de priorización*

Estimamos el impacto de una intervención de PrEP en diez años bajo una variedad de estrategias de implementación. La Figura 4 muestra el número de infecciones evitadas en diez años (número de infecciones evitadas por 1,000 años-persona en PrEP y la proporción de infecciones evitadas en diez años se muestran en material suplementario). Para un programa modesto de 5% de cobertura de PrEP, más del 8% de infecciones pudo ser evitado con un nivel de adherencia equivalente al observado en el ensayo iPrEX y la priorización (escenario de alta priorización) de los grupos con mayor probabilidad de infectarse. El priorizar la PrEP a trabajadores sexuales y mujeres trans en lugar de lograr una cobertura uniforme incrementa el impacto de las intervenciones. Este es particularmente el caso cuando la cobertura total es baja. Por ejemplo, para un perfil de adherencia de iPrEX, con una cobertura del 5%, el número de infecciones evitadas con una estrategia de cobertura uniforme (970[394-1,060]) se incrementa en más del doble al priorizar la PrEP en un escenario de alta priorización (2,519[1,086-2,713]). El incremento de la adherencia individual a la PrEP incrementa su impacto estimado a nivel de la población. Con niveles más altos de cobertura, el impacto de las intervenciones de PrEP es mayor, pero el beneficio relativo se reduce (los números de infecciones evitadas por año de PrEP disminuyen). Esto se debe a que, en el escenario de alta cobertura, los años-persona de PrEP se vuelven de manera relativa menos eficientemente asignados a los individuos con bajo riesgo.

**Figura 4.** Impacto estimado de PrEP con respecto a la cobertura, adherencia y priorización de poblaciones clave.

1. *Costo-Efectividad de intervenciones PrEP*

El costo por AVAD evitado fue cuantificado para las intervenciones PrEP asumiendo el perfil de adherencia de iPrEX (Figura 5A). A través de todos los escenarios, el costo estimado más elevado por AVAD evitado (una estrategia uniforme para un nivel de cobertura del 20%, US$ 1,126-1,780 (incertidumbre debido a la eficacia condicional de PrEP: US$ 1,036-US$ 4,254)) se encuentra por debajo del umbral recomendado por la OMS para intervenciones costo-efectivas en Perú (<US$ 5,401/AVAD evitado)[35], mientras que es probable que sólo escenarios de priorización (algo de y alta) para una baja cobertura y el límite inferior de un escenario de alta priorización para una alta cobertura sean costo-efectivos usando un umbral más conservador sugerido por el Banco Mundial (<US$745/AVAD evitado)[44]. Ninguno de los escenarios pareció ser costo-efectivos cuando el límite inferior de la efectividad condicional de PrEP es incluida en el análisis, ni lo son los escenarios considerados bastante costo-efectivos usando el umbral del Banco Mundial de <US$149/AVAD evitado. Sin embargo, el costo por AVAD evitado es reducido sustancialmente con un alto grado de priorización. Podría ser más alto en programas de PrEP más amplios (con 20% en lugar de 5% de cobertura). En la tabla 3, presentamos los resultados costo-efectividad para nuestros seis escenarios principales junto con el costo total para un periodo de diez años para cada escenario. Para la misma inversión total, la priorización mejora la costo-efectividad. Se presenta una tabla adicional en el material suplementario donde incluimos los costos TAR posteriores evitados. Las intervenciones de PrEP podrían ser potencialmente costo-efectivas en la mayoría de los escenarios y ahorrar en costos en algunos si se incluyen los costos de tratamiento de las infecciones evitadas. Estas figuras se encuentran ilustradas en las figuras S5 a S7 en el material suplementario. La figura 5B compara la costo-efectividad de la PrEP a aquella estimada para otras intervenciones de prevención en el Perú[39] – los estimados de comparación específicos para Perú para otras intervenciones fueron derivados de Aldridge et al, específicos para Perú. Dependiendo de la estrategia de implementación, la PrEP podría ser tan costo-efectiva como el tratamiento contra ITS, alcance comunitario de HSH o TARGA. También sugiere que la PrEP debería ser considerada como una intervención adicional disponible como parte de un enfoque integral para la prevención de combinación de VIH.

**Figura 5.** Costo-efectividad de la PrEP, estimada como costo por AVAD evitado.

**Tabla 3.** Costo-efectividad y costo total de la PrEP por un periodo de diez años.

1. *Cambio de comportamiento asociado con el uso de PrEP*

Un tema importante al considerar el impacto de las intervenciones de PrEP es si los individuos usando PrEP reducirán la frecuencia con que usan condones. Estimamos el impacto de los programas de PrEP realizando diferentes supuestos acerca de cambios en el uso de condón de los individuos en PrEP, que van desde el cese completo de uso (-100% de uso de condón), hasta un leve incremento en su uso (+20% de uso de condón). En la figura 6, examinamos cómo el número de infecciones evitadas y el costo por AVAD evitado son afectados por estos cambios en el contexto de una intervención de cobertura baja o alta con un alto grado de priorización (los diagramas para los resultados de una estrategia de alta priorización, incluyendo los costos TAR posteriores, se encuentran en el material suplementario (Figura S8); los resultados para otras estrategias de priorización, incluyendo y excluyendo costos TAR posteriores, también se encuentran en el material suplementarios (Figuras S9 a S12)). En comparación a cuando no hay cambios en el comportamiento, una cesación completa del uso del condón entre aquellos en PrEP reduce el número de infecciones evitadas en diez años entre 1,500 (32%) y 2,250 (45%); e incrementa el costo por AVAD evitado en US$ 200-315 para un programa de alta cobertura (barras azules). El efecto es mayor para un programa de baja cobertura (barras verdes). Esto se debe a que sólo aquellos con el mayor riesgo de adquirir y transmitir la infección están recibiendo PrEP, de modo que cada infección intercurrente tiene mayor potencial de causar una propagación progresiva de la epidemia. El potencial de que una intervención de PrEP tenga el efecto neto de generar más nuevas infecciones es teóricamente posible pero bastante poco probable, requiriendo que la estimación empírica más baja de la eficacia intrínseca de la PrEP (40% es el límite inferior del intervalo de confianza de efectividad entre los participantes con niveles de medicamento en la sangre [95%IC 40-99]) sea verdadera y que todos los usuarios reduzcan el uso de condón en al menos un 50% (resultados no mostrados). Exploramos adicionalmente dos escenarios diferentes en los que el cambio de comportamiento podría estar correlacionado con la adherencia y hallamos que el efecto del cambio de comportamiento en la incidencia de VIH en aquellos con alta adherencia sólo es modesto (debido a que si ellos efectivamente tomas sus píldoras consistentemente, entonces el grado de protección es bastante alto). Este efecto es mayor si se halla que aquellos con una mala adherencia han cambiado su comportamiento (Figura S13A y B del material suplementario). Sin embargo, los programas de comunicación para limitar la compensación de riesgo podría ser un complemente costo-efectivo para las intervenciones de PrEP.

**Figura 6.** Impacto en la población y costo de la PrEP con respecto a los cambios en el uso de condón para una estrategia de alta priorización.

1. *Evitando un tercio de nuevas infecciones usando PrEP*

Finalmente, identificamos estrategias alternativas que se esperaría reduzcan el número de infecciones en un periodo de diez años en un tercio (Tabla 4). Con una rápida ampliación de dos años y una estrategia altamente priorizada, se requeriría un mínimo de 435,000 PrEP años-persona a un costo estimado de US$ 196M-310M en diez años. Se generaría el mismo impacto con una ampliación más lenta y una distribución uniforme, pero a un costo más elevado: US$ 277M-439M en diez años. Con todas las estrategias, un periodo más largo para alcanzar un nivel de cobertura previsto incrementa el monto de años-persona en PrEP necesaria. También incluimos en el material suplementario el costo total y costo-efectividad de las diferentes estrategias de PrEP para evitar un tercio de las infecciones, incluyendo los costos TAR posteriores evitados (Tabla S1). Mientras que los recursos necesitados aún son importantes en estos escenarios, el costo-efectividad es mejorado en gran medida.

**Tabla 4.** Escenarios para alcanzar un tercio de nuevas infecciones evitadas en diez años.

**Discusión**

Estimamos el impacto potencial de la PrEP bajo diferentes escenarios de intervención, examinando la importancia relativa de las estrategias de implementación y adherencia individual. Nuestro modelo muestra un importante impacto epidemiológico del uso de PrEP, guiado en gran medida por las características del programa de implementación – eficacia condicional de PrEP, cobertura, estrategia de priorización y tiempo para su implementación total – y comportamiento de compensación del riesgo.

En general, se prevé que la PrEP será costo-efectiva en esta población mediante las pautas de la iniciativa WHO-CHOICE, y estará en el límite de la costo-efectividad mediante las pautas más conservadoras del Banco Mundial si la intervención es priorizada a aquellos más vulnerables a la infección y cuando los costos posteriores de TARs evitados sean excluidos. Se encuentra que la mayoría de los escenarios de PrEP son costo-efectivos si estos costos son incluidos. Esto sostiene que la PrEP sea considerada entre el conjunto de intervenciones eficaces y costo-efectivas que podrían ser incluidas en una intervención integral para la prevención de combinación de VIH para HSH en este entorno. De acuerdo con la significativa heterogeneidad en comportamientos de riesgo entre HSH y mujeres trans, nuestros resultados muestran que priorizar a las personas en situaciones de mayor riesgo (por ejemplo, mujeres trans) cuando se implementa la PrEP tendría el mayor impacto. Mientras que la compensación del riesgo podría reducir el impacto de la PrEP, nuestros resultados sugieren que bajo una variedad de supuestos, aún es probable que la PrEP sea costo-efectiva.

Una mayor cobertura fortalece el impacto en la incidencia, con una rápida ampliación de la intervención incrementando el impacto en diez años. Sin embargo, la implementación de la PrEP por separado y a una escala que detenga la epidemia requeriría más recursos de los que han estado disponibles. Para reducir las nuevas infecciones en un tercio, el costo total de introducir la PrEP ascenderá a al menos US$ 20M en promedio por año (más de US$200M en diez años). Como una comparación, el gasto total de VIH en Perú para el año 2009 se reportó que estuvo justo por encima de los US$ 40M[45]. Además, si bien el Perú ha sido un importante receptor de ayuda de El Fondo Mundial de Lucha contra el SIDA, la Tuberculosis y la Malaria en América Latina, el monto total aprobado en concesiones para el VIH a la fecha se encuentra justo por encima de los US$ 85M[46].

Estos son unos de los primeros resultados para cuantificar el impacto potencial en la población de la PrEP incorporando data del único ensayo clínico llevado a cabo en una población HSH. Resaltamos la necesidad de representar heterogeneidad en el comportamiento de adherencia y una alta eficacia intrínseca de la PrEP de ser usada. También es el primer modelo en examinar el impacto entre HSH y mujeres trans en un país de ingresos bajos y medios.

Nuestros resultados son ampliamente consecuentes con trabajos de modelamiento previos en el impacto de la PrEP entre HSH en los EE.UU. donde la mayoría de los autores han encontrado que mientras la PrEP puede ser costo-efectiva bajo ciertos supuestos, la inversión necesaria sigue siendo muy alta. Por ejemplo, Desai et al.[47] encontraron que un programa de PrEP fue costo-efectivo bajo la mayoría de las variaciones en eficacia y adherencia – era posible un alto impacto para una eficacia del 70% y adherencia del programa del 50%. Paltiel et al. también encontraron que la PrEP podría tener un efecto sustancial en el riesgo de por vida de infección por VIH, especialmente entre poblaciones más jóvenes, y que podría ser una intervención de ahorro de costos con niveles de eficacia del medicamento por encima del 70% un costo de PrEP anual de US$ 2,500 o menos [48]. Recientemente, Koppenhaver et al.[49] hallaron que aunque reducciones en nuevos casos de VIH seguidos a la introducción de la PrEP entre los HSH en Nueva York llevaron a reducciones sustanciales en los costos de tratamiento, estos ahorros fueron ampliamente contrarrestados por incrementos en los costos de la PrEP. Los autores asumieron ambos costos elevados para la PrEP que incluyen costos de implementación de la PrEP y aquellos para tenofovir/emtricitabina (valorado en $22/día) y una cobertura del 100% de todos los susceptibles. Juusola et al.[50] variaron la cobertura de HSH susceptibles y también incluyeron altos costos para la PrEP consistente con los costos de EE.UU. Los autores también concluyeron que “la PrEP en la población HSH general podía prevenir un número sustancial de infecciones por VIH, pero es caro”.

Los resultados de estudios de modelamiento para uso de la PrEP en epidemias generalizadas han sido contradictorios. Abbas et al.[51] encontraron que con una alta efectividad, la PrEP podía tener un impacto beneficioso cuando se dirige a aquellos con mayor riesgo si no llevaba a un incremento de los comportamientos de riesgo. Recientemente, Pretorius et al.[40] hallaron que una intervención de PrEP era costo-efectiva bajo los umbrales de la iniciativa WHO-CHOICE pero concluyeron que no proporcionada una buena relación costo-calidad cuando se comparaba con la ampliación del TAR. Hallett et al[52] hallaron que el uso de la PrEP en parejas serodiscordantes es una alternativa costo-efectiva, especialmente en parejas con incremento de comportamiento riesgoso. Aunque nuestros hallazgos no pueden ser directamente comparados con estos, dado que los entornos difieren en gran medida, una explicación para los diferentes resultados obtenidos podría ser la diferencia en los supuestos de incidencia (ratios más altos de incidencia entre HSH llevan a que las intervenciones de prevención tengan un mayor valor).

Más allá del equilibrio necesario de consideraciones de heterogeneidad con disponibilidad de data y la relevancia programática de distinguir sub-grupos particulares, nuestro estudio tiene limitaciones adicionales. El modelo está limitado por la necesidad de capturar una variación importante y la complejidad en el comportamiento sexual de esta población en un marco simplificado. Para justificar esto, hemos aplicado un procedimiento de calibración del modelo Bayesiano. Debido a la gran cantidad de data de prevalencia consistente disponible entre HSH y mujeres trans en Lima, el mejor ajuste se usó en nuestro análisis. Sin embargo, exploramos el efecto potencial de la incertidumbre alrededor de este escenario epidemiológico en nuestros resultados principales (es decir, la costo-efectividad de la PrEP a través de todos los escenarios programáticos) y observamos que, aunque el rango es amplio, no cambia el mensaje principal obtenido de este análisis. El modelo no captura la posibilidad de una evolución de resistencia al medicamento generada por individuos usando la PrEP luego de una infección intercurrente, lo cual ha sido una preocupación. Sin embargo, varios modelos sugieren que la cantidad de resistencia generada de esta manera (individuos usando PrEP luego de una infección intercurrente) será pequeña en comparación a aquella generada por el TAR en sí[53], siempre que los individuos en PrEP sean monitoreados frecuentemente. Por consiguiente, nuestros resultados sugieren que sólo 4 de cada 1000 años-persona de PrEP estarían mal asignados a individuos infectados (en un escenario de baja cobertura, alta priorización) de los individuos en PrEP son monitoreados cada tres meses. Esto incrementa a 8 de cada 1000 años-persona de PrEP si la demora entre los monitoreos se establece para cada seis meses (resultados no se muestran). Por otra parte, Supervie et al.[54] recientemente usaron un modelo matemático para explorar la resistencia en el contexto de uso de PrEP en la comunidad HSH de San Francisco, mostrando que si los comportamientos de riesgo no incrementan, entonces la resistencia transmitida disminuiría con la PrEP.

Además, la validez de los AVADs como una medida agregada de efectividad depende de supuestos hechos para el peso de la discapacidad, el descuento, peso de la edad y esperanza de vida. Seguimos prácticas estándares con respecto a estos supuestos buscando incrementar la comparabilidad de nuestro análisis mientras proporcionamos resultados significativos para los formuladores de políticas. Sin embargo, reconocemos que los estándares supuestos acerca de los AVADs evitados gracias a la prevención de la transmisión de VIH no consideran completamente las fuertes preferencias por permanecer no infectados por el VIH dada la carga de una terapia diaria de por vida y el estigma. Adicionalmente, limitamos el cálculo de los AVADs totales evitados a una función del número de infecciones evitadas durante el periodo de intervención de diez años, asumiendo que el 80% de estos habrían recibido tratamiento de otra manera. Al hacer esto, proporcionamos una estimación conservadora de costo-efectividad de la PrEP (es decir, no incluimos los ahorros debido a los costos de tratamiento evitados). Sin embargo, esto también podría representar una aproximación optimista de los beneficios potenciales dado que asumimos que esas infecciones evitadas durante la intervención no sucederán luego. Probamos esto corriendo el modelo por 60 años adicionales luego del final de la intervención y encontramos que el número de infecciones evitadas en general es mayor que aquel observado durante el periodo de intervención (resultados disponibles en el Texto S1). La propagación de la infección es un proceso dinámico, detener las infecciones durante un periodo protege a otros de ser infectados más adelante en una manera similar a como lo hacen las vacunas. No usamos esta información para producir resultados para asegurar la comparabilidad con otros análisis de costo-efectividad que usan modelos matemáticos de la transmisión del VIH. Adicionalmente, al reconocer que realizamos un gran número de supuestos para estimar el efecto de la PrEP durante los diez años de la intervención, preferimos no extrapolar los supuestos de comportamiento posteriormente.

Otra limitación de este estudio es que las interacciones entre la PrEP y el tratamiento temprano para la prevención de la transmisión progresiva no se consideran completamente. Queda por aprender cómo la PrEP y los programas de tratamiento interactuarán en la práctica. Aunque la capacidad para fabricar medicamentos antirretrovirales, incluyendo formulaciones genéricas de los agentes más populares, no ha sido una limitante, el financiamiento disponible para adquirir estos medicamentos sí lo ha sido. Teóricamente, la PrEP podría competir con la implementación del tratamiento usando un financiamiento limitado o capacidad clínica limitada, y esto debe ser evitado. De manera alternativa, la PrEP podría habilitar programas de tratamiento al permitir mayores descuentos por volumen en los precios y costos de medicamentos, al incrementar la cobertura de pruebas, fomentando la retención en el tratamiento mediante la desestigmatización de los medicamentos antirretrovirales y de las personas que los usan, o fomentando apoyo popular y político para atraer más financiamiento para iniciativas contra el VIH/SIDA. Ante la ausencia de información acerca de cómo estas variadas posibilidades se desarrollarán, este documento ha tenido como objetivo evaluar el impacto potencial de la PrEP como una estrategia de prevención entre una población con relativamente alto riesgo de infección y explorar el efecto de diferentes factores programáticos tales como la priorización y cobertura. Mientras que el tratamiento extendido se asume en este modelo en el contexto de acceso universal a TAR de acuerdo a las guías actuales, un análisis detallado de cómo la PrEP podría afectar la cobertura del tratamiento se encuentra más allá del alcance de la información disponible en este momento.

En conclusión, hemos mostrado que si se prioriza a grupos clave y siguiendo un rápido incremento de la cobertura, la PrEP podría ser una intervención costo-efectiva para las poblaciones HSH y mujeres trans en Lima, Perú. A pesar de la costo-efectividad, en niveles aparentemente factibles de cobertura y captación, se requerirán gastos y recursos humanos considerables para generar una reducción significativa en la incidencia. Estos gastos no deberían ser considerados a menos que servicios de TAR bien realizados ya estén en su lugar, lo cual no es el caso en todos lados en Perú y mucho menos aún a través de la región. Sin embargo, si se aseguran tales condiciones, un programa de PrEP estratégicamente implementado podría realizar una contribución significativa como una parte en un paquete de combinación de intervenciones de prioridad, bien implementadas, para poblaciones HSH/mujeres trans en epidemia concentrada luego de un incremento de la cobertura de TAR.

**References**

1. AVAC Global advocacy for HIV prevention: [http://www.avac.org](http://www.avac.org/) (accessed July 2012)

2. Grant RM, Lama JR, Anderson PL, McMahan V, Liu AY, et al. (2010) Preexposure chemoprophylaxis for HIV prevention in men who have sex with men. N Engl J Med 363: 2587-2599.

3. CDC (2011) Interim Guidance: Preexposure Prophylaxis for the Prevention of HIV Infection in Men Who Have Sex with Men. MMWR Morb Mortal Wkly Rep 60: 65-68.

4. Southern African HIV Clinicians Society Consensus Committee (2012) Guidelines for the safe use of pre-exposure prophylaxis in men who have sex with men who are at risk for HIV infection. S Afr J HIV Med 13: 40-55.

5. McCormack S, Fidler S, Fisher M (2012) The British HIV Association/British Association for Sexual Health and HIV Position Statement on pre-exposure prophylaxis in the UK. Int J STD AIDS 23: 1-4.

6. WHO (2012) Guidance on oral pre-exposure prophylaxis (PrEP) for serodiscordant couples, men and transgender women who have sex with men at high risk of HIV: Recommendations for use in the context of demonstration projects. <http://wwwwhoint/hiv/pub/guidance_prep/en/indexhtml> (accessed August 2012)

7. FDA (16 July 2012) Press release: FDA approves first drug for reducing the risk of sexually acquired HIV infection. US Food and Drug Administration. <http://www.fda.gov/NewsEvents/Newsroom/PressAnnouncements/ucm312210.htm> (accessed July 2012)

8. Baeten JM, Donnell D, Ndase P, Mugo NR, Campbell JD, et al. (2012) Antiretroviral Prophylaxis for HIV Prevention in Heterosexual Men and Women. N Engl J Med 367(399-410.

9. Thigpen MC, Kebaabetswe PM, Paxton LA, Smith DK, Rose CE, et al. (2012 ) Antiretroviral Preexposure Prophylaxis for Heterosexual HIV Transmission in Botswana. N Engl J Med 367: 423-434.

10. Van Damme L, Corneli A, Ahmed K, Agot K, Lombaard J, et al. (2012) Preexposure Prophylaxis for HIV Infection among African Women. N Engl J Med 367: 411-422.

11. Abdool Karim Q, Abdool Karim SS, Frohlich JA, Grobler AC, Baxter C, et al. (2010) Effectiveness and safety of tenofovir gel, an antiretroviral microbicide, for the prevention of HIV infection in women. Science 329: 1168-1174.

12. MTN (September 2011) PRESS RELEASE: Microbicide Trials Network Statement on Decision to Discontinue Use of Oral Tenofovir Tablets in VOICE, a Major HIV Prevention Study in Women. <http://www.mtnstopshiv.org/news/studies/mtn003> (accessed November 2011)

13. Stover J (2011) HIV models to inform health policy. Current Opinion in HIV and AIDS 6: 108–113.

14. Beyrer C, Baral SD, Walker D, Wirtz AL, Johns B, et al. (2010) The expanding epidemics of HIV type 1 among men who have sex with men in low- and middle-income countries: diversity and consistency. Epidemiol Rev 32: 137-151.

15. Caceres CF, Konda K, Segura ER, Lyerla R (2008) Epidemiology of male same-sex behaviour and associated sexual health indicators in low- and middle-income countries: 2003-2007 estimates. Sex Transm Infect 84 Suppl 1: i49-i56.

16. Caceres CF, Konda KA, Salazar X, Leon SR, Klausner JD, et al. (2008) New populations at high risk of HIV/STIs in low-income, urban coastal Peru. AIDS Behav 12: 544-551.

17. Clark JL, Konda KA, Segura ER, Salvatierra HJ, Leon SR, et al. (2008) Risk factors for the spread of HIV and other sexually transmitted infections among men who have sex with men infected with HIV in Lima, Peru. Sex Transm Infect 84: 449-454.

18. Clark JL, Caceres CF, Lescano AG, Konda KA, Leon SR, et al. (2007) Prevalence of same-sex sexual behavior and associated characteristics among low-income urban males in Peru. PLoS One 2: e778.

19. Sanchez J, Lama JR, Kusunoki L, Manrique H, Goicochea P, et al. (2007) HIV-1, sexually transmitted infections, and sexual behavior trends among men who have sex with men in Lima, Peru. J Acquir Immune Defic Syndr 44: 578-585.

20. Sanchez J, Lama JR, Peinado J, Paredes A, Lucchetti A, et al. (2009) High HIV and ulcerative sexually transmitted infection incidence estimates among men who have sex with men in Peru: awaiting for an effective preventive intervention. J Acquir Immune Defic Syndr 51 Suppl 1: S47-51.

21. Silva-Santisteban A, Raymond HF, Salazar X, Villayzan J, Leon S, et al. (2011) Understanding the HIV/AIDS Epidemic in Transgender Women of Lima, Peru: Results from a Sero-Epidemiologic Study Using Respondent Driven Sampling. AIDS Behav.

22. Caceres C, Segura ER (2011) CPOS study - baseline data. Unpublished data.

23. Abu-Raddad LJ, Patnaik P, Kublin JG (2006) Dual infection with HIV and malaria fuels the spread of both diseases in sub-Saharan Africa. Science 314: 1603-1606.

24. Hallett TB, Singh K, Smith JA, White RG, Abu-Raddad LJ, et al. (2008) Understanding the impact of male circumcision interventions on the spread of HIV in southern Africa. PLoS One 3: e2212.

25. Strathdee SA, Hallett TB, Bobrova N, Rhodes T, Booth R, et al. (2010) HIV and risk environment for injecting drug users: the past, present, and future. Lancet 376: 268-284.

26. Hollingsworth TD, Anderson RM, Fraser C (2008) HIV-1 transmission, by stage of infection. J Infect Dis 198: 687-693.

27. Egger M, May M, Chene G, Phillips AN, Ledergerber B, et al. (2002) Prognosis of HIV-1-infected patients starting highly active antiretroviral therapy: a collaborative analysis of prospective studies. Lancet 360: 119-129.

28. Alkema L, Raftery AE, Clark SJ (2007) Probabilistic projections of HIV prevalence using Bayesian melding. The Annals of Applied Statistics: 229-248.

29. Poole D, Raftery AE (2000) Inference for deterministic simulation models: The Bayesian melding approach. Journal of the American Statistical Association 95: 1244-1255.

30. Blower S, Dowlatabadi H (1994) Sensitivity and uncertainty analysis of complex models of disease transmission: an HIV model, as an example. International Statistical Review 62: 229-243.

31. Anderson PL, Liu A, Buchbinder S, Lama JR, Guanira J, et al. (2012) Intracellular Tenofovir-diphosphate (TFV-DP) Concentrations Associated with PrEP Efficacy in Men who have Sex with Men (MSM) from iPrEx. 19th Conference on Retroviruses and Opportunistics Infections Seattle.

32. UNAIDS (2009) Informe Nacional de UNGASS. <http://www.unaids.org/es/dataanalysis/monitoringcountryprogress/2010progressreportssubmittedbycountries/file,57849,es..pdf> (accessed November 2011)

33. UNAIDS (February 2004) Costing Guidelines for HIV/AIDS Intervention Strategies For use in estimating Resource Needs, Scaling-up and Strategic Planning in the Asia/Pacific region.

34. Gilead <http://www.gilead.com/enabling_access> (accessed November 2011)

35. WHO-CHOICE Cost effectiveness analysis guidelines <http://www.who.int/choice/en/> (accessed November 2011)

36. Fox-Rushby JA, Hanson K (2001) Calculating and presenting disability adjusted life years (DALYs) in cost-effectiveness analysis. Health Policy Plan 16: 326-331.

37. Lopez de Castilla D, Verdonck K, Otero L, Iglesias D, Echevarria J, et al. (2008) Predictors of CD4+ cell count response and of adverse outcome among HIV-infected patients receiving highly active antiretroviral therapy in a public hospital in Peru. Int J Infect Dis 12: 325-331.

38. WHO Global burden of disease 2004 update: disability weights for diseases and conditions <http://www.who.int/healthinfo/global_burden_disease/GBD2004_DisabilityWeights.pdf> (accessed November 2011)

39. Aldridge RW, Iglesias D, Caceres CF, Miranda JJ (2009) Determining a cost effective intervention response to HIV/AIDS in Peru. BMC Public Health 9: 352.

40. Pretorius C, Stover J, Bollinger L, Bacaer N, Williams B (2010) Evaluating the cost-effectiveness of pre-exposure prophylaxis (PrEP) and its impact on HIV-1 transmission in South Africa. PLoS One 5: e13646.

41. MINSA (2004) Plan de la Estrategia Sanitaria Nacional de Prevención y Control de las ITS-VIH/SIDA 2005 – 2009.

42. Shillcutt SD, Walker DG, Goodman CA, Mills AJ (2009) Cost effectiveness in low- and middle-income countries: a review of the debates surrounding decision rules. Pharmacoeconomics 27: 903-917.

43. World Bank (2012) <http://data.worldbank.org/indicator/NY.GDP.PCAP.CD> (accessed July 2012).

44. World Bank (1993) World development report. Washington, DC.

45. Aran-Matero D, Amico P, Aran-Fernandez C, Gobet B, Izazola-Licea JA, et al. (2011) Levels of spending and resource allocation to HIV programs and services in Latin America and the Caribbean. PLoS One 6: e22373.

46. Global Fund to fight AIDS, Tuberculosis and Malaria. Peru: Country Grant Portfolio. <http://portfolio.theglobalfund.org/Country/Index/PER?lang=en> (accessed November 2011)

47. Desai K, Sansom SL, Ackers ML, Stewart SR, Hall HI, et al. (2008) Modeling the impact of HIV chemoprophylaxis strategies among men who have sex with men in the United States: HIV infections prevented and cost-effectiveness. AIDS 22: 1829-1839.

48. Paltiel AD, Freedberg KA, Scott CA, Schackman BR, Losina E, et al. (2009) HIV preexposure prophylaxis in the United States: impact on lifetime infection risk, clinical outcomes, and cost-effectiveness. Clin Infect Dis 48: 806-815.

49. Koppenhaver RT, Sorensen SW, Farnham PG, Sansom SL (2011) The cost-effectiveness of pre-exposure prophylaxis in men who have sex with men in the United States: an epidemic model. J Acquir Immune Defic Syndr 58: e51-52.

50. Juusola JL, Brandeau ML, Owens DK, Bendavid E (2012) The Cost-Effectiveness of Preexposure Prophylaxis for HIV Prevention in the United States in Men Who Have Sex With Men. Ann Intern Med 156: 541-550.

51. Abbas UL, Anderson RM, Mellors JW (2007) Potential impact of antiretroviral chemoprophylaxis on HIV-1 transmission in resource-limited settings. PLoS One 2: e875.

52. Hallett TB, Baeten JM, Heffron R, Barnabas R, de Bruyn G, et al. (2011) Optimal uses of antiretrovirals for prevention in HIV-1 serodiscordant heterosexual couples in South Africa: a modelling study. PLoS Med 8: e1001123.

53. Abbas UL, Glaubius R, Mubayi A, Hood G, Mellors JW (2011) Paper # 98LB: Predicting the Impact of ART and PrEP with Overlapping Regimens on HIV Transmission and Drug Resistance in South Africa. CROI. Boston.

54. Supervie V, Garcia-Lerma JG, Heneine W, Blower S (2010) HIV, transmitted drug resistance, and the paradox of preexposure prophylaxis. Proc Natl Acad Sci U S A 107: 12381-12386.

55. Stover J, Bertozzi S, Gutierrez JP, Walker N, Stanecki KA, et al. (2006) The global impact of scaling up HIV/AIDS prevention programs in low- and middle-income countries. Science 311: 1474-1476.

**Papel del Patrocinador**

Las organizaciones de financiamiento no tuvieron un papel en la preparación, revisión o aprobación del manuscrito.

**Reconocimientos**

A los autores les gustaría agradecer a Mark Dybul (Georgetown University), Peter Piot (London School of Hygiene and Tropical Medicine), Monica Pun (Dirección General de Epidemiologia, Ministerio de Salud, Perú) y Andreas Eisingerich, Ana Wheelock, John Williams, Lesong Conteh e Ide Cremin (Imperial College London) por sus valiosos comentarios durante la preparación de este manuscrito.

**Leyendas de las figuras**

Figura 1 – Representación del modelo de mezcla sexual y posicionamiento sexual entre HSH y mujeres trans.

Leyenda: HMSM: hombres que mayormente tienen sexo con mujeres; HMSH: hombres que mayormente tienen sexo con hombres. Los hombres insertivos y receptivos siempre toman el papel insertivo y receptivo durante el sexo anal respectivamente. Los hombres versátiles toman ya sea el rol insertivo o el receptivo durante el sexo anal. Las flechas indican parejas sexuales que están siendo formadas entre individuos dentro de los grupos – el ancho muestra el número de relaciones y la dirección ilustra el posicionamiento sexual (desde insertivo hacia receptivo). Para cada categoría: los insertivos sólo formarán pareja con receptivos y versátiles (estos últimos en un rol receptivo), los versátiles formarán pareja con insertivos y tanto versátiles como receptivos, dependiendo de su rol por acto sexual; los receptivos sólo formarán pareja con insertivos o versátiles (estos últimos en un rol insertivo).

Figura 2: Corridas seleccionadas del modelo y mejor ajuste en base a la data de prevalencia para cuatro sub-grupos y la población en general.

Leyenda: Corridas seleccionadas están representadas en gris, el mejor ajuste está en rojo. Las corridas fueron seleccionadas en base a límites (líneas negras) definidos de la data de prevalencia (cruces azules) para cuatro sub-grupos y la población en general.

Figura 3 - Proporción de nuevas infecciones evitadas totales en 10 años entregando 25,000 PrEP años-persona para cada grupo por separado.

Leyenda: HMSM: hombres que mayormente tienen sexo con mujeres; HMSH: hombres que mayormente tienen sexo con hombres; ins: insertivo; rec: receptivo; ver: versatil. Las barras de error reflejan la estimación de eficacia de la iPrEX en 92%[95%IC 40 a 99]

Figura 4 -Impacto estimado de PrEP con respecto a la cobertura, adherencia y priorización de poblaciones clave.

Leyenda: Las barras de error reflejan la incertidumbre en el estimado de eficacia de la iPrEX en 92%[95%IC 40 a 99]. En esta comparación, mostramos dos escenarios (Panel A: baja cobertura en verde; Panel B: alta cobertura en azul) incluyendo tres estrategias de priorización - uniforme, donde la cobertura es la misma en cada sub-grupo; poca priorización: una mayor cobertura lograda en las poblaciones de mujeres trans en mayor riesgo y trabajadores sexuales (pero no más del 50% cubierto) en comparación con los hombres que mayormente tienen sexo con mujeres y hombres que mayormente tienen sexo con hombres; y alta priorización, donde el 90% de mujeres trans en alto riesgo y 11% de trabajadores sexuales reciben PrEP en el escenario de baja cobertura, o 90% de las de mujeres trans en alto riesgo y 90% de trabajadores sexuales, 3.9% de hombres que mayormente tienen sexo con hombres y 21.5% de hombres que mayormente tienen sexo con mujeres reciben PrEP en el escenario de alta cobertura.

Figura 5 - Costo-efectividad de la PrEP, estimada como costo por AVAD evitado.

**A.** Costo de PrEP costo por AVAD evitado con respecto a los niveles de cobertura y estrategias.

**B.** Comparación de costo PrEP por AVAD evitado con otras estrategias de prevención del VIH.

Leyenda: Las barras de error reflejan la incertidumbre en el estimado de eficacia de la iPrEX en 92%[95%IC 40 a 99]. La altura de las barras muestra la variación debida sólo a los supuestos de costo de un año persona en PrEP. AVAD, años de vida ajustados por discapacidad. En el panel A, le perfil de adherencia de iPrEX usado para estos escenarios. En verde: escenario de baja cobertura: 5%; en azul: escenario de alta cobertura: 20%. En el panel B, la variación en costo por las intervenciones refleja diferentes estrategias de cobertura asumidas y la incertidumbre en el costeo. Los valores de Aldridge, R. et al. (2009)[39] presentes en este documento fueron calculados usando supuestos del modelo GOALS sobre la cobertura y diseño de la intervención. Estos supuestos pueden ser consultados en Stover, J. et al. (2006)[55]. El costo estimado de la PrEP/AVAD fue calculado tanto para las estrategias uniformes como para las de alta priorización (incluyendo los escenarios de cobertura del 5% y 20%). Los umbrales de costo-efectividad mostrados (líneas rojas en la figura) corresponde a: 1. Banco Mundial: intervención costo-efectiva <US$745/AVAD evitado; 2. Banco Mundial: intervención altamente costo-efectiva <US$149/AVAD evitado.

Figura 6 -Impacto en la población y costo de la PrEP con respecto a los cambios en el uso de condón para una estrategia de alta priorización.

**A.** Cambio de comportamiento e impacto en la población de la PrEP.

**B.** Cambio de comportamiento y costo de PrEP por infección evitada.

Leyenda: Esta figura asume que no hay correlación entre la adherencia y el riesgo de compensación. Exploramos este asunto por separado en el material suplementario. AVAD, años de vida ajustados por discapacidad. Perfil de adherencia de iPrEX usado para estos escenarios. En verde: escenario de baja cobertura: 5%; en azul: escenario de alta cobertura: 20%. Los umbrales de costo-efectividad mostrados (líneas rojas en la figura) corresponde a: 1. Banco Mundial: intervención costo-efectiva <US$745/AVAD evitado; 2. Banco Mundial: intervención altamente costo-efectiva <US$149/AVAD evitado. La altura de las barras muestra la variación debida sólo a los supuestos de costo de un año persona en PrEP.

Tabla 1 – Definición de escenario: Impacto de la PrEP por adherencia y efectividad funcional

| **Eficacia/adherencia** | **1: iPrEX** | **2: alta adherencia** | **3: baja adherencia** |
| --- | --- | --- | --- |
| Eficacia condicional | 0.92[0.4-0.99] | 0.92[0.4-0.99] | 0.92[0.4-0.99] |
| Adherencia 1* | 0.95 | 0.95 | 0.95 |
| Adherencia 2* | 0.40 | 0.40 | 0.40 |
| Adherencia 3* | 0.15 | 0.15 | 0.15 |
| Proporción en grupo de Adherencia 1 | 0.50 | 0.60 | 0.30 |
| Proporción en grupo de Adherencia 2 | 0.00 | 0.10 | 0.10 |
| Proporción en grupo de Adherencia 3 | 0.50 | 0.30 | 0.60 |
| Efectividad funcional | 0.52 | 0.62 | 0.35 |

Leyenda: La efectividad funcional es una función de la probabilidad de transmisión, eficacia intrínseca, la adherencia a la PrEP y su distribución, afectando sólo actos sexuales sin protección, los cuales a su vez dependen del número de parejas, uso de condón promedio, número de actos sexuales por pareja (todos los valores derivados de data de iPrEX[2]). *El estudio iPrEX reportó una distribución de participantes por rangos de adherencia. Incluimos una estimación puntual dentro de esos rangos.

Tabla 2 – Definición de escenario: Impacto de la PrEP por cobertura y priorización

| **Cobertura** | | **1: baja cobertura (5%)** | **2: alta cobertura (20%)** |
| --- | --- | --- | --- |
| Cobertura uniforme | general | 0.05 | 0.2 |
|  | HMSM | 0.05 | 0.2 |
|  | HMSH | 0.05 | 0.2 |
|  | HSM | 0.05 | 0.2 |
|  | Trans | 0.05 | 0.2 |
| Algo de priorización | general | 0.05 | 0.2 |
|  | HMSM | 0 | 0.462 |
|  | HMSH | 0 | 0.085 |
|  | HSM | 0.·26 | 0.5 |
|  | Trans | 0.5 | 0.5 |
| Alta priorización | general | 0.05 | 0.2 |
|  | HMSM | 0 | 0.215 |
|  | HMSH | 0 | 0.039 |
|  | HSM | 0.11 | 0.9 |
|  | Trans | 0.9 | 0.9 |

Leyenda: HMSM: hombres que mayormente tienen sexo con mujeres; HMSH: hombres que mayormente tienen sexo con hombres; HTS: hombres trabajadores sexuales; trans: mujeres trans en alto riesgo.

Tabla 3 – Costo-efectividad y costo total de la PrEP en 10 años.

| **Distribución** | **Cobertura** | **Total AP** | **Costo/AVAD (1)** | **Costo/AVAD (2)** | **Costo total** |
| --- | --- | --- | --- | --- | --- |
| Uniforme | 0.05 | 45,325 | [1,076 -1,702] | [419-4,182] | 23,795,696-37,619,863 |
| Uniforme | 0.20 | 182,596 | [1,125-1,779] | [428-4,254] | 95,863,381-151,555,440 |
| Algo de priorización | 0.05  (0,0,0.26,0.5) | 44,925 | [447-707] | [159-1,596] | 23,852,467-37,709,615 |
| Algo de priorización | 0.20  (0.04,0.08,0.5,0.5) | 187,116 | [886-1,400] | [397-3,133] | 96,398,735-152,401,810 |
| Alta priorización | 0.05  (0,0,0.11,0.9) | 44,002 | [403-637] | [163-1,553] | 23,877,220-37,748,747 |
| Alta priorización | 0.20  (0.21,0.03,0.9,0.9) | 183,851 | [665-1,052] | [310-2,258] | 96,071,585-151,884,601 |

Leyenda: Costos TAR posteriores evitados no incluidos. Total AP, número total de años-persona en PrEP en diez años; Costo/AVAD: costo en US$ por años de vida ajustados por discapacidad. Costo/AVAD (1) los valores en paréntesis se refieren a la variación en el costo/AVAD debido sólo a los supuestos de costo (corresponde a la altura de las barras coloreadas en la Figura 5A). Costo/AVAD (2) los valores muestran en paréntesis la variación debido a la incertidumbre total – incluyendo los supuestos tanto de costo como de eficacia de PrEP así como los supuestos epidemiológicos. Costo total: costo total de la intervención de PrEP en un periodo de 10 años; el rango refleja la incertidumbre sólo de los costos del programa de PrEP. La cobertura en la estrategia de distribución uniforme es igual en todos los grupos (HMSM – hombres que mayormente tienen sexo con mujeres, HMSH – hombres que mayormente tienen sexo con hombres, trabajadores sexuales y mujeres trans en más alto riesgo). La cobertura mostrada en las dos estrategias que involucran priorización (poca y alta) se proporcionan como una cobertura de la población en general y entre paréntesis la cobertura en cada sub-población (HMSM/HMSH/trabajadores sexuales/personas transgénero en más alto riesgo).

Tabla 4 – Escenarios para alcanzar un tercio de nuevas infecciones evitadas en 10 años.

| **Distribución** | **Ampliación** | **Cobertura** | **Total AP** | **Costo/AVAD** | **Costo total** |
| --- | --- | --- | --- | --- | --- |
| Uniforme | Dos años | 0.46 | 597,165 | [1,189-1,880] | 269,888,000-426,680,500 |
| Uniforme | Cinco años | 0.57 | 625,155 | [1,263-1,996] | 277,445,500-438,628,500 |
| Algo de priorización | Dos años | 0.40 (0.26/0.38/0.63/0.63) | 521,040 | [1,046-1,654] | 235,447,000-372,232,000 |
| Algo de priorización | Cinco años | 0.54 (0.51/0.51/0.7/0.7) | 591,730 | [997-1,879] | 262,597,000-415,153,500 |
| Alta priorización | Dos años | 0.34 (0.27/0.22/0.9/0.9) | 433,810 | [870-1,375] | 195,959,000-309,802,000 |
| Alta priorización | Cinco años | 0.49 (0.42/0.41/0.9/0.9) | 535,155 | [1,073-1,696] | 237,555,000-375,563,000 |

Leyenda: Total AP, número total de años-persona en PrEP en 10 años; Costo/AVAD: costo en US$ por años de vida ajustados por discapacidad. El rango observado en esta columna y en la columna de Costo total representa la variación observada en los costos estimados por año de PrEP; Costo total: costo total de la intervención de PrEP en diez años. Cobertura en la estrategia de distribución uniforme es igual en todos los sub-grupos (HMSM – hombres que mayormente tienen sexo con mujeres, HMSH – hombres que mayormente tienen sexo con hombres, trabajadores sexuales y mujeres trans en más alto riesgo). La cobertura mostrada en las dos estrategias que involucran priorización (poca y alta) se proporcionan como una cobertura de la población en general y entre paréntesis la cobertura en cada sub-población (HMSM/HMSH/trabajadores sexuales/personas transgénero en más alto riesgo).

**Información de Apoyo**

#### Texto S1

#### Información técnica suplementaria

#### Figura S1

#### Impacto estimado de la PrEP con respecto a cobertura, adherencia, y priorización de poblaciones clave (I. Número de infecciones evitadas/1000 AP-PrEP)

#### Leyenda: El impacto es presentado como infecciones evitadas por cada 1000 años-persona de PrEP. Las barras de error reflejan la incertidumbre en la estimación de eficacia de la PrEP al 92% [95%IC 40 a 99]. En esta comparación, les mostramos dos escenarios (Panel A: baja cobertura en verde; Panel B: alta cobertura en azul) para tres perfiles de adherencia incluyendo tres estrategias de priorización – uniforme, donde la cobertura es la misma en cada sub-grupo; poca priorización: una alta cobertura lograda en las mujeres trans en mayor riesgo y poblaciones de trabajadores del sexo (pero no más de 50% cubierto) comparado con hombres que tienen sexo mayormente con mujeres, y hombres que tienen sexo mayormente con hombres; y alta priorización, donde el 90% de mujeres trans en mayor riesgo y 11% de trabajadores del sexo reciben PrEP en el escenario de baja cobertura, o 90% de mujeres trans en mayor riesgo, 90% de trabajadores del sexo, 3.9% de hombres que tienen sexo mayormente con hombres, y 21.5% de hombres que tienen sexo mayormente con mujeres reciben PrEP en el escenario de alta cobertura.

#### Figura S2

#### Impacto estimado de la PrEP con respecto a cobertura, adherencia, y priorización de poblaciones clave (II. Proporción de infecciones evitadas en 10 años)

#### Leyenda: El impacto es presentado como porcentaje del total de infecciones que son evitadas con la PrEP. Las barras de error reflejan la incertidumbre en la estimación de eficacia de la PrEP al 92% [95%IC 40 a 99]. En esta comparación, les mostramos dos escenarios (Panel A: baja cobertura en verde; Panel B: alta cobertura en azul) para tres perfiles de adherencia incluyendo tres estrategias de priorización – uniforme, donde la cobertura es la misma en cada sub-grupo; poca priorización: una alta cobertura lograda en las mujeres trans en mayor riesgo y poblaciones de trabajadores del sexo (pero no más de 50% cubierto) comparado con hombres que tienen sexo mayormente con mujeres, y hombres que tienen sexo mayormente con hombres; y alta priorización, donde el 90% de mujeres trans en mayor riesgo y 11% de trabajadores del sexo reciben PrEP en el escenario de baja cobertura, o 90% de mujeres trans en mayor riesgo, 90% de trabajadores del sexo, 3.9% de hombres que tienen sexo mayormente con hombres, y 21.5% de hombres que tienen sexo mayormente con mujeres reciben PrEP en el escenario de alta cobertura.

#### Figura S3

#### Costo-efectividad de la PrEP, estimación como costo por AVAD evitado: incluyendo costos de TAR posteriores ahorrados.

#### A. Costos de TAR posteriores evitados incluidos en US$1,000/AP- TAR.

#### B. Costos de TAR posteriores evitados incluidos en US$3,500/AP- TAR.

#### Leyenda: AVAD, año de vida de discapacidad ajustado. Perfil de adherencia de iPrEX utilizado para estos escenarios. En verde: escenario de baja cobertura: 5%; en azul: escenario de alta cobertura: 20%. La variación en costos refleja la incertidumbre en el costeo de un año-persona en PrEP. Los umbrales de costo-efectividad presentados (línea rojas en la figura) corresponden a: 1. Banco Mundial: intervención costo-efectiva<US$745/AVAD evitado; 2. Banco Mundial: intervención altamente costo-efectiva<US$149/AVAD evitado.

#### Figura S4

#### Costo-efectividad y costo total de la PrEP durante 10 años: Costos de TAR posteriores no incluidos.

#### Leyenda: AVAD, año de vida de discapacidad ajustado. Perfil de adherencia de iPrEX utilizado para todos los escenarios. En verde: escenario de baja cobertura: 5%; en azul: escenario de alta cobertura: 20%. Las líneas son tramadas contra el eje Y de costo/AVAD evitado en US$ y contra el eje X – número total de AVADs evitados en 10 años. Los números sobre las líneas señalan los puntos de datos como sigue – 1. Baja cobertura, escenario uniforme; 2. Baja cobertura, escenario de poca priorización; 3. Baja cobertura, escenario de alta priorización; 4. Alta cobertura, escenario uniforme; 5. Alta cobertura, escenario de poca priorización; 6. Alta cobertura, escenario de alta priorización. Estos puntos de datos tienen barras de incertidumbre representando la variación en el costeo de un año-persona en PrEP. Las casillas son tramadas contra el eje de la mano derecha únicamente. Ellos representan el costo total de escenarios. La variación en costos (altura de las casillas) refleja la incertidumbre en el costeo de un año-persona en PrEP.

#### Figura S5

#### Costo-efectividad y costo total de la PrEP durante 10 años: Costos de TAR posteriores incluidos a US$1,000/AP- TAR.

#### Leyenda: AVAD, año de vida de discapacidad ajustado. Perfil de adherencia de iPrEX utilizado para todos los escenarios. En verde: escenario de baja cobertura: 5%; en azul: escenario de alta cobertura: 20%. Las líneas son tramadas contra el eje Y de costo/AVAD evitado en US$ y contra el eje X – número total de AVADs evitados en 10 años. Los números sobre las líneas señalan los puntos de datos como sigue – 1. Baja cobertura, escenario uniforme; 2. Baja cobertura, escenario de poca priorización; 3. Baja cobertura, escenario de alta priorización; 4. Alta cobertura, escenario uniforme; 5. Alta cobertura, escenario de poca priorización; 6. Alta cobertura, escenario de alta priorización. Estos puntos de datos tienen barras de incertidumbre representando la variación en el costeo de un año-persona en PrEP. Las casillas son tramadas contra el eje de la mano derecha únicamente. Ellos representan el costo total de escenarios. La variación en costos (altura de las casillas) refleja la incertidumbre en el costeo de un año-persona en PrEP.

#### Figura S6

#### Costo-efectividad y costo total de la PrEP durante 10 años: Costos de TAR posteriores incluidos a US$3,500/AP- TAR.

#### Leyenda: AVAD, año de vida de discapacidad ajustado. Perfil de adherencia de iPrEX utilizado para todos los escenarios. En verde: escenario de baja cobertura: 5%; en azul: escenario de alta cobertura: 20%. Las líneas son tramadas contra el eje Y de costo/AVAD evitado en US$ y contra el eje X – número total de AVADs evitados en 10 años. Los números sobre las líneas señalan los puntos de datos como sigue – 1. Baja cobertura, escenario uniforme; 2. Baja cobertura, escenario de poca priorización; 3. Baja cobertura, escenario de alta priorización; 4. Alta cobertura, escenario uniforme; 5. Alta cobertura, escenario de poca priorización; 6. Alta cobertura, escenario de alta priorización. Estos puntos de datos tienen barras de incertidumbre representando la variación en el costeo de un año-persona en PrEP. Las casillas son tramadas contra el eje de la mano derecha únicamente. Ellos representan el costo total de escenarios. La variación en costos (altura de las casillas) refleja la incertidumbre en el costeo de un año-persona en PrEP.

#### Figura S7

#### Costo de la PrEP con respecto a los cambios en uso del condón para una estrategia de alta priorización.

#### A. Costos de TAR posteriores evitados incluidos en US$1,000/AP- TAR.

#### B. Costos de TAR posteriores evitados incluidos en US$3,500/AP- TAR.

#### Leyenda: Esta figura asume que no hay una correlación entre la adherencia y la compensación de riesgo. Exploramos este tema separadamente en la Figura S12. AVAD, año de vida de discapacidad ajustado. Perfil de adherencia de iPrEX utilizado para estos escenarios. En verde: escenario de baja cobertura: 5%; en azul: escenario de alta cobertura: 20%. Los umbrales de costo-efectividad presentados (línea rojas en la figura) corresponden a: 1. Banco Mundial: intervención costo-efectiva<US$745/AVAD evitado; 2. Banco Mundial: intervención altamente costo-efectiva<US$149/AVAD evitado.

#### Figura S8

#### Impacto de la población de PrEP con respecto a cambios en el uso de condón.

#### A. Escenario “uniforme”

#### B. Escenario de “poca priorización”

#### Leyenda: Esta figura asume que no hay una correlación entre la adherencia y la compensación de riesgo. Exploramos este tema separadamente en la Figura S13. En verde: escenario de baja cobertura: 5%; en azul: escenario de alta cobertura: 20%. La referencia es ningún cambio en el uso de condón.

#### Figura S9

#### Costo-efectividad de la PrEP con respecto a los cambios en el uso de condón: Costos de TAR posteriores evitados no incluidos.

#### A. Escenario “uniforme”

#### B. Escenario de “poca priorización”

#### Leyenda: Esta figura asume que no hay una correlación entre la adherencia y la compensación de riesgo. Exploramos este tema separadamente en la Figura S12. AVAD, año de vida de discapacidad ajustado. Perfil de adherencia de iPrEX utilizado para estos escenarios. En verde: escenario de baja cobertura: 5%; en azul: escenario de alta cobertura: 20%. Los umbrales de costo-efectividad presentados (línea rojas en la figura) corresponden a: 1. Banco Mundial: intervención costo-efectiva<US$745/AVAD evitado; 2. Banco Mundial: intervención altamente costo-efectiva<US$149/AVAD evitado.

#### Figura S10

#### Costo-efectividad de la PrEP con respecto a los cambios en el uso de condón: Costos de TAR posteriores evitados incluidos a US$1,000/AP- TAR.

#### A. Escenario “uniforme”

#### B. Escenario de “poca priorización”

#### Leyenda: Esta figura asume que no hay una correlación entre la adherencia y la compensación de riesgo. Exploramos este tema separadamente en la Figura S12. AVAD, año de vida de discapacidad ajustado. Perfil de adherencia de iPrEX utilizado para estos escenarios. En verde: escenario de baja cobertura: 5%; en azul: escenario de alta cobertura: 20%. Los umbrales de costo-efectividad presentados (línea rojas en la figura) corresponden a: 1. Banco Mundial: intervención costo-efectiva<US$745/AVAD evitado; 2. Banco Mundial: intervención altamente costo-efectiva<US$149/AVAD evitado.

#### Figura S11

#### Costo-efectividad de la PrEP con respecto a los cambios en el uso de condón: Costos de TAR posteriores evitados incluidos a US$3,500/AP- TAR.

#### A. Escenario “uniforme”

#### B. Escenario de “poca priorización”

#### Leyenda: Esta figura asume que no hay una correlación entre la adherencia y la compensación de riesgo. Exploramos este tema separadamente en la Figura S12. AVAD, año de vida de discapacidad ajustado. Perfil de adherencia de iPrEX utilizado para estos escenarios. En verde: escenario de baja cobertura: 5%; en azul: escenario de alta cobertura: 20%. Los umbrales de costo-efectividad presentados (línea rojas en la figura) corresponden a: 1. Banco Mundial: intervención costo-efectiva<US$745/AVAD evitado; 2. Banco Mundial: intervención altamente costo-efectiva<US$149/AVAD evitado.

#### Figura S12

#### Impacto de la población de PrEP con respecto a cambios diferenciales en el uso de condón.

#### A. Sólo los buenos adherentes cambian su comportamiento

#### B. Sólo los malos adherentes cambian su comportamiento

#### Leyenda: Esta figura asume que hay una correlación entre la adherencia y la compensación de riesgo. Perfil de adherencia de iPrEX utilizado para estos escenarios. En verde: escenario de baja cobertura: 5%; en azul: escenario de alta cobertura: 20%. La referencia es ningún cambio en el uso de condón.

#### Tabla S1

#### Escenarios a conseguir un tercio de nuevas infecciones evitadas durante 10 años. Costos de A TAR posteriores evitados incluidos a US$1,000 y 3,500/AP- TAR.

Leyenda: AP Total, número total de años-persona en PrEP durante 10 años; Costo/AVAD: costo en US$ años de vida de discapacidad ajustada; Costo total: costo total de intervención PrEP durante 10 años. (1) Incluye costos de TAR posteriores evitados incluidos a US$1,000/AP- TAR. (2) Incluye costos de TAR posteriores incluidos a US$3,5000/AP- TAR. El rango observado en esta columna y en la columna de Costo total representa la variación observada en los costos por año-PrEP. Cobertura en la estrategia de distribución uniforme es igual en todos los subgrupos (HSMM – hombres que tienen sexo mayormente con mujeres, HSMH – hombres que tienen sexo mayormente con hombres, trabajadores del sexo, y mujeres trans en mayor riesgo). La cobertura presentada en ambas estrategias involucrando priorización (poca y alta) es brindada cobertura de población general y en corchetes, la cobertura en cada sub-población (HSMM/HSMH/trabajadores del sexo/mujeres trans en mayor riesgo).
