# Supplementary figures and images for: The Potential Impact of Pre-Exposure Prophylaxis for HIV Prevention among Men Who Have Sex with Men and Transwomen in Lima, Peru: A Mathematical Modelling Study
Source: PLoS Med. 2012 Oct 9;9(10):e1001323. doi: 10.1371/journal.pmed.1001323 (PMC3467261; doi:10.1371/journal.pmed.1001323)

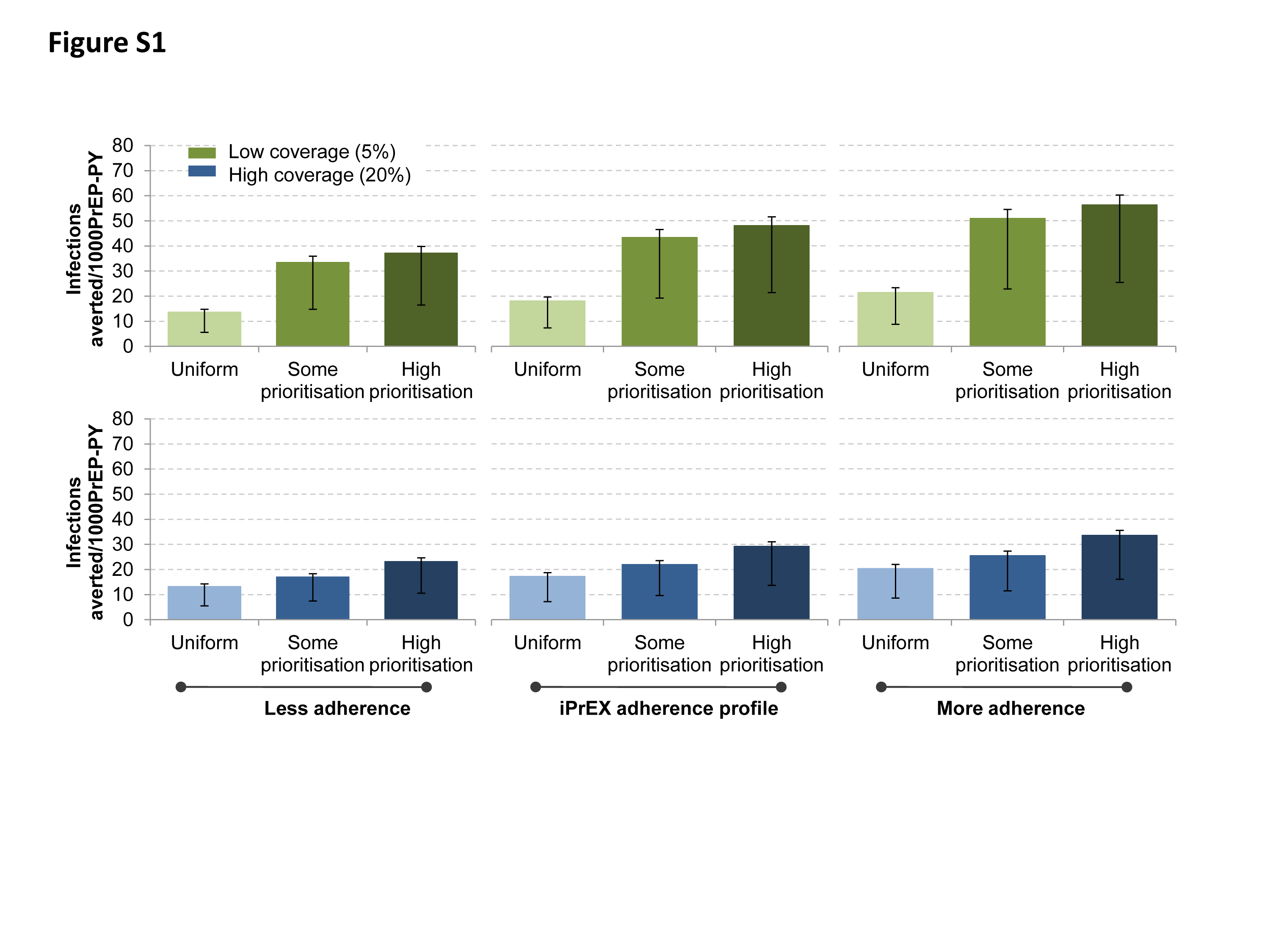

Supplement: Figure S1 — Estimated impact of PrEP with respect to coverage, adherence, and prioritisation of key populations: number of infections averted/1,000 PrEP person-years. Impact is shown as infections averted for every 1,000 PrEP person-years (PY). The error bars reflect the uncertainty in the iPrEx efficacy estimate of 92% (95% CI 40–99). In this comparison, we show two scenarios ([A]: low coverage, in green; [B]: high coverage, in blue) for three adherence profiles including three prioritisation strategies—uniform, where the coverage is the same in each subgroup; some prioritisation, where there is higher coverage achieved in the transwomen at higher risk and sex worker populations (but no more than 50% covered) than in MMSW and MMSM; and high prioritisation, where 90% of transwomen at higher risk and 11% of sex workers receive PrEP in the low coverage scenario, or 90% of transwomen at higher risk, 90% of sex workers, 3.9% of MMSM, and 21.5% of MMSW receive PrEP in the high coverage scenario. (TIFF) [file pmed.1001323.s002.tif]

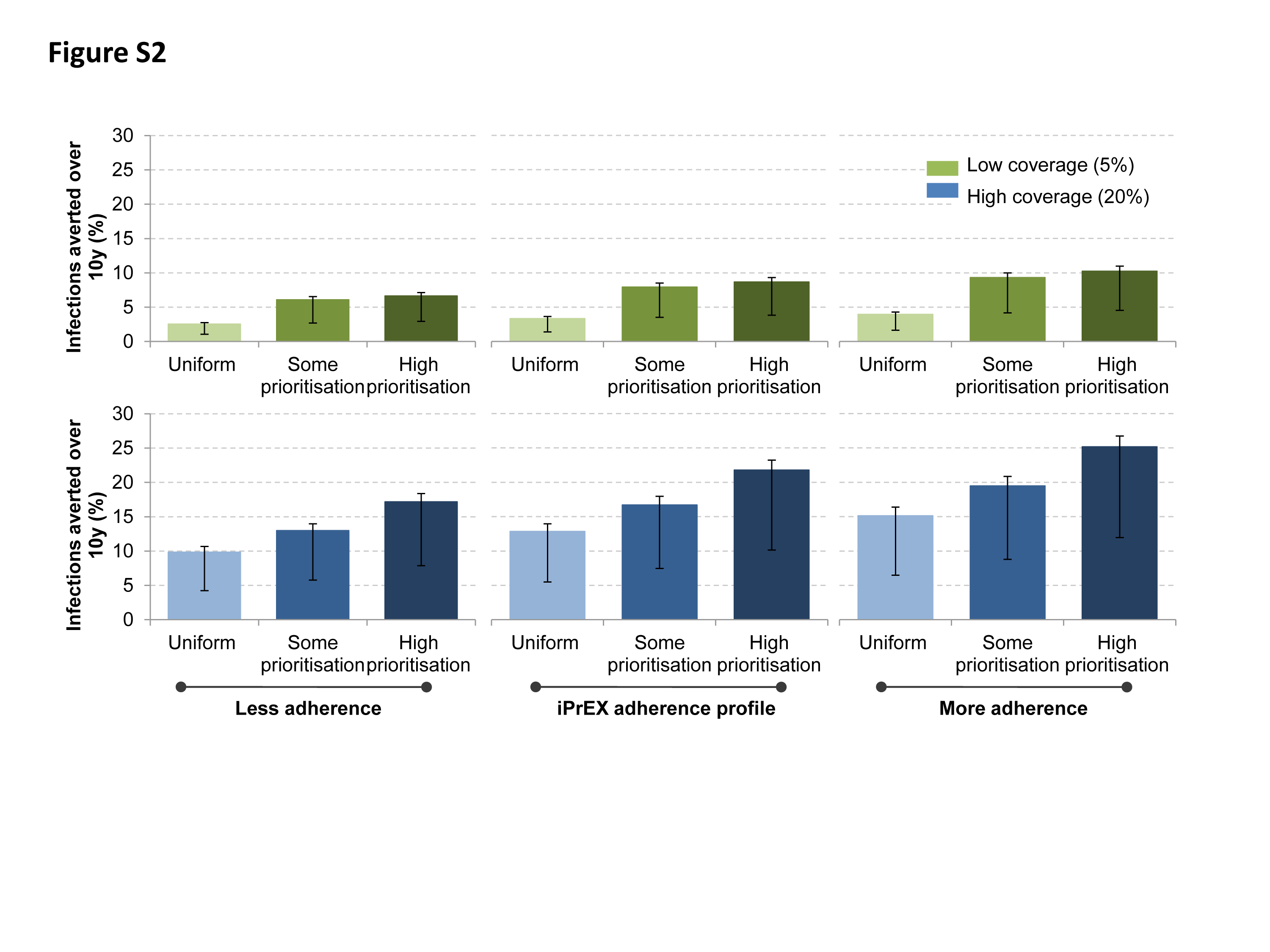

Supplement: Figure S2 — Estimated impact of PrEP with respect to coverage, adherence, and prioritisation of key populations: proportion of infections averted over 10 y. Impact is shown as percentage of total infections that are averted with PrEP. The error bars reflect the uncertainty in the iPrEx efficacy estimate of 92% (95% CI 40–99). In this comparison, we show two scenarios ([A]: low coverage, in green; [B]: high coverage, in blue) for three adherence profiles including three prioritisation strategies—uniform, where the coverage is the same in each subgroup; some prioritisation, where there is higher coverage achieved in the transwomen at higher risk and sex worker populations (but no more than 50% covered) than in MMSW and MMSM; and high prioritisation, where 90% of transwomen at higher risk and 11% of sex workers receive PrEP in the low coverage scenario, or 90% of transwomen at higher risk, 90% of sex workers, 3.9% of MMSM, and 21.5% of MMSW receive PrEP in the high coverage scenario. (TIFF) [file pmed.1001323.s003.tif]

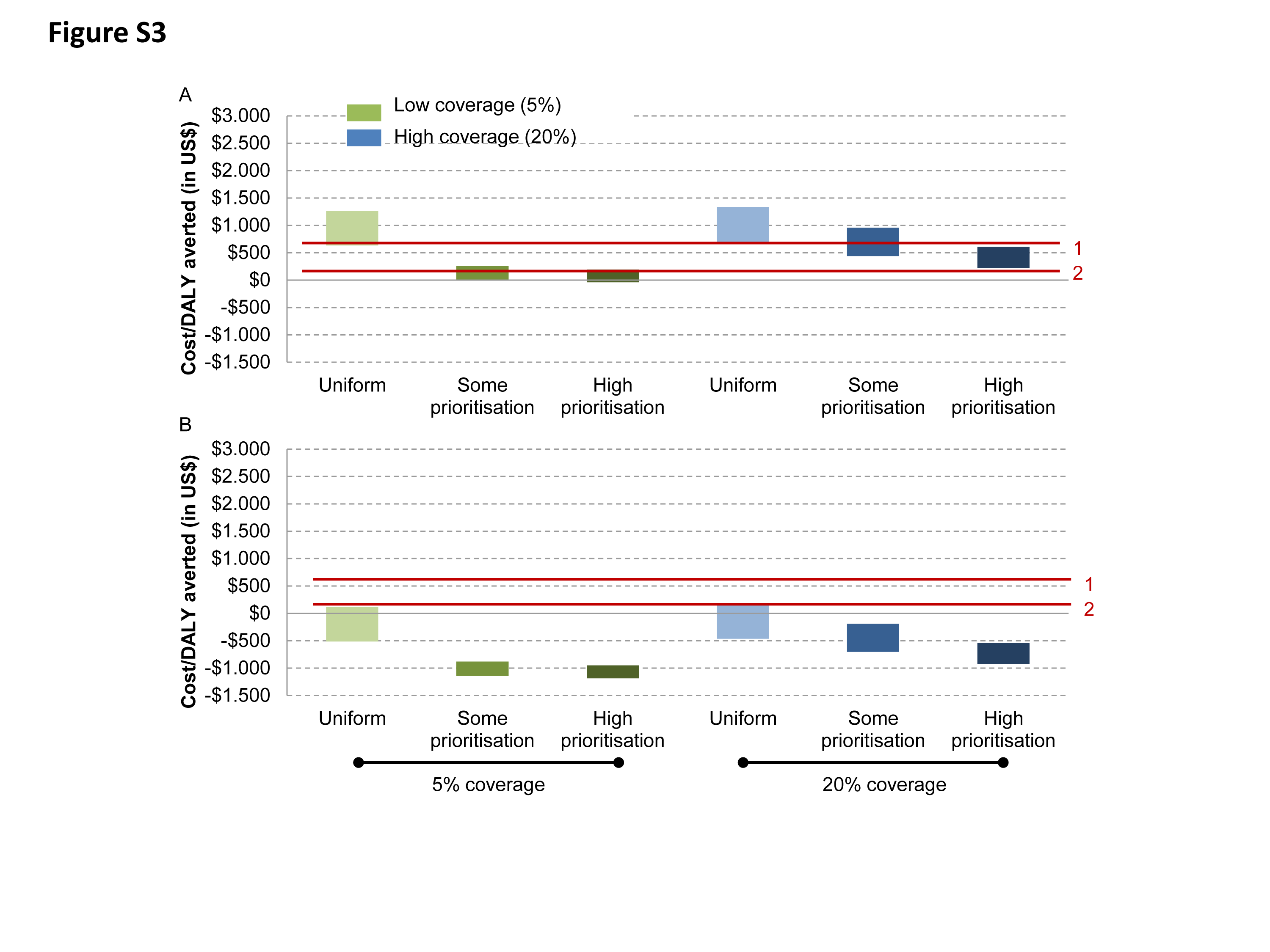

Supplement: Figure S3 — Cost-effectiveness of PrEP, estimated as cost per DALY averted: downstream ARV costs included. (A) Downstream ARV costs averted included at US$1,000/person-year on ARV drugs. (B) Downstream ARV costs averted included at US$3,500/person-year on ARV drugs. iPrEx adherence profile used for these scenarios. In green: low coverage scenario: 5%; in blue: high coverage scenario: 20%. The variation in costs reflects the uncertainty in the costing of one person-year on PrEP. The red lines correspond to (1) the World Bank threshold for a cost-effective intervention, <US$745/DALY averted, and (2) the World Bank threshold for a highly cost-effective intervention, <US$149/DALY averted. (TIFF) [file pmed.1001323.s004.tif]

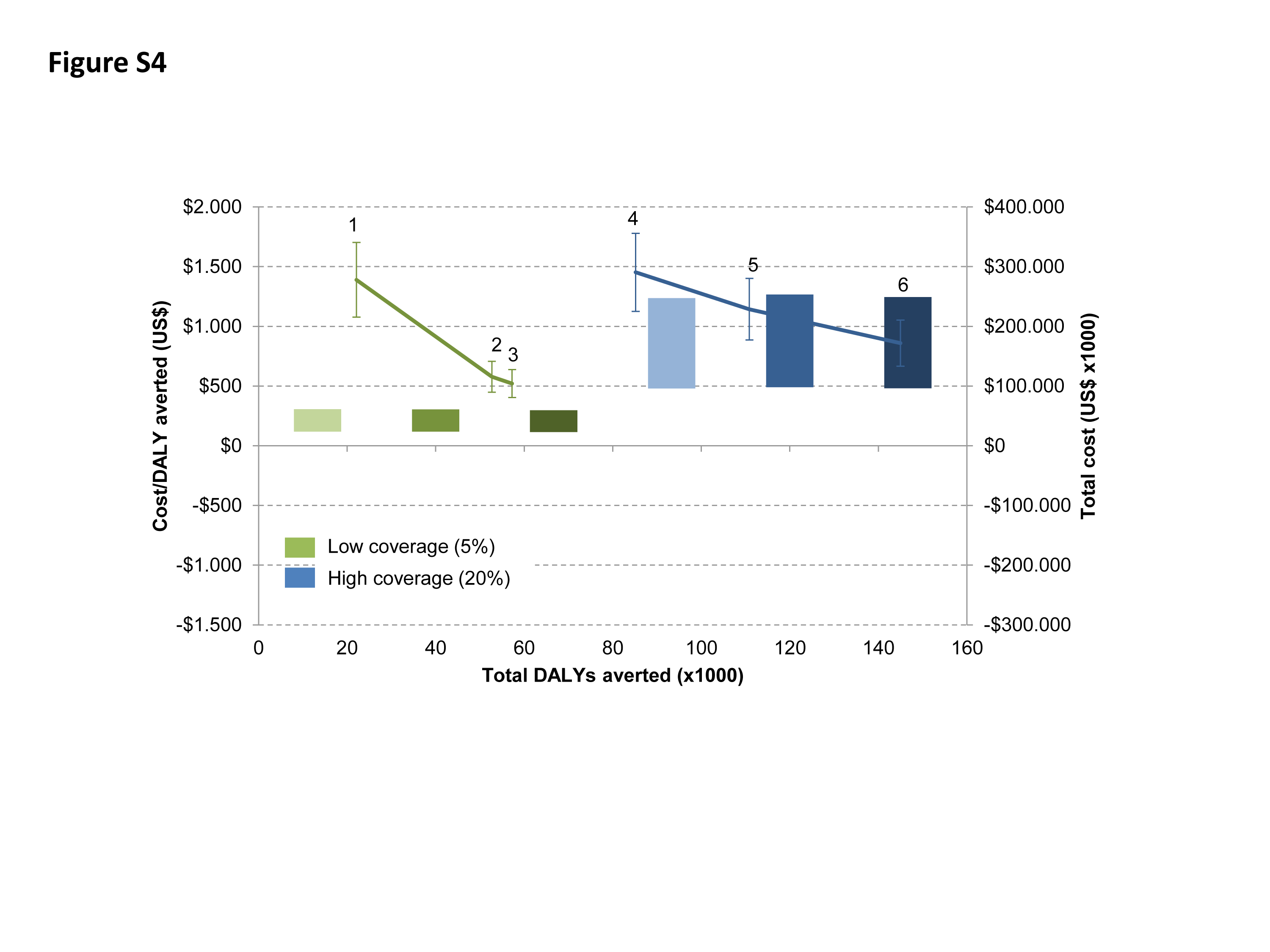

Supplement: Figure S4 — Cost-effectiveness and total cost of PrEP over 10 y: downstream ARV costs not included. iPrEx adherence profile used for all scenarios. In green: low coverage scenario: 5%; in blue: high coverage scenario: 20%. Lines are plotted against the y-axis of cost/DALY averted in US dollars and against the x-axis—total number of DALYs averted over 10 y. The numbers over the lines indicate the data points as follows: 1, low coverage, uniform scenario; 2, low coverage, some prioritisation scenario; 3, low coverage, high prioritisation scenario; 4, high coverage, uniform scenario; 5, high coverage, some prioritisation scenario; 6, high coverage, high prioritisation scenario. These data points have uncertainty bars representing the variation in the costing of one person-year on PrEP. The boxes are plotted against the right-hand axis only. They represent the total cost of scenarios. The variation in costs (height of boxes) reflects the uncertainty in the costing of one person-year on PrEP. (TIFF) [file pmed.1001323.s005.tif]

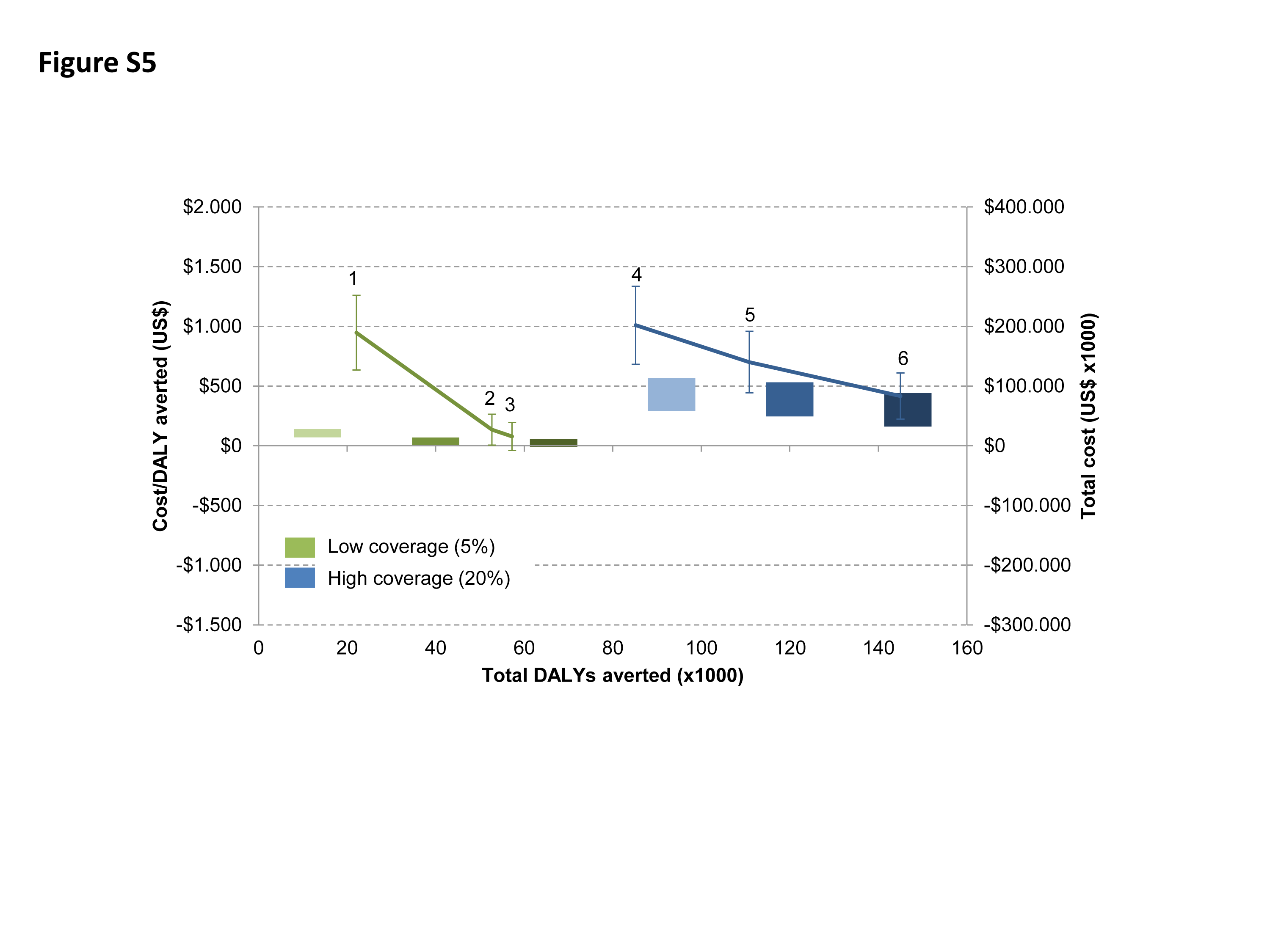

Supplement: Figure S5 — Cost-effectiveness and total cost of PrEP over 10 y: downstream ARV costs averted included at US$1,000/person-year on ARV drugs. iPrEx adherence profile used for all scenarios. In green: low coverage scenario: 5%; in blue: high coverage scenario: 20%. Lines are plotted against the y-axis of cost/DALY averted in US dollars and against the x-axis—total number of DALYs averted over 10 y. The numbers over the lines indicate the data points as follows: 1, low coverage, uniform scenario; 2, low coverage, some prioritisation scenario; 3, low coverage, high prioritisation scenario; 4, high coverage, uniform scenario; 5, high coverage, some prioritisation scenario; 6, high coverage, high prioritisation scenario. These data points have uncertainty bars representing the variation in the costing of one person-year on PrEP. The boxes are plotted against the righthand axis only. They represent the total cost of scenarios. The variation in costs (height of boxes) reflects the uncertainty in the costing of one person-year on PrEP. (TIFF) [file pmed.1001323.s006.tif]

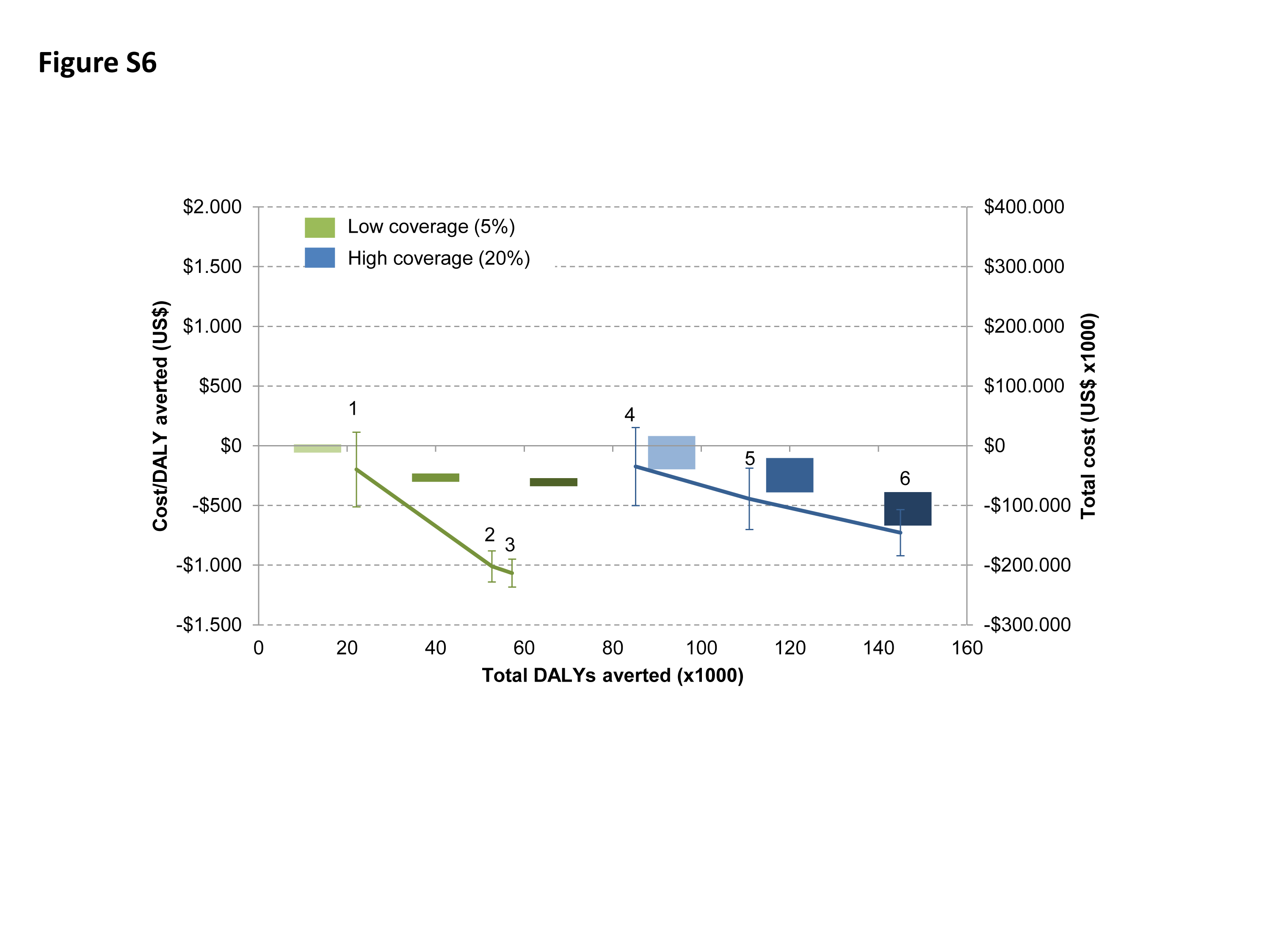

Supplement: Figure S6 — Cost-effectiveness and total cost of PrEP over 10 y: downstream ARV costs averted included at US$3,500/person-year on ARV drugs. iPrEx adherence profile used for all scenarios. In green: low coverage scenario: 5%; in blue: high coverage scenario: 20%. Lines are plotted against the y-axis of cost/DALY averted in US dollars and against the x-axis—total number of DALYs averted over 10 y. The numbers over the lines indicate the data points as follows: 1, low coverage, uniform scenario; 2, low coverage, some prioritisation scenario; 3, low coverage, high prioritisation scenario; 4, high coverage, uniform scenario; 5, high coverage, some prioritisation scenario; 6, high coverage, high prioritisation scenario. These data points have uncertainty bars representing the variation in the costing of one person-year on PrEP. The boxes are plotted against the righthand axis only. They represent the total cost of scenarios. The variation in costs (height of boxes) reflects the uncertainty in the costing of one person-year on PrEP. (TIFF) [file pmed.1001323.s007.tif]

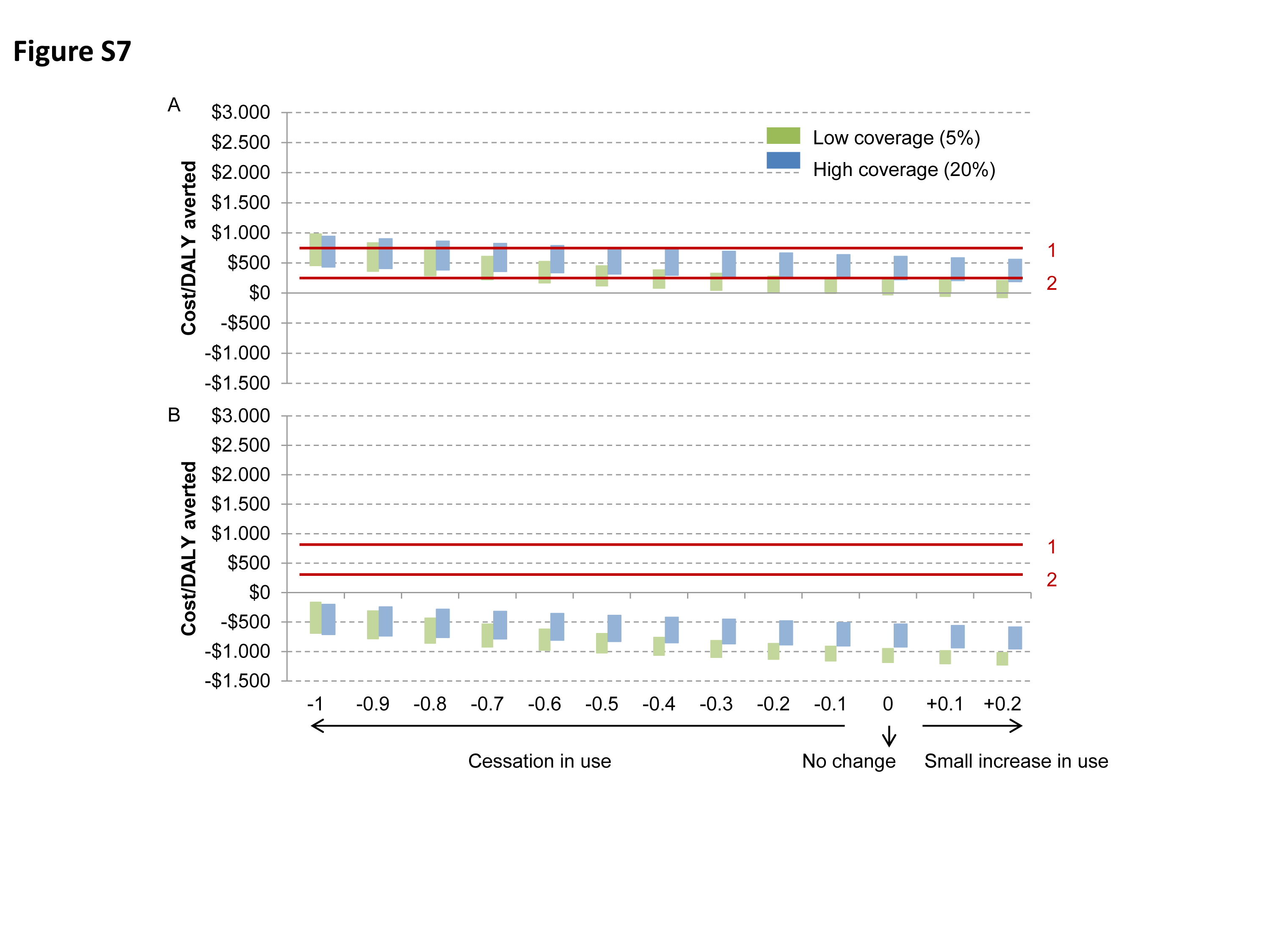

Supplement: Figure S7 — Cost of PrEP with respect to changes in condom use for a high prioritisation strategy. (A) Downstream ARV costs averted included at US$1,000/person-year on ARV drugs. (B) Downstream ARV costs averted included at US$3,500/person-year on ARV drugs. This figure assumes there is no correlation between adherence and risk compensation. We explore this issue separately in Figure S12. iPrEx adherence profile used for these scenarios. In green: low coverage scenario: 5%; in blue: high coverage scenario: 20%. The red lines correspond to (1) the World Bank threshold for a cost-effective intervention, <US$745/DALY averted, and (2) the World Bank threshold for a highly cost-effective intervention, <US$149/DALY averted. (TIFF) [file pmed.1001323.s008.tif]

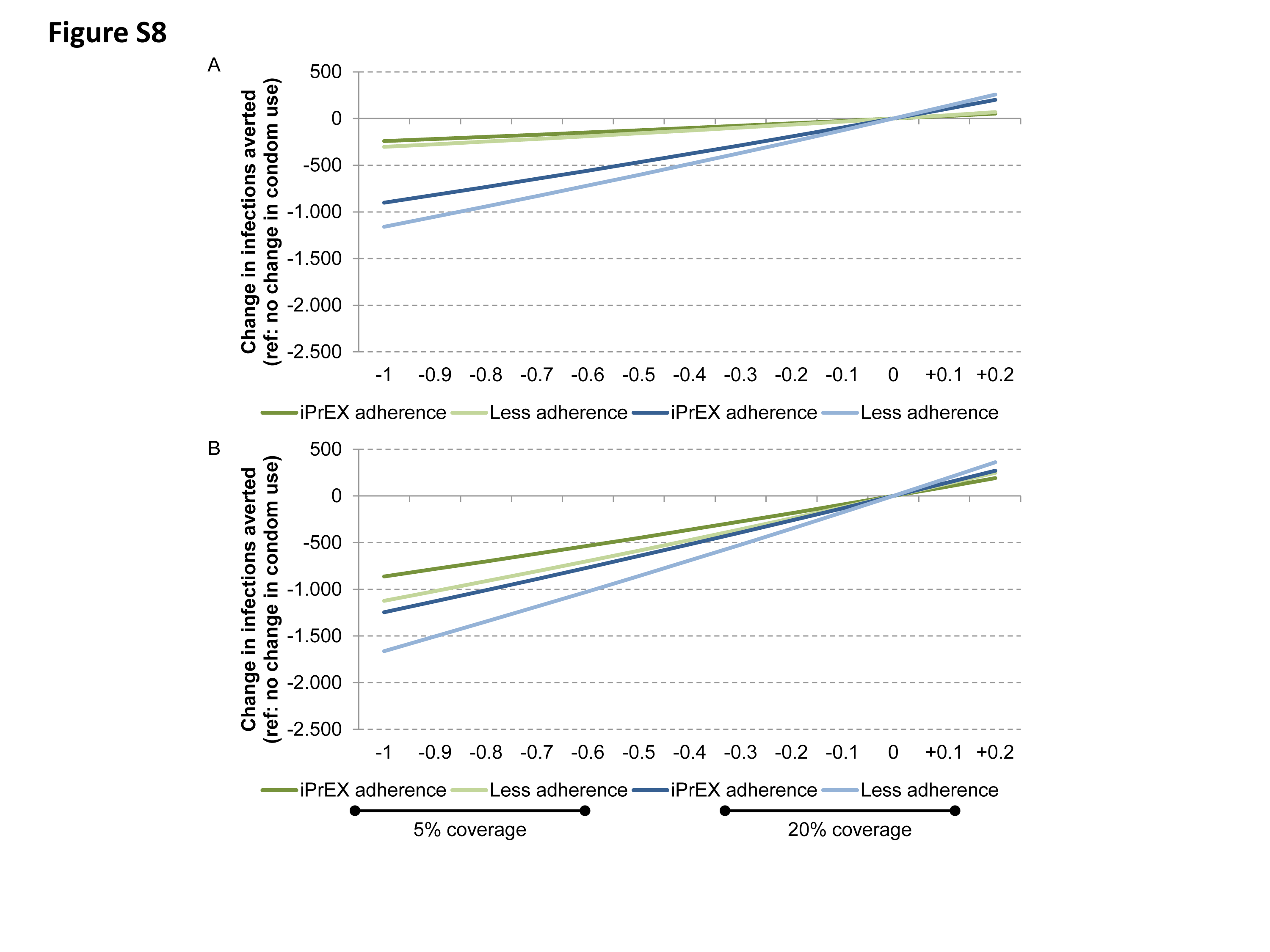

Supplement: Figure S8 — Population impact of PrEP with respect to changes in condom use. (A) “Uniform” scenario. (B) “Some prioritisation” scenario. This figure assumes there is no correlation between adherence and risk compensation. We explore this issue separately in Figure S12. In green: low coverage scenario: 5%; in blue: high coverage scenario: 20%. Reference is no change in condom use. (TIFF) [file pmed.1001323.s009.tif]

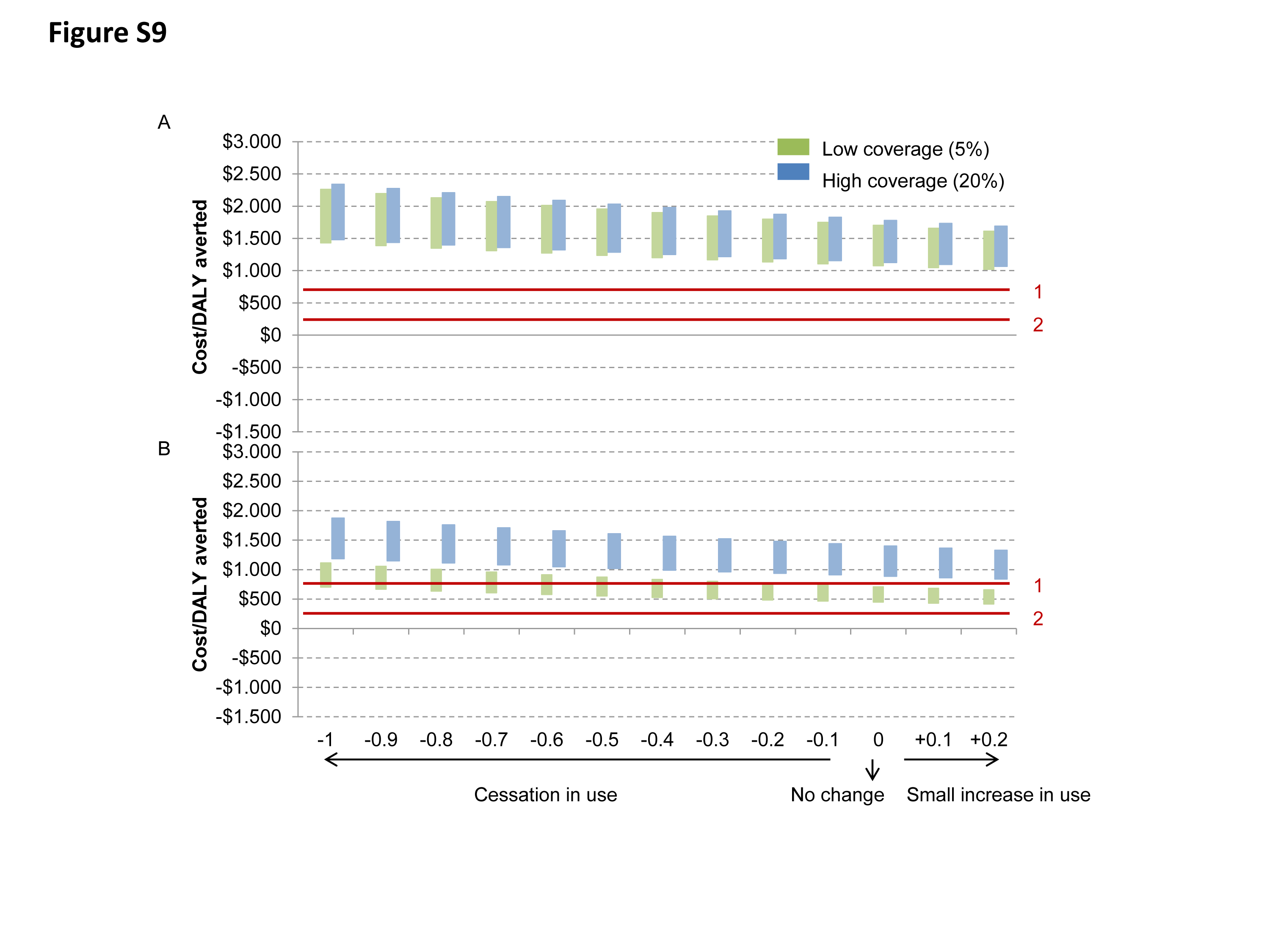

Supplement: Figure S9 — Cost-effectiveness of PrEP with respect to changes in condom use: downstream ARV costs averted not included. (A) “Uniform” scenario. (B) “Some prioritisation” scenario. This figure assumes there is no correlation between adherence and risk compensation. We explore this issue separately in Figure S12. iPrEx adherence profile used for these scenarios. In green: low coverage scenario: 5%; in blue: high coverage scenario: 20%. The red lines correspond to (1) the World Bank threshold for a cost-effective intervention, <US$745/DALY averted, and (2) the World Bank threshold for a highly cost-effective intervention, <US$149/DALY averted. (TIFF) [file pmed.1001323.s010.tif]

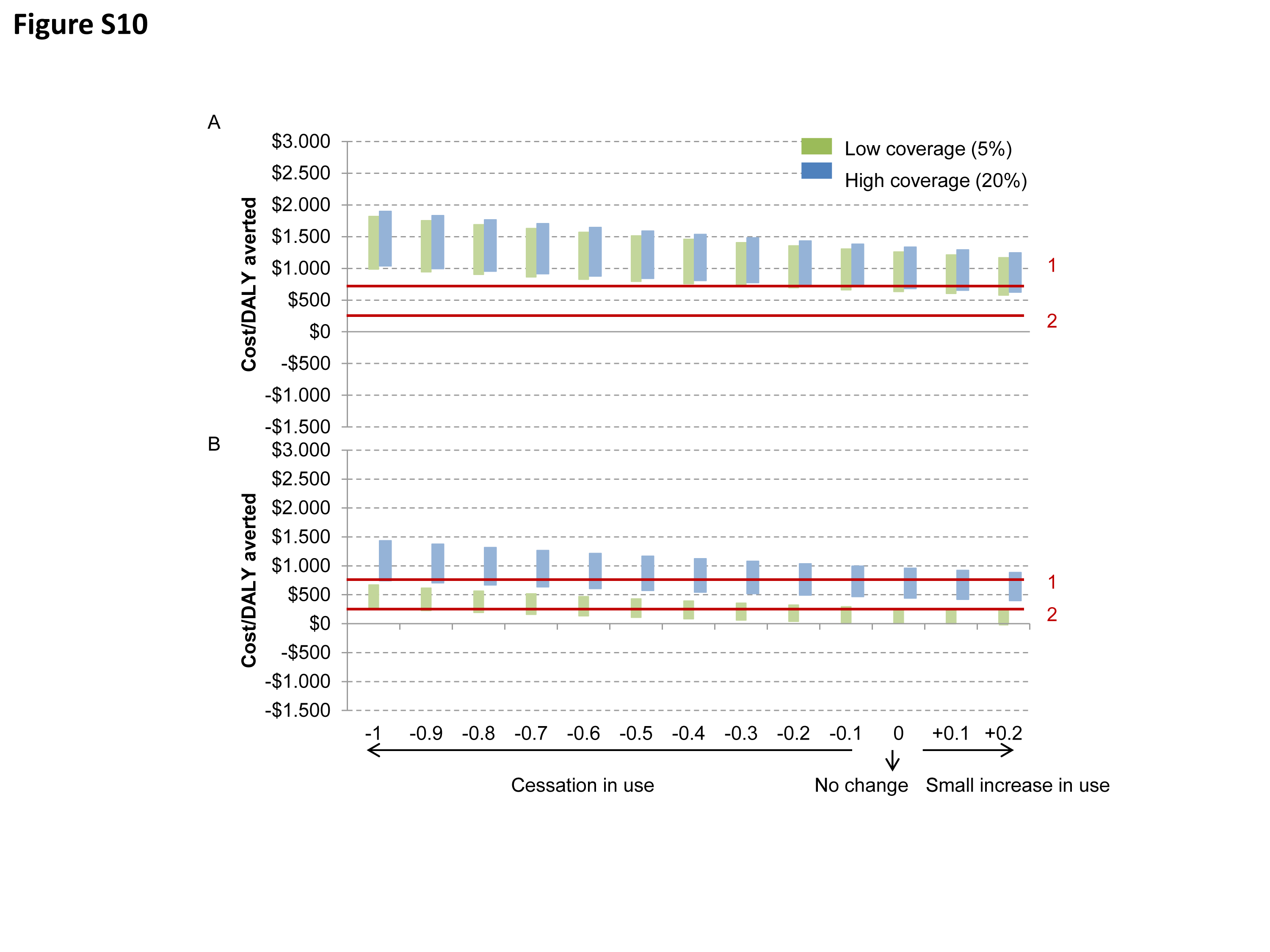

Supplement: Figure S10 — Cost-effectiveness of PrEP with respect to changes in condom use: downstream ARV costs averted included at US$1,000/person-years on ARV drugs. (A) “Uniform” scenario. (B) “Some prioritisation” scenario. This figure assumes there is no correlation between adherence and risk compensation. We explore this issue separately in Figure S12. iPrEx adherence profile used for these scenarios. In green: low coverage scenario: 5%; in blue: high coverage scenario: 20%. The red lines correspond to (1) the World Bank threshold for a cost-effective intervention, <US$745/DALY averted, and (2) the World Bank threshold for a highly cost-effective intervention, <US$149/DALY averted. (TIFF) [file pmed.1001323.s011.tif]

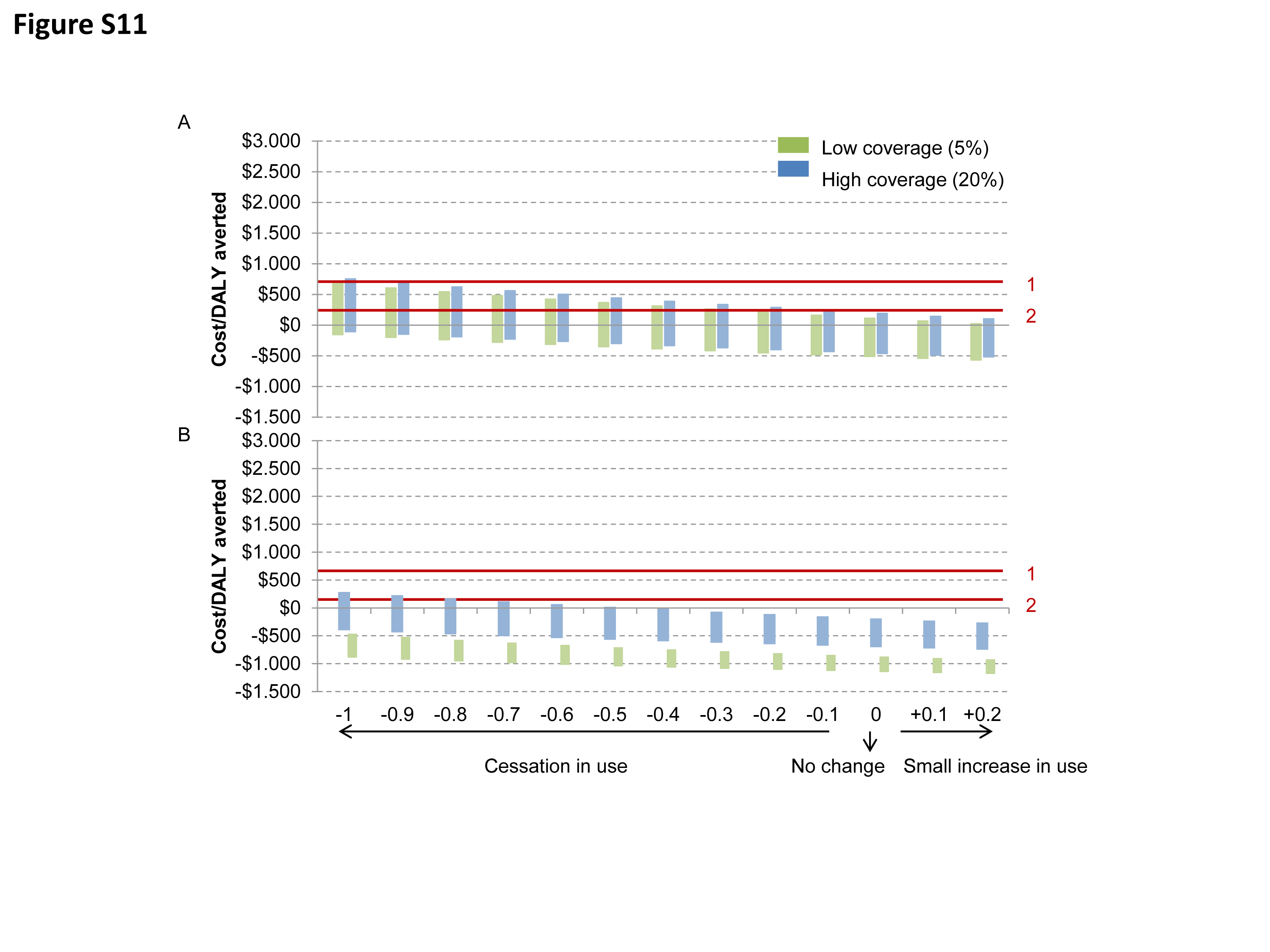

Supplement: Figure S11 — Cost-effectiveness of PrEP with respect to changes in condom use: downstream ARV costs averted included at US$3,500/person-year on ARV drugs. (A) “Uniform” scenario. (B) “Some prioritisation” scenario. This figure assumes there is no correlation between adherence and risk compensation. We explore this issue separately in Figure S12. iPrEx adherence profile used for these scenarios. In green: low coverage scenario: 5%; in blue: high coverage scenario: 20%. The red lines correspond to (1) the World Bank threshold for a cost-effective intervention, <US$745/DALY averted, and (2) the World Bank threshold for a highly cost-effective intervention, <US$149/DALY averted. (TIFF) [file pmed.1001323.s012.tif]

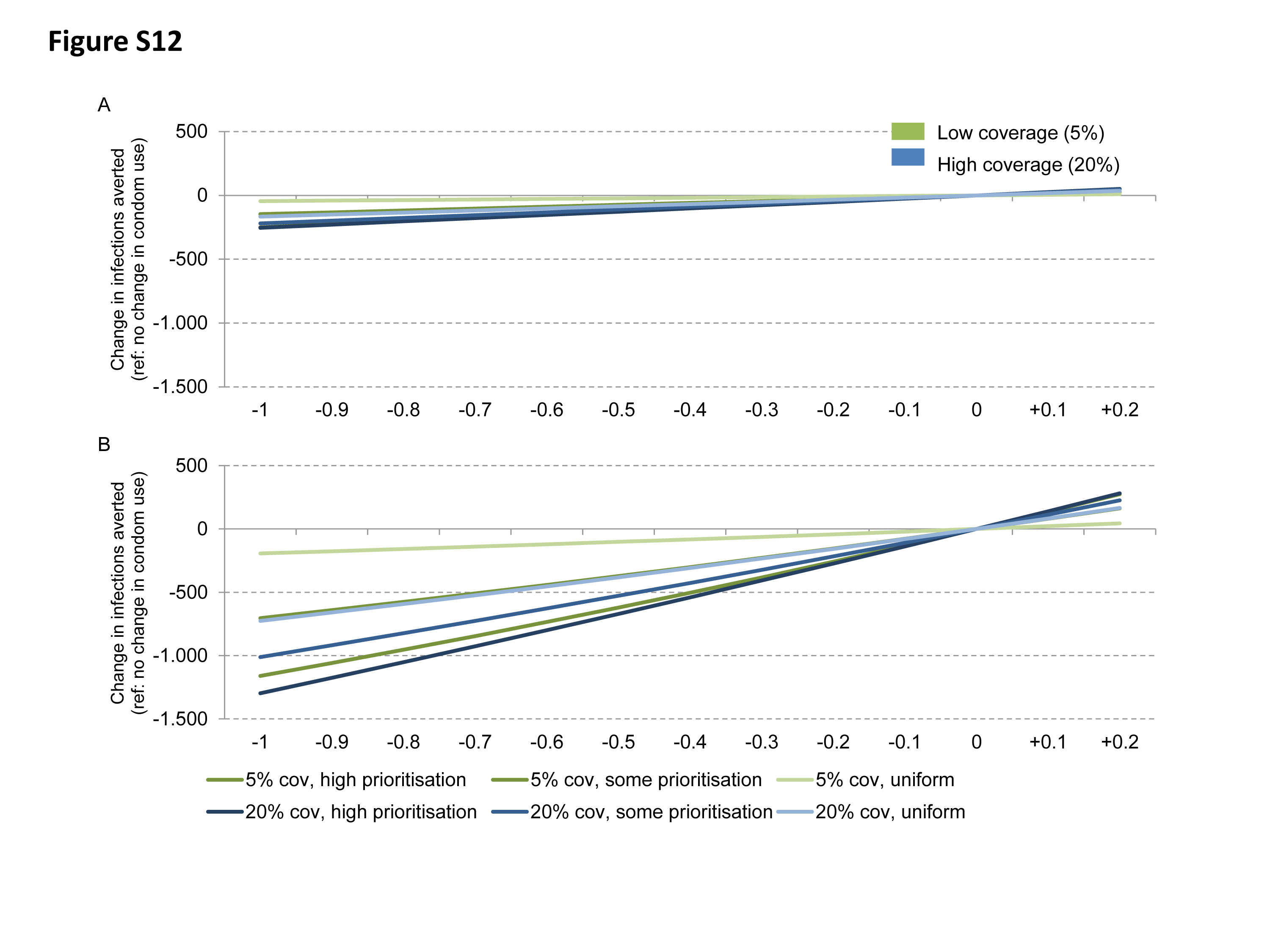

Supplement: Figure S12 — Population impact of PrEP with respect to differential changes in condom use. (A) Only good adherers change their behaviour. (B) Only bad adherers change their behaviour. This figure assumes there is a correlation between adherence and risk compensation. iPrEx adherence profile used for these scenarios. In green: low coverage scenario: 5%; in blue: high coverage scenario: 20%. Reference is no change in condom use. (TIFF) [file pmed.1001323.s013.tif]
