# Supplementary material for: The Potential Impact of Pre-Exposure Prophylaxis for HIV Prevention among Men Who Have Sex with Men and Transwomen in Lima, Peru: A Mathematical Modelling Study
Source: PLoS Med. 2012 Oct 9;9(10):e1001323. doi: 10.1371/journal.pmed.1001323 (PMC3467261; doi:10.1371/journal.pmed.1001323)
Supplement: Table S1 — Scenarios to achieve one-third of new infections averted over 10 y: downstream ARV costs averted included at US$1,000 and 3,500/person-year on ARV drugs. Total PY: total number of person-years on PrEP over 10 y. Cost/DALY: cost in US dollars/DALY. Total cost: total cost of PrEP intervention over 10 y. (1) Includes downstream ARV costs averted included at US$1,000/person-year on ARV drugs. (2) Includes downstream ARV costs averted included at US$3,500/person-year on ARV drugs. The range observed in this column and in the Total cost column represents the variation observed in the costs per PrEP person-year. Coverage in the uniform distribution strategy is equal in all subgroups (MMSW, MMSM, sex workers, and transwomen at higher risk). The coverage showed in both strategies involving prioritisation (some and high) are given as overall population coverage and, in brackets, the coverage in each subpopulation (MMSW/MMSM/sex workers/transwomen at higher risk). (DOC) [file pmed.1001323.s014.doc]

**Table S1**

| **Distribution** | **Scale up** | **Coverage** | **Total PY** | **Cost/DALY** (1) | **Total cost** (1) | **Cost/DALY** (2) | **Total cost** (2) |
| --- | --- | --- | --- | --- | --- | --- | --- |
| Uniform | Two years | 0.46 | 597,165 | [938-1,741] | [212,985,781-395,121,146] | [-207-596] | [-46,873,703-135,261,661] |
| Uniform | Five years | 0.57 | 625,155 | [1,017-1,857] | [230,871,816-421,543,582] | [-94-773] | [-20,735,759-169,936,008] |
| Some prioritisation | Two years | 0.40  (0.26/0.38/0.63/0.63) | 521,040 | [772-1,478] | [173,846,982  -332,765,071] | [-373-333] | [-83,878,936-75,039,153] |
| Some prioritisation | Five years | 0.54  (0.51/0.51/0.7/0.7) | 591,730 | [963-1,780] | [212,785,304-393,263,226] | [-182-635] | [-40,217,470-140,260,451] |
| High  prioritisation | Two years | 0.34  (0.27/0.22/0.9/0.9) | 433,810 | [568-1,155] | [127,939,027-260,250,303] | [-577-10] | [-130,069,542-2,241,734] |
| High  prioritisation | Five years | 0.49  (0.42/0.41/0.9/0.9) | 535,155 | [826-1,564] | [182,992,144-346,281,992] | [-319-419] | [-70,546,066-92,743,783] |
