# Supplementary material for: The Potential Impact of Pre-Exposure Prophylaxis for HIV Prevention among Men Who Have Sex with Men and Transwomen in Lima, Peru: A Mathematical Modelling Study
Source: PLoS Med. 2012 Oct 9;9(10):e1001323. doi: 10.1371/journal.pmed.1001323 (PMC3467261; doi:10.1371/journal.pmed.1001323)
Supplement: Text S1 — Supplementary technical information. (PDF) [file pmed.1001323.s015.pdf]

Text S1: technical supplementary information for "The potential impact of pre-exposure prophylaxis for HIV prevention among men who have sex with men and transwomen in Lima, Peru: a mathematical modelling study" GB Gomez; A Borquez; CF Caceres; ER Segura; RM Grant; GP Garnett; TB Hallett

The methods will be described in the following four parts: (1) the technical specification of the mathematical simulation model; (2) parameter values and sources used to fit the model; (3) unit cost estimation for a hypothetical PrEP intervention; and (4) calculation of disability-adjusted life years (DALYs) averted per HIV infection averted in Peruvian male adults.

1. Technical specification

We present a deterministic, compartmental model to represent the sexual transmission of HIV amongst men who have sex with other men (MSM) and transwomen in Lima, Peru. To represent HIV spread in the model, we defined four interacting groups of men: men that mostly have sex with women (MMSW), men that mostly have sex with men (MMSM), sex workers, and transwomen (including transsexuals and transvestites) at higher risk. These categories are intended to represent a broad spectrum of sexual identities, orientations, and behaviours including numbers of partners, types of partnerships formed (stable, casual, commercial), condom use, and sex work (defined as the exchange of anal sex against for money, drugs, gifts, or favours). Our subgroups definitions are presented in table 1 below.

Table 1 - Definition of groups.

**Men that mostly have sex with women (MMSW)** Men that mostly have sex with women may sporadically form commercial or casual partnerships with other men; sexual roles for MMSW were restricted to insertive and versatile (which includes both insertive and receptive anal sex acts) as the receptive role is less often reported among men who identify themselves as heterosexual.	**Men that mostly have sex with men (MMSM)** Men that mostly have sex with men represent men who generally self-identify as gay or homosexual. They might be in a stable partnership with another man, and occasionally form commercial partnerships with other men. They can be insertives, receptives or versatiles.
**Sex workers** Sex workers may have sex with men and women but their main source of risk is to report compensated sex with other men. They form commercial partnerships mainly with MMSW and MMSM, and occasionally with trans. Sex workers can be insertives, receptives or versatiles.	**Transwomen at higher risk** This group represents a high risk sub-group of the transgender population, which includes transsexuals and transvestites. They self-identify as "trans", have a large number of partners in average, higher prevalence, and a high proportion reports compensated sex. This sub-group was assumed to always play the receptive role during anal sex, as this is the role reported by the majority of trans.

The course of HIV infection was represented as distinct phases of disease progression defined by duration and infectiousness (Figure 1)[1]. When an individual gets infected, he enters a phase of acute infection (short duration, high infectiousness) and progresses to a latent phase (long duration, low infectiousness), before entering a pre-AIDS phase (short duration, high infectiousness). The disease finally progresses to an AIDS phase (short duration, no infectiousness due to an interruption of sexual activity), followed by death. A proportion of infected individuals receive antiretroviral treatment (ART) when their CD4 count is under 200 cells /mm^3, before they enter the pre-AIDS stage. ART reduces infectiousness and extends survival.

Figure 1 – Progression of HIV infection.

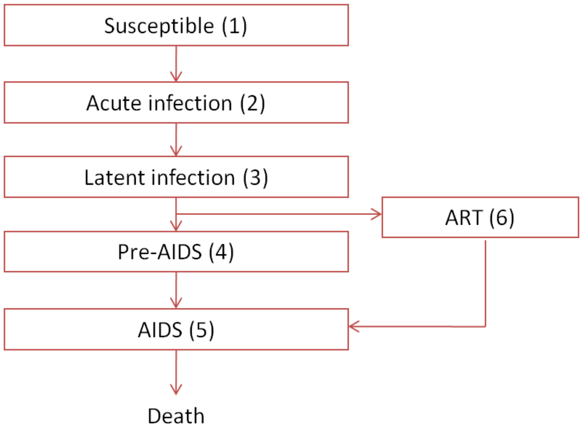

Men initially enter the model as susceptible and spend an average time period in each group. MMSM and MMSW have a slower turnover than transgender at higher risk and sex workers which reflects the transitional aspect of sex work. Once individuals exit the model, they are replaced and allocated to each group according to the initial population distribution by group. The population grows at an average rate of 2%[2]. Condom use was modelled to increase linearly from 1995 until 2005, and to remain constant at the rate reported thereafter. ART was introduced free of charge in Peru in 2004[3]. We modelled this introduction, with a small coverage starting in 2002 and a linear increase up to a 2007 coverage level of 48%[4]. It then continues to increase and stabilises at 80% coverage.

The model is defined by ordinary differential equations to simulate how HIV spreads over time. They are shown below. The state variables are given by $X^{s}_{k,a}(t)$ and $XP^{s}_{k,a}(t)$, corresponding to people who are not on PrEP and to people either on PrEP (while susceptible) or who have been on PrEP (if infected), respectively. *t* is the time elapsed in the simulation; *s* is the infection-status (1= susceptible; 2= acute infection; 3= latent infection; 4= pre-AIDS; 5= AIDS; 6= ART), *k* is 'sexual behaviour' (1= Insertive MMSW; 2=Versatile MMSW; 3=Insertive MMSM; 4=Versatile MMSM; 5=Receptive MMSM; 6=Insertive sex worker; 7=Versatile sex worker; 8=Receptive sex worker; 9= receptive transwomen at a higher risk), *a* is the PrEP adherence group (1=good; 2=average; 3=poor).

$$\frac{dX_k^1(t)}{dt} = \gamma_{k,a}\left\{(\mu+\alpha(t))\sum_{k,a}^{s}\left(X_k^s + XP_{k,a}^s\right) + \sigma_6\sum_{k}^{a}\left(X_k^6 + XP_{k,a}^6\right) + \sum_k\left(\tau_k\sum_a^s\left(X_k^s + XP_{k,a}^s\right)\right)\right\}$$
$$-\lambda_k X_k^1 - (\mu+\tau_k)X_k^1$$
$$\frac{dX_k^2(t)}{dt} = \lambda_k X_k^1 - (\sigma_2+\mu+\tau_k)X_k^2$$
$$\frac{dX_k^3(t)}{dt} = \sigma_2 X_k^2 - (\sigma_3+\mu+\tau_k)X_k^3$$
$$\frac{dX_k^4(t)}{dt} = \sigma_3 X_k^3 - (\sigma_4+\mu+\tau_k)X_k^4$$
$$\frac{dX_k^5(t)}{dt} = (1-\delta(t))\sigma_4 X_k^4 + \sigma_6 X_k^6 - (\sigma_5+\mu+\tau_k)X_k^5$$
$$\frac{dX_k^6(t)}{dt} = \delta(t)\sigma_4 X_k^4 - (\sigma_6+\mu+\tau_k)X_k^6$$

Susceptible on PrEP and infected while they wereon PrEP:

$$\frac{dXP_{k,a}^1(t)}{dt} = -\lambda P_{k,a} XP_{k,a}^1 - (\mu+\tau_k)XP_{k,a}^1$$
$$\frac{dXP_{k,a}^2(t)}{dt} = \lambda_{k,a} XP_{k,a}^1 - (\sigma_2+\mu+\tau_k)XP_{k,a}^2$$
$$\frac{dXP_{k,a}^3(t)}{dt} = \sigma_2 XP_{k,a}^2 - (\sigma_3+\mu+\tau_k)XP_{k,a}^3$$
$$\frac{dXP_{k,a}^4(t)}{dt} = \sigma_3 XP_{k,a}^3 - (\sigma_4+\mu+\tau_k)XP_{k,a}^4$$
$$\frac{dXP_{k,a}^5(t)}{dt} = (1-\delta(t))\sigma_4 XP_{k,a}^4 + \sigma_6 XP_{k,a}^6 - (\sigma_5+\mu+\tau_k)XP_{k,a}^5$$
$$\frac{dXP_{k,a}^6(t)}{dt} = \delta(t)\sigma_4 XP_{k,a}^4 - (\sigma_6+\mu+\tau_k)XP_{k,a}^6$$

Flow to PrEP

$$X_k^1 \rightarrow X_k^1 - \varsigma_{\mathrm{k}}(t)\sum_a\left(X_k^1 + XP_{k,a}^1\right) - \sum_a XP_{k,a}^1$$
$$XP_{k,a}^1 \rightarrow XP_{k,a}^1 + \upsilon_a\left(\varsigma_{\mathrm{k}}(t)\sum_a\left(X_k^1 + XP_{k,a}^1\right) - \sum_a XP_{k,a}^1\right)$$

$1/\sigma_s$ is the mean time spent in infection phase *s*; $\lambda_{k,a}$ is the force of infection for individuals of that type and behaviour; μ is the death rate, $\frac{1}{\tau_k}$ is the net mean time spent in each sexual behaviour group. $\alpha(t)$ is the population

growth rate over time; $\gamma_{k,a}$ is the proportion of people in each of the sexual behaviour and adherence groups. A certain proportion of infected individuals receive ART depending on coverage at time t ($\delta(t)$). People who exited the population due to background death, AIDS related death or population turnover are continuously replaced into the susceptible group X^1 and distributed according to $\gamma_{k,a}$. Equally, susceptible individuals who are not on PrEP continuously move to the “susceptible on PrEP” compartment ($XP^1_{k,a}(t)$) so that the PrEP coverage at that time point $\varsigma_k(t)$ is maintained. They are distributed into each adherence group according to υ_a . The boundary conditions of the system are:

$$X^1_{k,a}(0) = (1 - seed)\gamma_{k,a}N_0$$
$$X^3_{k,a}(0) = (seed)\gamma_{k,a}N_0$$

N_0 is the size of the MSM and Transgender population at the start of the simulation and $seed$ is the HIV prevalence at the start of the simulation in all parts of the population.

Force of infection

The force of infection determines the rate of progression from susceptible to infected. The force of infection through sexual contact depends on: the number of insertive and receptive partnerships C^i and C^r , respectively, the pattern of sexual partnership formation with respect to sexual behaviour (i.e. the proportion of partnerships formed with each of the sexual behaviour groups for insertive and receptive partnerships ($\rho^i_{k,k'}$ and $\rho^r_{k,k'}$ respectively), the number of sex acts occurring within that partnership ($\omega_{k,k'}$), the infection-status and stage of infection of the partner, the fraction of sex acts in which a condom is used ($\phi_{k,k'}$) and the efficacy of condoms in reducing the risk of HIV transmission (ψ). β^i_s and β^r_s are the probabilities of HIV transmission per sex act for each stage of infection for insertive and receptive anal sex respectively. For individuals using PrEP, it will also depend on their adherence to the PrEP regimen, which determines the proportion of sex acts protected by PrEP (η_a) and on the efficacy of PrEP (θ).

Off PrEP

$$\lambda_k = \sum_{k'} C^i_k \rho^i_{k,k'} \left[\frac{\sum_{s=2}^{8} (X^s_{k'} + \sum_a XP^s_{k',a})}{\sum_s \left(X^s_{k'} + \sum_a XP^s_{k',a} \right)} \left(1 - \left((1-\beta^i_s)^{\omega_{k,k'}(1-\phi_{k,k'})} (1-\beta^i_s \psi)^{\omega_{k,k'}\phi_{k,k'}} \right)\right) \right]$$

$$+ \sum_{k'} C^r_k \rho^r_{k,k'} \left[\frac{\sum_{s=2}^{8} (X^s_{k'} + \sum_a XP^s_{k',a})}{\sum_s \left(X^s_{k'} + \sum_a XP^s_{k',a} \right)} \left(1 - \left((1-\beta^r_s)^{\omega_{k,k'}(1-\phi_{k,k'})} (1-\beta^r_s \xi_{k,k'} \psi)^{\omega_{k,k'}\phi_{k,k'}} \right)\right) \right]$$

On PrEP

$$\lambda_{k,a} = \sum_{k'} C^i_k \rho^i_{k,k'} \left[\frac{\sum_{s=2}^{8} (X^s_{k'} + \sum_a XP^s_{k',a})}{\sum_s \left(X^s_{k'} + \sum_a XP^s_{k',a} \right)} \left(1 - \left(\begin{array}{l} (1-\beta^i_s)^{\omega_{k,k'}(1-\phi_{k,k'})(1-\eta_a)} (1-\beta^i_s \psi)^{\omega_{k,k'}\phi_{k,k'}(1-\eta_a)} \\ (1-\beta^i_s \theta)^{\omega_{k,k'}(1-\phi_{k,k'})\eta_a} (1-\beta^i_s \psi\theta)^{\omega_{k,k'}\phi_{k,k'}\eta_a} \end{array} \right)\right) \right]$$

$$+ \sum_{k'} C^r_k \rho^r_{k,k'} \left[\frac{\sum_{s=2}^{8} (X^s_{k'} + \sum_a XP^s_{k',a})}{\sum_s \left(X^s_{k'} + \sum_a XP^s_{k',a} \right)} \left(1 - \left(\begin{array}{l} (1-\beta^r_s)^{\omega_{k,k'}(1-\phi_{k,k'})(1-\eta_a)} (1-\beta^r_s \psi)^{\omega_{k,k'}\phi_{k,k'}(1-\eta_a)} \\ (1-\beta^r_s \theta)^{\omega_{k,k'}(1-\phi_{k,k'})\eta_a} (1-\beta^r_s \psi\theta)^{\omega_{k,k'}\phi_{k,k'}\eta_a} \end{array} \right)\right) \right]$$

The rate of sex acts in a partnership is set at three levels: (i) rate of sex acts in commercial partnerships, (ii) rate of sex acts in occasional partnerships, and (iii) rate of sex acts in stable partnerships. Table 2 describes the contact network and type of partnerships between groups. The frequency of condom use in partnerships is specific to each sexual behaviour group and is determined by the receptive partner.

Table 2. Contact patterns and type of partnerships formed between groups

			**MMSW**		**MMSM**			**Sex workers**			**Transwomen**
	Sexual Role		insertive	versatile	insertive	versatile	receptive	insertive	versatile	receptive	receptive
		k index	1	2	3	4	5	6	7	8	9
**MMSW**	insertive	1									
	versatile	2									
**MMSM**	insertive	3									
	versatile	4									
	receptive	5									
**Sex workers**	insertive	6									
	versatile	7									
	receptive	8									
**Transwomen**	receptive	9									

Note: Light pink, darker pink and very dark pink corresponds to stable, occasional and commercial partnerships respectively.

Mixing matrix

The mixing matrix designs the proportion of partnerships that are formed with individuals from each of the groups. The first step is to determine who has sex with whom. Since our groups are divided in insertive, receptive and versatile roles, there are certain combinations that cannot occur: two insertive individuals. This is illustrated in table 2. To balance the number of insertive and receptive partnerships we first defined a mixing matrix for the insertive individuals, reflecting their preference for certain types of partners, as well as a random mixing matrix where insertive individuals chose their partner according to the proportion of sex acts offered by each of the receptive groups - this assumes that the amount of sex the “providers” have is totally dependent of the demand. The extent to which the mixing was closer to satisfy the preferences of insertive individuals was determined by a parameter ε varying from one (totally assortative mixing) to zero (totally random mixing). The total number of partners among the receptive groups was then recalculated to respond to the demand and the mixing matrix for receptive individuals was updated.

$$\rho^{i}_{k,k'} = \varepsilon A_{k,k'} + (1-\varepsilon) B_{k,k'}$$

$$B_{k,k'} = \frac{C^{i}_{k'}\left(\sum_{s} X^{s}_{k',1} + \sum_{a,s} X^{s}_{k',a}\right)}{\sum_{k'} C^{r}_{k'}\left(\sum_{s} X^{s}_{k',1} + \sum_{a,s} X^{s}_{k',a}\right)}$$

$$C^{r}_{k}{}' = \frac{C^{i}_{k'}\rho^{i}_{k',k}\left(\sum_{s} X^{s}_{k'} + \sum_{a,s} X^{s}_{k',a}\right)}{\sum_{s} X^{s}_{k} + \sum_{a,s} X^{s}_{k,a}}$$

$$\rho^{r}_{k,k'} = \frac{C^{i}_{k'}\rho^{i}_{k,k'}\left(\sum_{s} X^{s}_{k'} + \sum_{a,s} X^{s}_{k',a}\right)}{C^{r}_{k}{}'\left(\sum_{s} X^{s}_{k} + \sum_{a,s} X^{s}_{k,a}\right)}$$

$\rho^{i}_{k,k'}$ and $\rho^{r}_{k,k'}$ define the proportion of total partnerships in group k that are formed with group k’ for insertive and receptive partnerships respectively. $A_{k,k'}$ is the expected proportion of partnerships that insertive individuals in group k will have with receptive individuals in group k’ and $B_{k,k'}$ is the proportion of receptive partnerships given by receptive individuals in group k’ out of the total number of partnerships C^{r} given by all receptive individuals in all groups. ε determines the extent to which the mixing is in accordance to the insertive partners preferences or dependent on availability. $C^{r}_{k}{}'$ is the updated number of receptive partnerships individuals in group k give in order to respond to the demand.

2. **Model fit: parameter values and sources**

Bayesian melding

The epidemic was simulated with 10,000 different parameter sets. A run was accepted if it fitted within the prior limits corresponding to two prevalence values in each of the groups. These two data points were chosen giving preference to large sample size studies: one closest to the beginning of the epidemic and one closer to date (coloured in blue in Table 3). To produce lower and higher bounds we arbitrarily subtracted and added 0.2 respectively. If the lower bound was negative it was replaced by zero. The most discriminative prior was the high prevalence observed among transwomen relatively early in the epidemic. The parameters allowed to vary were those describing sexual behaviour as well as a couple describing the natural history of infection. Descriptive statistics for their prior and posterior distributions are given in tables 4 and 5 when applicable. The parameters for which the posterior distributions diverged the most from the prior distribution were the basic transmission probability (towards higher values) and the year at which the epidemic started (towards earlier start) as well as the sex workers turnover (towards slower), the number of sex acts in a commercial partnership (towards higher values) and the number of sex acts in a stable partnership (towards lower values). In general, the selection process favoured parameter values that increased risk among sex workers and transwomen and decreased risk among MMSW.

Table 3 – Prevalence by group

Population	year	Sample size	Prevalence	Reference
All MSM	1985	98	0·112	[5]
All MSM	1988	124	0·065	[6]
All MSM	1990	4300	0·262	[7]
All MSM	1996	444	0·185	[8]
All MSM	1997	1328	0·16	[9]
All MSM	1998	4858	0·122	[10]
All MSM	1998	1211	0·178	[8]
All MSM	1998	469	0·182	[9]
All MSM	2000	3200	0·165	[9]
All MSM	2000	1357	0·197	[8]
All MSM	2000	7041	0·139	[11]
All MSM	2002	1358	0·223	[8]
All MSM	2007	559	0·222	[12]
All MSM	2008	225	0·093	[13]
All MSM	2008	170	0·117	[13]
All MSM	2008	390	0·21	[13]
All MSM	2008	318	0·179	[14]
MMSW	1996	129	0·139	[8,15]
MMSW	1998	263	0·091	[8]
MMSW	2000	533	0·084	[8]
MMSW	2002	511	0·129	[8]
MMSW	2002	165	0·025	[16]
MMSW	2002	1124	0·073	[17]
MMSW	2008	109	0·055	[13]
MMSW	2008	21	0·29	[14]
MMSM	1996	265	0·18	[8,15]
MMSM	1998	796	0·181	[8]
MMSM	2000	661	0·26	[8]
MMSM	2002	562	0·262	[8]
MMSM	2002	1760	0·195	[17]
MMSM	2008	253	0·186	[14]

Transwomen	1996	48	0·333	[8,15]
Transwomen	1998	134	0·343	[8]
Transwomen	2000	96	0·448	[8]
Transwomen	2002	255	0·322	[8]
Transwomen	2008	208	0·178	[14]
Transwomen	2009	459	0·296	[18]
Sex workers	2008	169	0·243	[13]
Sex workers	2008	181	0·265	[13]
Sex workers	2008	183	0·284	[13]
Sex workers	2008	391	0·207	[14]

Table 4 – Incidence by group (infections/100 person years)

Population	year	Incidence	Reference
High risk MSM	1998-2000	3·5 [2·3-5·0]	[19]
All MSM	2002-2006	2·2 [1·3-3·6]	[20]

Table 5 – Distribution of risk in the population and risk behaviours

Parameter	Prior Value Mode [min-max]	Prior Value Mean [variance]	Posterior Value Mean [variance]	Reference
Proportion of all MSM: MMSW	0·2 [0·08-0·3]	0.19[0.002]	0.18 [0.002]	[8,17,19,21,22]
Proportion of all MSM: sex worker	0·15 [0·1-0·25]	0.17[0.001]	0.17 [0.001]	[8,12,19,22,23,24]
Proportion of all MSM: Transwomen	0·05 [0·043-0·1]	0.06[0.0002]	0.06[0.0001]	[22]
Mean duration: MMSW	20-30	24 [8.3]	24 [8.0]	Assumption
Mean duration: MMSM	25-35	29[8.3]	29[8.1]	Assumption
Mean duration: sex worker	2-10	3[5]	4[4.5]	Assumption
Mean duration: Transwomen	10-20	13[8.3]	13[7.6]	Assumption
N sex acts per commercial partnership	1-2	1.5[0.08]	1.7[0.06]	Assumption
N sex acts per stable partnership	40-80	60[133]	58 [132]	[25]
N sex acts per casual partnership	3-15	9[12]	9.6 [12]	[26]
Condom change	1.5-2	1.75[0.02]	1.76[0.02]	[8,14,18,22]
Epsi (mixing matrix)	0.6[0.4-0.99]	0-66[0.015]	0.64[0.013]	Assumption
Sexual behaviour: MMSW				
Pr of all MMSW: insertive	0·85 [0·8-0·9]	0.85[0.0004]	0.85[0.0005]	[27]
N partnerships/year: MMSW insertive	2[1-4]	2.3[0.39]	2.4[0.42]	[14,15]
Pr of protected sex acts: MMSW insertive	0·3-0·35	0.33[0.0002]	0.33[0.0002]	[15,26,27]
N partnerships/year: MMSW versatile	3·5 [1.5-4]	3[0.29]	3 [0.29]	[14,15,26,27]
Pr of protected sex acts: MMSW versatile	0·3-0·45	0.38[0.002]	0.38[0.002]	[15,26]
Sexual behaviour: MMSM				
Pr of all MMSM: insertive	0·25 [0·2-0·3]	0.25[0.0004]	0.25[0.0005]	[12,17]
N partnerships/year: MMSM insertive	1·2 [1-3]	1.7[0.20]	1.7[0.18]	[14,28]
Pr of protected sex acts: MMSM insertive	0·2-0·35	0.28[0.002]	0.28[0.002]	[15]
Pr of all MMSM: receptive	0·325 [0·3-0·35]	0.33[0.0001]	0.32[0.0001]	[12,17]
N partnerships/year: MMSM receptive	3[1-4]	2.7[0.4]	2.7[0.4]	[14,15]
Pr of protected sex acts: MMSM receptive	0·3-0·45	0.38[0.002]	0.38[0.002]	[15]

N partnerships/year: MMSM versatile	3.5 [1.5-4]	3[0.29]	3[0.2808]	[14,15]
Pr of protected sex acts: MMSM versatile	0·25-0·4	0.32[0.002]	0.33[0.002]	[15]
Sexual behaviour: sex worker				
Pr of all sex worker: insertive	0·2 [0·15-0·25]	0.2[0.0004]	0.2[0.0004]	[28]
N partnerships/year: sex worker insertive	30 [10-35]	25[29]	27[27]	[28]
Pr of protected sex acts: sex worker insertive	0·05-0·2	0.13[0.002]	0.13[0.002]	[28]
Pr of all sex worker: receptive	0·4 [0·35-0·5]	0.42[0.001]	0.42[0.001]	[28]
N partnerships/year: sex worker receptive	30 [10-50]	30[67]	30[69]	[28]
Pr of protected sex acts: sex worker receptive	0·2-0·3	0.25[0.001]	0.25[0.001]	[28]
N partnerships/year: sex worker versatile	50 [30-60]	47[39]	48[37]	[28]
Pr of protected sex acts: sex worker versatile	0·1-0·25	0.18[0.002]	0.17[0.002]	[28]
Sexual behaviour: transwomen				
N partnerships/year: transwomen	80 [50-150]	93[439]	96[492]	[14,26]
Pr of protected sex acts: transwomen	0·35-0·45	0.4[0.001]	0.40[0.001]	[15]

Note: N: number; Pr: proportion; min: minimum; max: maximum; ref: reference.

The parameters that determine the distribution of the population (i.e. the proportion of the total population represented by each group) are interdependent as they must add up to one. These were determined using a tree (Figure 2).

Figure 2 – Calculation of the proportion of people in each group

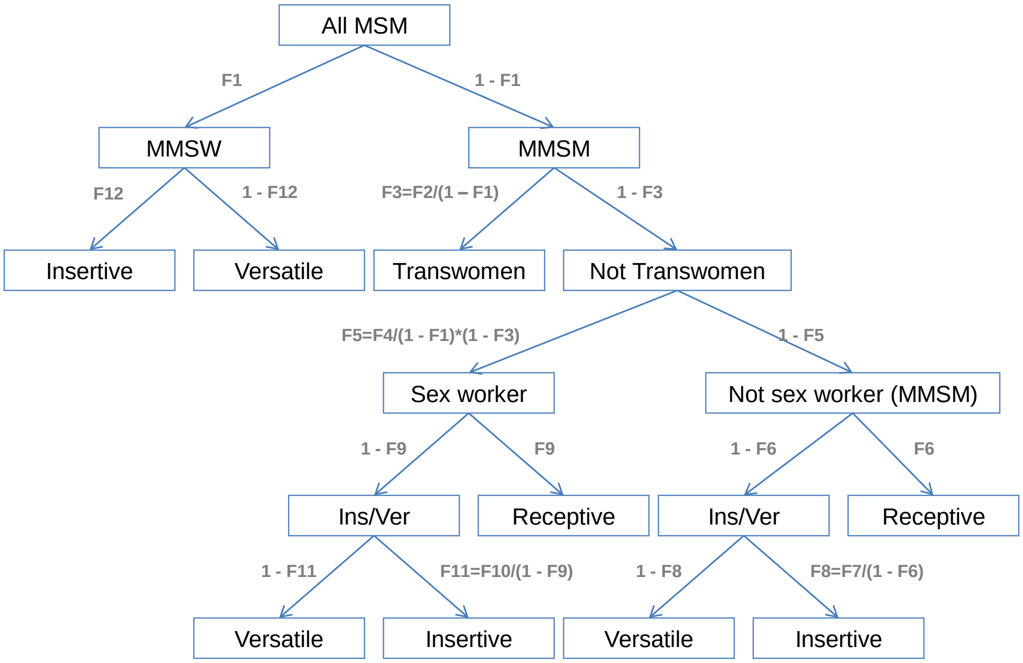

Note: MMSW – men that mostly have sex with women; MMSM – men that mostly have sex with men; Ins – insertive; Ver – versatile.

Parameters that define the natural history of HIV (Table 6) were estimated from the general scientific literature and generalised across all settings. For reasons of computational efficiency, the uncertainty in these parameter values is not reflected in the estimates of uncertainty, with the exception of β^{2-3} which corresponds to the baseline HIV transmission probability during an insertive anal sex act in the latent phase of infection. Parameters that describe the basic demography of the population (Table 7) are specific to each subgroup but are not used in the model 'fitting' procedure.

Table 6 – Natural history of HIV

Parameters	Description	Symbol	Prior Value mode [min-max]	Prior Value mean[variance]	Posterior Value mean[variance]	Reference
Average transmission rate of HIV per sex act	if latent HIV infection	β^{3-4}	0·0028[0·002-0·004]	0.0026[3.8 10-7]	0.003[1.6 10-7]	[29]
Start of the epidemic	year		[1970-1982]	1976[12]	1974.5[10.4]	[30]
Relative transmission rate per insertive anal sex act			1			Def.
Relative transmission rate per receptive anal sex act	(ref. insertive anal sex)	ξ	5			[31,32]
Relative infectiousness in acute phase infection	(ref. latent infection)	β^{2}	27			[1]
Relative infectiousness in latent phase infection		β^{3-4}	1			Def.
Relative infectiousness in pre-AIDS phase infection	(ref. latent infection)	β^{5}	7·2			[1]
Relative infectiousness in AIDS phase infection	(ref. latent infection)	β^{6}	0			[1]
Relative infectiousness of virally-suppressed individuals on ART	(ref. latent infection)	β^{7-8}	0·08			[1,33]
Mean duration of acute phase infection	months	$1/\sigma_2$	3			[1,33]
Mean duration of latent phase	years	$1/\sigma_3$	10			[1,33]
Mean interval with elevated viral load, pre-AIDS	months	$1/\sigma_4$	10			[1,33]
Mean interval with AIDS before death	months	$1/\sigma_5$	9			[1,33]
Mean duration of late ART viral suppression	years	$1/\sigma_6$	12			[33,34]
Mean ART coverage of those in need (CD4<200)	2007	ART_cov	0·48			[4,35]
Efficacy of condoms		Ψ	0.8			[36,37]

Table 7 – Basic demography of the population

Parameters	Description	Value	Reference
Population 15 to 49y	2007	4,767,148	[2]
Population 15 to 49y	1981	2,503,140	[38]
First report of AIDS cases in Peru		1983	[30]
Proportion of the population that are men in Lima, Peru	2007	0·489	[2]
Proportion of male-to-male sex in general population		0·06	[16,21,39]

Figure 3 – Best fit and prevalence data for four sub-groups and the overall population.

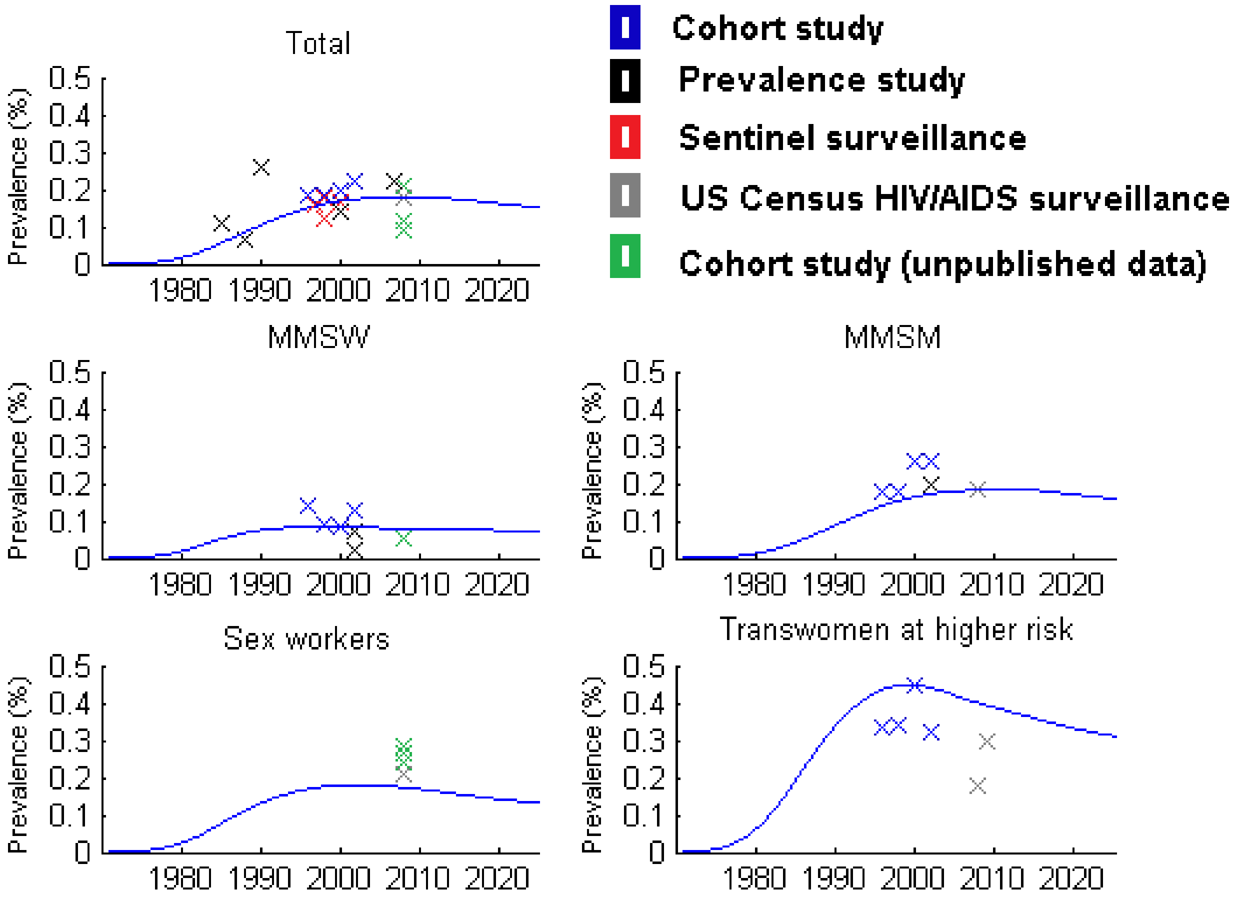

We explored the degree of uncertainty associated with the epidemiological assumptions. We calculated the number of infections averted for a low coverage (5%) scenario under all prioritisation strategies, using the iPrEX adherence profile and 92% efficacy assumption for each of the selected parameter sets. This corresponded to 449 runs as we applied additional filters to the 708 selected runs to ensure that the proportion of the population in each sexual behaviour group corresponded to what had been observed in recent studies, as this criterion was used together with the log-likelihood estimation to select our best fit. The resulting distributions for the 449 selected runs and for the best 50 fits according to log-likelihood are shown in figure 4. The number of infections averted used in the analysis for each scenario corresponds to the red lines.. In general, we see that although the range of infections averted is broad (from 758 to 1714, 1102 to 5085 and 915 to 5460 for the uniform, some prioritisation and high prioritisation scenario respectively), the distributions broadly follow a normal shape meaning that most results concentrate around the mean. The fit chosen falls close to both the median for all selected runs and for the 50 best fits for the some and high prioritisation scenarios. It falls close to the lower bound of the distribution for the uniform prioritisation scenario suggesting we might be under-estimating the potential impact of this strategy. However, the main result remains unchanged with more

infections being averted when the intervention is prioritised to MSW and transwomen and with the scenarios we explored broadly meeting WHO criteria for cost-effectiveness.

Figure 4 - Uncertainty around the number of infections averted for a ten year intervention for all coverage scenarios and all prioritisation strategies

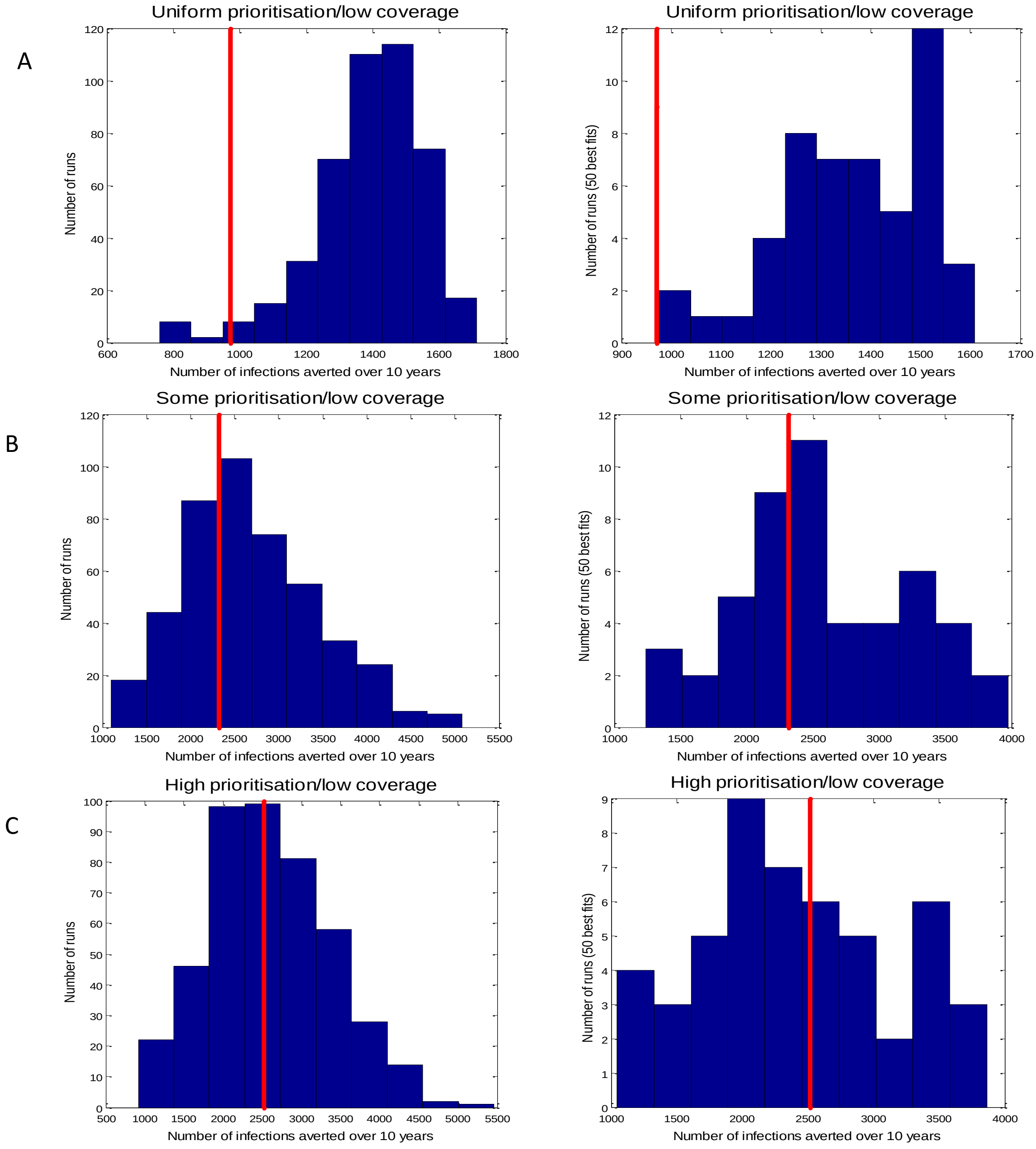

D

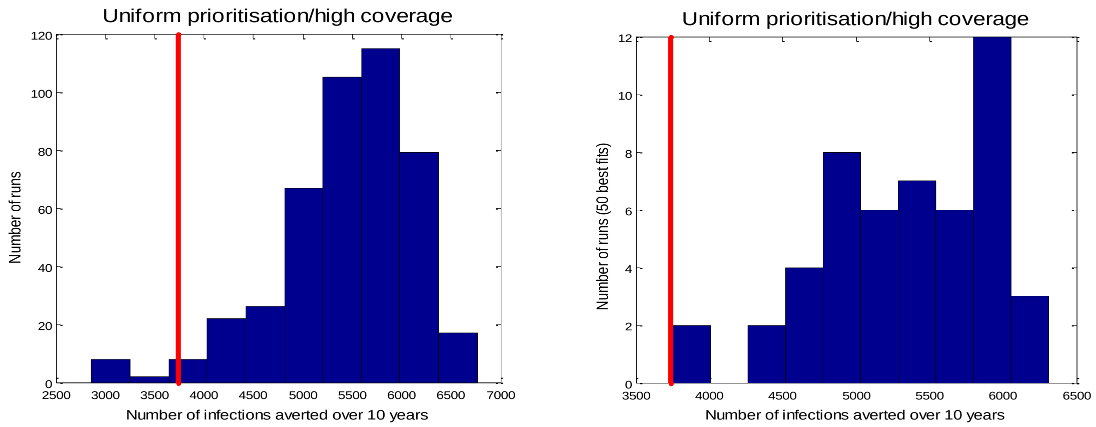

E

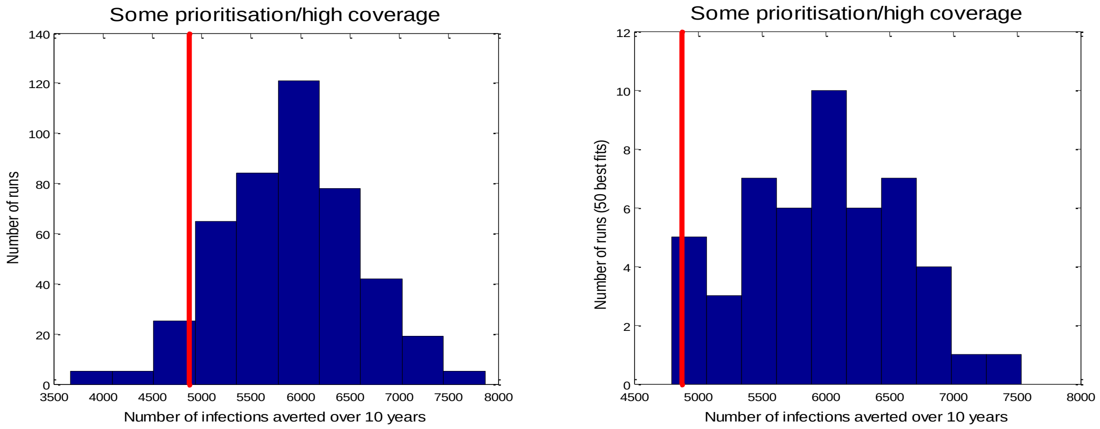

F

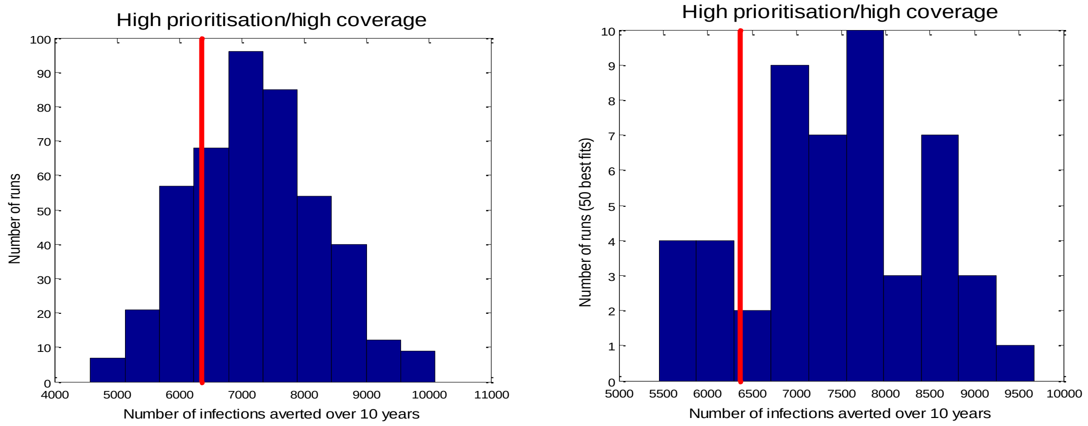

Note: A: uniform prioritisation/low coverage; B: some prioritisation/low coverage; C: high prioritisation scenarios/low coverage; D: uniform prioritisation/high coverage; E: some prioritisation/high coverage; F: high prioritisation scenarios/high coverage. Red lines: best fit.

Finally, we investigated the potential implications of restricting our analyses to the 10 years of the intervention in terms of the number infections averted. Since it is possible that those who did not get infected while they were taking PrEP, get infected subsequently, we ran the analysis for an extra 60 years after the end of the intervention (corresponding to the life expectancy in the model). We found that the number of infections averted is greater when looking further into the future, suggesting averting infections during the 10 years of the intervention results in averting secondary infections later on. Results for the low coverage, all prioritisation strategies, under the IPrEX adherence scenario and assuming 92% efficacy are shown in table 8.

Table 8 – Number of infections averted for the low coverage (5%) scenario under each prioritisation strategy when restricting the analysis to the intervention period and when projecting 60 years into the future.

		Number of infections averted	
		10 year intervention	10 year intervention + 60 years
Prioritisation scenario	Uniform	971	1965
	Some	2317	4560
	High	2519	5115

3. **Unit cost estimation for a hypothetical PrEP intervention**

Table 10 – Estimated cost of a hypothetical PrEP intervention

**Group**	**Trans**	**Sex worker**	**MMSM**	**MMSW**
Number of people on PrEP	1,000	1,000	1,000	1,000
**1. Behaviour change communication (including outreach and peer-outreach activities)**				
a. Peer education				
Peer educator to person on PrEP ratio (UNAIDS recommended ratio 1:10 to 1:20)[40]	15	15	20	20
(Total number of peer educators)	66·6	66·6	50	50
Cost peer educator/day[41]	16·5	16·5	16·5	16·5
Number of days of work/week (peer educator)[40]	2	2	2	2
b. Peer educator training/support (outreach workers)				
Number of trainers required per training course[40,41]	2	2	2	2
Number of days in one course[40,41]	2	2	2	2
Number of peer educators attending each course[40,41]	20	20	20	20
Daily allowance to attend course/participant[41]	16·5	16·5	16·5	16·5
Trainer fees/day[41]	33	33	33	33
Cost of materials/participant/course[41]	5·5	5·5	5·5	5·5
Cost of food and refreshment/participant/course[41]	11	11	11	11
Outreach workers to peer educator ratio[40,41]	17	17	17	17
Monthly salary: outreach worker[41]	330	330	330	330
**2. Delivery of tools, services for prevention, and care**				
a. Provision of condoms and lubricant				
Average number of partners/year (per group)	100	30	2·5	2·5
Average number of sex acts/partner (per group)	2	4	45	4
Proportion of condoms used consistently (per group)	0·68	0·34	0·595	0·595
Proportion of condoms covered by the programme	0·4	0·4	0·4	0·4
Cost/male condom and lubricant[41]	0·022	0·022	0·022	0·022
b. Services for prevention (PrEP, counselling and testing)				
Number of doctors/PrEP unit	1	1	1	1
Number of nurses/PrEP unit	2	2	2	2
Number of counsellors/PrEP unit	3	3	3	3
Monthly salary: doctor[41,42]	1,300	1,300	1,300	1,300
Monthly salary: nurse[41,42]	850	850	850	850
Monthly salary: counsellor[41,42]	650	650	650	650
Cost of ARV for PrEP/year (*estimated $35 or 50/month/person[43])	420/600	420/600	420/600	420/600
Average number of HIV tests/year/person[44]	4	4	4	4
Cost of single HIV test[41]	2·23	2·23	2·23	2·23
Prevalence HIV (estimate for each group)	0·4	0·3	0·2	0·05
Cost confirmation HIV test[41]	9·8	9·8	9·8	9·8
Cost of creatinine/BUN tests[45,46]	7·7	7·7	7·7	7·7
Number of tests/year[44]	1	1	1	1
Unit cost: management/M&E (programme excl. outreach)**	25.1/34.1	25.0/34.0	24.9/33.9	24.8/33.8
Unit cost: management/M&E (programme incl. outreach)**	30.4/39.4	30.3/39.3	29.0/38.0	28.9/37.9
Total (cost/person-year on PrEP – excl. outreach)**	526.2/715.2	524.3/713.3	523.5/712.5	521.4/710.4
Total (cost/person-year on PrEP – incl. outreach)**	638.7/827.7	636.8/825.8	608.0/797.0	605.9/794.9

Notes: all costs in US$. *Estimated discounted cost in discussion should Truvada (tenofovir disoproxil fumarate [TDF: 300 mg] plus emtricitabine [FTC: 200 mg]) be approved for prevention use in Peru.**unit cost of programme if PrEP drugs cost $35 or $50.

4. Calculation of DALYs averted per HIV infection averted

Table 11 – Summary comparison of DALYs averted per HIV infection averted in Peruvian male adults varying assumptions of age-weighting and discounting [47,48,49,50]

	**Assumptions: [0.03,1,0.04]**	**Assumptions: [0.03,0,0]**	**Assumptions: [0,1,0.04]**	**Assumptions: [0,0,0]**
DALYs averted (ART)	18.86	16.53	38.01	35.63
DALYs averted (no ART)	10.65	10.19	24.36	24.99
DALYs averted (80% ART coverage)	12.29	11.46	27.09	27.12

Assumptions are presented as [r,K,β]; where r is the discount rate (0.03 or 0), K is the age weighting modulation factor (1 or 0), β is the parameter from the age weighting function (0.04 or 0).

Table 12 – Detailed calculation of DALYs averted per HIV infection averted in Peruvian male adults [47,48,49,50]

Variables	symbol	no ART	ART	Variables	symbol	Pre AIDS (no ART)	Pre AIDS (ART)	AIDS (no ART)	AIDS (ART)
a. Assumptions: [0.03,1,0.04] – with discounting and age weighting									
Age weighting modulation factor	K	1	1			1	1	1	1
Age weighting constant	C	0.1658	0.1658			0.1658	0.1658	0.1658	0.1658
Discount rate	R	0.03	0.03			0.03	0.03	0.03	0.03
Constant from the age weighting function	B	0.04	0.04			0.04	0.04	0.04	0.04
	E	2.72	2.72		e	2.72	2.72	2.72	2.72
Age at death	A	36.75	48.75	Age at onset of disability	a	24.92	24.92	36.00	48.00
Standard expectation of life at age a	L	33.75	21.75	Duration of disability	L	11.08	23.08	0.75	0.75
				Disability weight	D	0.135	0.135	0.505	0.167
YLL (at age 36.75 or 48.75)		23.44	15.01	YLDs		1.89	3.17	0.53	0.14
Discount rate	R	0.03	0.03						
YLL (at age 24.92)		16.44	7.34						
b. Assumptions: [0.03,0,0] – with discounting and no age weighting									
Age weighting modulation factor	K	0	0		K	0	0	0	0
Age weighting constant	C	0.1658	0.1658		C	0.1658	0.1658	0.1658	0.1658
Discount rate	R	0.03	0.03		r	0.03	0.03	0.03	0.03
Constant from the age weighting function	B	0	0		β	0	0	0	0

	E	2.72	2.72		e	2.72	2.72	2.72	2.72
Age at death	A	36.75	48.75	Age at onset of disability	a	24.92	24.92	36.00	48.00
Standard expectation of life at age a	L	33.75	21.75	Duration of disability	L	11.08	23.08	0.75	0.75
				Disability weight	D	0.135	0.135	0.505	0.167
YLL (at age 36.75 or 48.75)		21.22	15.98	YLDs		1.27	2.25	0.37	0.12
Discount rate	R	0.03	0.03						
YLL (at age 24.92)		14.88	7.81						
c. Assumptions: [0,1,0.04] – with no discounting and age weighting									
Age weighting modulation factor	K	1	1		K	1	1	1	1
Age weighting constant	C	0.1658	0.1658		C	0.1658	0.1658	0.1658	0.1658
Discount rate	R	0	0		r	0	0	0	0
Constant from the age weighting function	B	0.04	0.04		β	0.04	0.04	0.04	0.04
	E	2.72	2.72		e	2.72	2.72	2.72	2.72
Age at death	A	36.75	48.75	Age at onset of disability	a	24.92	24.92	36.00	48.00
Standard expectation of life at age a	L	33.75	21.75	Duration of disability	L	11.08	23.08	0.75	0.75
				Disability weight	D	0.135	0.135	0.505	0.167
YLL (at age 36.75 or 48.75)		35.26	19.90	YLDs		2.22	4.32	0.53	0.15
d. Assumptions: [0,0,0] – with no discounting and no age weighting									
Age weighting modulation factor	K	0	0		K	0	0	0	0
Age weighting constant	C	0.1658	0.1658		C	0.1658	0.1658	0.1658	0.1658
Discount rate	R	0	0		r	0	0	0	0
Constant from the age weighting function	B	0	0		β	0	0	0	0
	E	2.72	2.72		e	2.72	2.72	2.72	2.72
Age at death	A	36.75	48.75	Age at onset of disability	a	24.92	24.92	36.00	48.00
Standard expectation of life at age a	L	33.75	21.75	Duration of disability	L	11.08	23.08	0.75	0.75
				Disability weight	D	0.135	0.135	0.505	0.167
YLL (at age 36.75 or 48.75)		33.75	21.75	YLDs		1.50	3.12	0.38	0.13

YLLs: years of life lost; YLDs: years of life lived with disability.

References

1. Hollingsworth TD, Anderson RM, Fraser C (2008) HIV-1 transmission, by stage of infection. J Infect Dis 198: 687-693.
2. INEI (2007) Censo de la poblacion. Peru. http://censos.inei.gob.pe/censos2007/ (accessed November 2011)
3. Ley Nº 28243: Ley que amplia y modifica la Ley 266246 sobre el virus de inmunodeficiencia humana (VIH), el sindrome de inmunodeficiencia adquirida (SIDA) y las infecciones de transmision sexual (May 2004). http://www.congreso.gob.pe/ntley/Imagenes/Leyes/28243.pdf (accessed November 2011)
4. UNAIDS (2008 update) Peru: Epidemiological Fact Sheet on HIV and AIDS: Core data on epidemiology and response Geneva. http://apps.who.int/globalatlas/predefinedReports/EFS2008/full/EFS2008_PE.pdf (accessed November 2011)
5. Rojas G, Gotuzzo E, Yi A, Koster F (1986) Acquired immunodeficiency syndrome in Peru. Ann Intern Med 105: 465-466.
6. Caceres C, Gotuzzo E, Wignall S, Campos M (1991) Sexual behavior and frequency of antibodies to type 1 human immunodeficiency virus (HIV-1) in a group of Peruvian male homosexuals. Bull Pan Am Health Organ 25: 306-319.
7. McCarthy MC, Wignall FS, Sanchez J, Gotuzzo E, Alarcon J, et al. (1996) The epidemiology of HIV-1 infection in Peru, 1986-1990. AIDS 10: 1141-1145.
8. Sanchez J, Lama JR, Kusunoki L, Manrique H, Goicochea P, et al. (2007) HIV-1, sexually transmitted infections, and sexual behavior trends among men who have sex with men in Lima, Peru. J Acquir Immune Defic Syndr 44: 578-585.
9. Calleja JM, Walker N, Cuchi P, Lazzari S, Ghys PD, et al. (2002) Status of the HIV/AIDS epidemic and methods to monitor it in the Latin America and Caribbean region. AIDS 16 Suppl 3: S3-12.
10. Jorge S, Russell K, Carcamo C (2000) Abs: ThOrC717: HIV Sentinel Surveillance for Men Who have Sex with Men in Peru. XIII International AIDS Conference. Durban, South Africa.
11. Montano SM, Sanchez JL, Laguna-Torres A, Cuchi P, Avila MM, et al. (2005) Prevalences, genotypes, and risk factors for HIV transmission in South America. J Acquir Immune Defic Syndr 40: 57-64.
12. Clark JL, Konda KA, Segura ER, Salvatierra HJ, Leon SR, et al. (2008) Risk factors for the spread of HIV and other sexually transmitted infections among men who have sex with men infected with HIV in Lima, Peru. Sex Transm Infect 84: 449-454.
13. HIV/AIDS Surveillance - US Census Bureau. http://hivaidssurveillancedb.org/ (accessed November 2011)
14. Caceres C, Segura ER (2011) Unpublished data: CPOS study - baseline data.
15. Tabet S, Sanchez J, Lama J, Goicochea P, Campos P, et al. (2002) HIV, syphilis and heterosexual bridging among Peruvian men who have sex with men. AIDS 16: 1271-1277.
16. Garcia P, Holmes KK, Garnett GP (2006) The PREVEN Project: Urban community randomized trial for prevention of STI in Peru. Peru.
17. Lama JR, Lucchetti A, Suarez L, Laguna-Torres VA, Guanira JV, et al. (2006) Association of herpes simplex virus type 2 infection and syphilis with human immunodeficiency virus infection among men who have sex with men in Peru. J Infect Dis 194: 1459-1466.
18. Caceres C, Segura ER (2011) Unpublished data: Trans-Amfar study - baseline data.
19. Sanchez J, Lama JR, Peinado J, Paredes A, Lucchetti A, et al. (2009) High HIV and ulcerative sexually transmitted infection incidence estimates among men who have sex with men in Peru: awaiting for an effective preventive intervention. J Acquir Immune Defic Syndr 51 Suppl 1: S47-51.
20. Caceres C, Segura ER (2011) Unpublished data: CPOL study - incidence data.
21. Caceres C, Konda K, Pecheny M, Chatterjee A, Lyerla R (2006) Estimating the number of men who have sex with men in low and middle income countries. Sex Transm Infect 82 Suppl 3: iii3-9.

22. Caceres CF, Konda K, Segura ER, Lyerla R (2008) Epidemiology of male same-sex behaviour and associated sexual health indicators in low- and middle-income countries: 2003-2007 estimates. Sex Transm Infect 84 Suppl 1: i49-i56.
23. Caceres CF, Konda KA, Salazar X, Leon SR, Klausner JD, et al. (2008) New populations at high risk of HIV/STIs in low-income, urban coastal Peru. AIDS Behav 12: 544-551.
24. Clark JL, Caceres CF, Lescano AG, Konda KA, Leon SR, et al. (2007) Prevalence of same-sex sexual behavior and associated characteristics among low-income urban males in Peru. PLoS One 2: e778.
25. Stover J, Bertrand JT, Shelton JD (2000) Empirically based conversion factors for calculating couple-years of protection. Eval Rev 24: 3-46.
26. Konda KA, Lescano AG, Leontsini E, Fernandez P, Klausner JD, et al. (2008) High rates of sex with men among high-risk, heterosexually-identified men in low-income, coastal Peru. AIDS Behav 12: 483-491.
27. Miller GA, Mendoza W, Krone MR, Meza R, Caceres CF, et al. (2004) Clients of female sex workers in Lima, Peru: a bridge population for sexually transmitted disease/HIV transmission? Sex Transm Dis 31: 337-342.
28. Goodreau SM, Goicochea LP, Sanchez J (2005) Sexual role and transmission of HIV Type 1 among men who have sex with men, in Peru. J Infect Dis 191 Suppl 1: S147-158.
29. Baggaley RF, Garnett GP, Ferguson NM (2006) Modelling the impact of antiretroviral use in resource-poor settings. PLoS Med 3: e124.
30. Patrucco R (1985) Síndrome de Inmunodeficiencia Adquirida en el Perú (Sida). Estudios Inmunológicos. Diagnóstico 16: 122-135.
31. Baggaley RF, White RG, Boily MC (2010) HIV transmission risk through anal intercourse: systematic review, meta-analysis and implications for HIV prevention. Int J Epidemiol 39: 1048-1063.
32. Vittinghoff E, Douglas J, Judson F, McKirnan D, MacQueen K, et al. (1999) Per-contact risk of human immunodeficiency virus transmission between male sexual partners. Am J Epidemiol 150: 306-311.
33. Donnell D, Baeten JM, Kiarie J, Thomas KK, Stevens W, et al. (2010) Heterosexual HIV-1 transmission after initiation of antiretroviral therapy: a prospective cohort analysis. Lancet 375: 2092-2098.
34. Mahy M, Stover J, Stanecki K, Stoneburner R, Tassie JM (2010) Estimating the impact of antiretroviral therapy: regional and global estimates of life-years gained among adults. Sex Transm Infect 86 Suppl 2: ii67-71.
35. UNAIDS (2009) Informe Nacional de UNGASS. http://www.unaids.org/es/dataanalysis/monitoringcountryprogress/2010progressreportssubmittedbycountries/file,57849,es..pdf (accessed November 2011)
36. Pinkerton SD, Abramson PR (1997) Effectiveness of condoms in preventing HIV transmission. Soc Sci Med 44: 1303-1312.
37. Weller S, Davis K (2002) Condom effectiveness in reducing heterosexual HIV transmission. Cochrane Database Syst Rev: CD003255.
38. INEI (1981) Censo de la poblacion. Peru. http://www.inei.gob.pe/ (accessed November 2011)
39. PAHO (2009) Modos de Transmisión del VIH en América Latina: Resultados de la aplicación del modelo. Lima: MINSA.
40. UNAIDS (February 2004) Costing Guidelines for HIV/AIDS Intervention Strategies For use in estimating Resource Needs, Scaling-up and Strategic Planning in the Asia/Pacific region.
41. Aldridge RW, Iglesias D, Caceres CF, Miranda JJ (2009) Determining a cost effective intervention response to HIV/AIDS in Peru. BMC Public Health 9: 352.
42. MINSA (February 2011) Escalas remunerativas. http://www.minsa.gob.pe/transparencia/prs_er.asp (accessed November 2011)
43. Gilead http://www.gilead.com/enabling_access (accessed November 2011)

44. CDC (2011) Interim Guidance: Preexposure Prophylaxis for the Prevention of HIV Infection in Men Who Have Sex with Men. MMWR Morb Mortal Wkly Rep 60: 65-68.
45. Grant R (2011) Costs estimated from the iPrEX study - Peru site.
46. Instituto Nacional de la Salud http://www.ins.gob.pe/portal/jerarquia/3/173/laboratorio-clinico/jer.173 (accessed November 2011)
47. Fox-Rushby JA, Hanson K (2001) Calculating and presenting disability adjusted life years (DALYs) in cost-effectiveness analysis. Health Policy Plan 16: 326-331.
48. Lopez de Castilla D, Verdonck K, Otero L, Iglesias D, Echevarria J, et al. (2008) Predictors of CD4+ cell count response and of adverse outcome among HIV-infected patients receiving highly active antiretroviral therapy in a public hospital in Peru. Int J Infect Dis 12: 325-331.
49. WHO Global burden of disease 2004 update: disability weights for diseases and conditions http://www.who.int/healthinfo/global_burden_disease/GBD2004_DisabilityWeights.pdf (accessed November 2011)
50. WHO-CHOICE Cost effectiveness analysis guidelines: http://www.who.int/choice/en/ (accessed November 2011)
